# Effect of Fu Zheng Jie Du Formula on outcomes in patients with severe pneumonia receiving prone ventilation: a retrospective cohort study

**DOI:** 10.3389/fphar.2024.1428817

**Published:** 2024-07-24

**Authors:** Hairong Cai, Sicong Luo, Xingui Cai, Ting Lai, Shuai Zhao, Weizhang Zhang, Jieqin Zhuang, Zhishang Li, Li Chen, Bojun Chen, Ye Ye

**Affiliations:** ^1^ The Second Clinical College of Guangzhou University of Chinese Medicine, Guangzhou, China; ^2^ Guangdong Provincial Key Laboratory of Research on Emergency in Traditional Chinese Medicine, Guangzhou, China; ^3^ Clinical Research Team of Prevention and Treatment of Cardiac Emergencies with Traditional Chinese Medicine, Guangzhou, China

**Keywords:** Fu Zheng Jie Du Formula, prone ventilation, severe pneumonia, retrospective cohort study, clinical efficacy

## Abstract

**Background:**

The effect of combining prone ventilation with traditional Chinese medicine on severe pneumonia remains unclear.

**Objective:**

To evaluate the effect of Fu Zheng Jie Du Formula (FZJDF) combined with prone ventilation on clinical outcomes in patients with severe pneumonia.

**Methods:**

This single-center retrospective cohort study included 188 severe pneumonia patients admitted to the ICU from January 2022 to December 2023. Patients were divided into an FZJD group (receiving FZJDF for 7 days plus prone ventilation) and a non-FZJD group (prone ventilation only). Propensity score matching (PSM) was performed to balance baseline characteristics. The primary outcome was the change in PaO2/FiO2 ratio after treatment. Secondary outcomes included 28-day mortality, duration of mechanical ventilation, length of ICU stay, PaCO2, lactic acid levels, APACHE II score, SOFA score, Chinese Medicine Score, inflammatory markers, and time to symptom resolution.

**Results:**

After PSM, 32 patients were included in each group. Compared to the non-FZJD group, the FZJD group showed significantly higher PaO2/FiO2 ratios, lower PaCO2, and lower lactic acid levels after treatment (*p* < 0.05 for all). The FZJD group also had significantly lower APACHE II scores, SOFA scores, Chinese Medicine Scores, and levels of WBC, PCT, hs-CRP, and IL-6 (*p* < 0.05 for all). Time to symptom resolution, including duration of mechanical ventilation, length of ICU stay, time to fever resolution, time to cough resolution, and time to resolution of pulmonary rales, was significantly shorter in the FZJD group (*p* < 0.05 for all). There was no significant difference in 28-day mortality between the two groups.

**Conclusion:**

FZJDF as an adjuvant therapy to prone ventilation can improve oxygenation and other clinical outcomes in severe pneumonia patients. Prospective studies are warranted to validate these findings.

## Introduction

Severe pneumonia remains a significant cause of morbidity and mortality worldwide, particularly among critically ill patients in intensive care units (ICUs) (Mandell et al., 2007). Despite advances in medical care, the mortality rate for severe pneumonia ranges from 20% to 50% (Fine et al., 1996; Restrepo et al., 2008). Mechanical ventilation is often required in the management of severe pneumonia, but it can lead to ventilator-induced lung injury and worsen patient outcomes (Acute Respiratory Distress Syndrome Network et al., 2000; Slutsky and Ranieri, 2013).

In recent years, prone ventilation has emerged as a promising strategy to improve oxygenation and outcomes in patients with severe pneumonia (Guérin et al., 2013). By positioning patients in the prone position, prone ventilation can optimize ventilation-perfusion matching, reduce ventilator-induced lung injury, and facilitate drainage of secretions (Gattinoni et al., 2013; Guerin et al., 2014). Several studies have demonstrated that prone ventilation can significantly improve oxygenation and reduce mortality in patients with acute respiratory distress syndrome (ARDS) (Sud et al., 2014; Mora-Arteaga et al., 2015; Munshi et al., 2017; Matthay et al., 2019; Meyer et al., 2021). However, the effect of prone ventilation on outcomes in severe pneumonia remains less well characterized (Thompson et al., 2017).

Traditional Chinese medicine (TCM) has been widely used in the management of respiratory diseases in China for thousands of years (Wu et al., 2008; Li et al., 2020; Li BH. et al., 2021; Wang et al., 2022). According to TCM theory, the pathogenesis of severe pneumonia can be attributed to the deficiency of Yang Qi and the invasion of pathogenic toxins into the lungs. The interaction between these two aspects forms a vicious cycle, leading to the aggravation of the disease and the manifestation of various symptoms, such as impaired lung function, intense pathogenic toxins, and blood stasis obstructing the collaterals (Luo et al., 2020). Fu Zheng Jie Du Formula (FZJDF), a classical TCM formula, has gained increasing attention for its potential in treating respiratory infections.Fu Zheng Jie Du Formula (FZJDF) is composed of eight herbs, *Aconiti Lateralis Radix* (Fuzi)*, Zingiberis Rhizoma* (Ganjiang)*, Glycyrrhizae Radix* (Gancao)*, Lonicerae Flos* (Jinyinhua)*, Gleditsiae Spina* (Zaojiaoci)*, Ipomoeae cairicae herba* (Wuzhaolong)*, Pogostemon cablin (Blanco) Benth. herba.* (huoxiang)*, and Citri Reticulatae Pericarpium* (Chenpi).FZJDF has the efficacy of benefiting Qi and warming Yang, resolving dampness and detoxifying toxins (Liu et al., 2022).FZJDF has been empirically employed as an adjuvant therapy for COVID-19 (Wang et al., 2021) and is particularly beneficial for patients presenting with severe deficiency syndrome complicated by pneumonia.Recent studies have highlighted the therapeutic potential of FZJDF in the management of COVID-19 (Li G. et al., 2021; Wang et al., 2021) and acute lung injury (ALI) (Liu et al., 2022), with minimal herbal toxicity and adverse effects reported. Mechanistically, FZJDF has been shown to exert its anti-inflammatory effects through the modulation of key signaling pathways and immune cell functions. For instance, FZJDF can inhibit the activation of the PI3K/Akt signaling pathway, suppress the polarization of pro-inflammatory M1 macrophages, and enhance the barrier integrity of type II alveolar epithelial cells (Liu et al., 2022). These findings provide valuable insights into the pharmacological basis of FZJDF in treating respiratory diseases.However, its efficacy in severe pneumonia, particularly in combination with prone ventilation, has not been well established. Considering the complex pathophysiology of severe pneumonia and the potential synergistic effects of FZJDF and prone ventilation, further investigation is warranted to evaluate the clinical benefits of this integrative approach.

In this study, we hypothesized that FZJDF combined with prone ventilation could improve oxygenation and clinical outcomes in patients with severe pneumonia. We conducted a retrospective cohort study to compare the outcomes between patients receiving prone ventilation with or without FZJDF. Understanding the mechanisms underlying the potential synergistic effects of FZJDF and prone ventilation, particularly in the context of the gut-lung axis, may lead to more targeted and effective therapies for this critical condition (Yu-Jie et al., 2020; Shelhamer et al., 2021).

## Methods

### Study design and setting

This was a single-center, retrospective cohort study conducted at the Emergency Intensive Care Unit (EICU) of Guangdong Provincial Hospital of Traditional Chinese Medicine. We screened all adult patients (aged ≥18 years) with severe pneumonia admitted to the EICU between January 2022 and December 2023.The diagnosis of severe pneumonia was based on the American Thoracic Society and Infectious Diseases Society of America guidelines (Haukoos and Lewis, 2015). The inclusion criteria were: 1) met the diagnostic criteria for severe pneumonia; 2) receiving mechanical ventilation within 24 h of admission; 3) received at least one session of prone ventilation; 4) adult patients aged ≥18 years.The exclusion criteria were: 1) pregnancy; 2) malignant tumors; 3) immunocompromised status; 4) received extracorporeal membrane oxygenation (ECMO); 5)history of or current malignancy with an expected survival <28 days; 6)HIV infection; 7)Severe immunodeficiency (congenital immunodeficiency, organ transplantation, autoimmune diseases, *etc.*); 8) incomplete medical records.

Propensity score matching (PSM) (Haukoos and Lewis, 2015)was performed to minimize potential confounding factors. A 1:1 nearest neighbor matching algorithm with a caliper width of 0.04 was used. The covariates included in the PSM model were: age, gender, APACHE II score, SOFA score, PaO2/FiO2 ratio, and comorbidities at admission.

**FIGURE 1:**
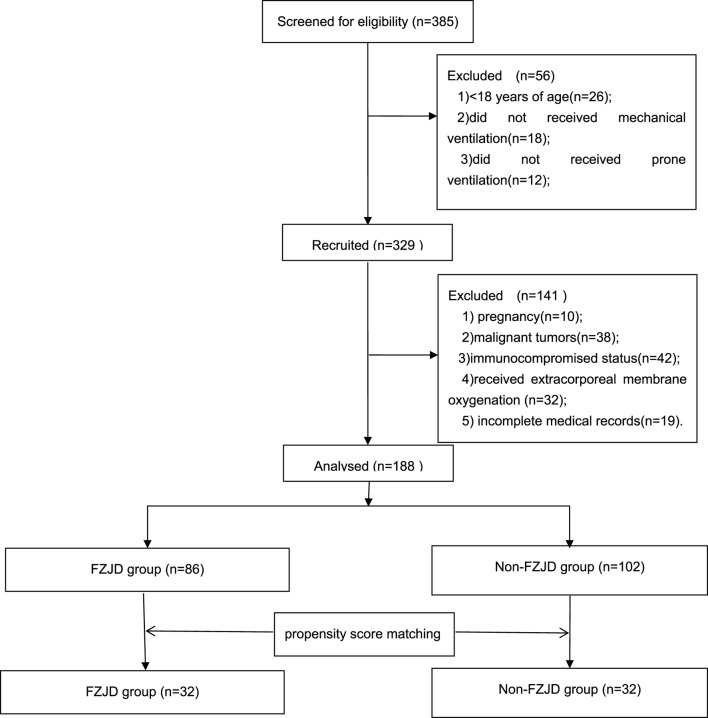
Enrollment flowchart of this study.

A total of 188 patients with severe pneumonia were admitted to the EICU during the study period. After screening for eligibility and propensity score matching, 64 patients were included in the final analysis, with 32 patients in each group (Figure 1).

### Interventions

All patients received standard treatment for severe pneumonia according to the international guidelines (Metlay et al., 2019). The antimicrobial therapy was initiated empirically and then adjusted based on the results of bacterial culture and antimicrobial susceptibility testing.For most patients with severe community-acquired pneumonia (CAP), initial treatment included a combination of β-lactam antibiotics and either macrolides or fluoroquinolones. For patients without risk factors for methicillin-resistant *Staphylococcus aureus* (MRSA) and *Pseudomonas aeruginosa*, the β-lactam could be cefotaxime, ceftriaxone. For patients with risk factors for *P. aeruginosa*, β-lactams such as piperacillin/tazobactam, Cefoperazone sodium sulbactam sodium, imipenem, or meropenem were used to achieve pathogen coverage. For patients allergic to β-lactams, aztreonam was an alternative. Empiric treatment options for MRSA included vancomycin 15 mg/kg every 12 h (adjusted based on levels) or linezolid 600 mg every 12 h. The duration of antimicrobial therapy was 7–10 days. Antiviral agents, such as oseltamivir were administered when influenza infection was suspected or confirmed. Intravenous fluids were given to maintain adequate tissue perfusion and organ function, with the type and rate of fluids adjusted based on the patient’s hemodynamic status and fluid responsiveness. Enteral nutrition was preferred and started as early as possible, with a target energy intake of 25–30 kcal/kg/day and a protein intake of 1.2–2.0 g/kg/day. Parenteral nutrition was used when enteral nutrition was contraindicated or insufficient. Organ function support, including vasopressors, inotropes, renal replacement therapy, was provided as needed based on the patient’s condition and the judgment of the attending physician. In addition to the above treatments, all patients received intravenous ambroxol (30 mg) in 0.9% sodium chloride injection, administered three times daily. Inhaled medications, including budesonide suspension (1 mg) and compound ipratropium bromide solution (2.5 mL), were given *via* nebulization three times daily, diluted with 3 mL of 0.9% sodium chloride injection.

Prone ventilation was initiated when patients met the following criteria: 1) PaO2/FiO2 ratio <150 mmHg; 2) FiO2 ≥0.6; 3) PEEP ≥5 cmH2O. The prone position was maintained for at least 16 h per day, with the duration determined by the attending physician based on the patient’s response and tolerance. The ventilator settings were adjusted to maintain a tidal volume of 6–8 mL/kg of predicted body weight, a plateau pressure ≤30 cmH2O, and a respiratory rate of 20–35 breaths/min (Albert, 2000; Mancebo et al., 2006; Guérin, 2014). The FiO2 and PEEP were titrated to target a PaO2 of 60–80 mmHg or SpO2 of 88%–95% (Petrone et al., 2021). Lung-protective ventilation strategies, including low tidal volume, permissive hypercapnia, and recruitment maneuvers, were applied to minimize ventilator-induced lung injury.

Patients in the FZJD group additionally received FZJDF, which was prepared by the hospital’s TCM pharmacy. The composition of FZJDF was as follows: *Aconiti Lateralis Radix* (Fuzi) 10g*, Zingiberis Rhizoma* (Ganjiang) 15g, *Glycyrrhizae Radix* (Gancao) 20g*, Lonicerae Flos* (Jinyinhua) 15g*, Gleditsiae Spina* (Zaojiaoci) 15g*, Ipomoeae cairicae herba* (Wuzhaolong) 20g*, P. cablin (Blanco) Benth. herba* (huoxiang) 15g*, and Citri Reticulatae Pericarpium* (Chenpi) 5 g.Source and Authentication:All herbs used in the FZJDF were provided by the Chinese Medicine Pharmacy of Guangdong Provincial Hospital of Chinese Medicine (The second Clinical Medical College of Guangzhou University of Chinese Medicine). The herbs were purchased from Kangmei Pharmaceutical Co., Ltd. Authentication of the herbs was performed by senior licensed Traditional Chinese Medicine pharmacists.Preparation Method:The FZJDF was prepared using a standardized method combining traditional Chinese medicine practices with modern pharmaceutical techniques. Herbs, sourced from the Chinese Medicine Pharmacy of Guangdong Provincial Hospital of Chinese Medicine and authenticated by senior licensed Traditional Chinese Medicine pharmacists, were combined according to Table 1. The mixture underwent two sequential decoctions: first in 1,000 mL of purified water for 30 min, then in 800 mL for 20 min, both at simmering temperature. The combined decoctions were concentrated to approximately 200 mL, yielding a 4:1 (w/v) concentration ratio of raw materials to final product. Each 200 mL dose, representing the extract from 90 g of raw herbs, was sealed in sterile containers and stored at 4°C with a 7-day shelf life. Quality control measures included organoleptic evaluation and sample retention for each batch. This rigorous process ensured consistency and potency, providing a reliable basis for clinical application.Quality Control and Batch Consistency Measures:To ensure consistency and quality across different batches of FZJDF, a comprehensive set of control measures was implemented. All herbs, sourced exclusively from Kangmei Pharmaceutical Co., Ltd., were authenticated by senior licensed Traditional Chinese Medicine pharmacists through organoleptic evaluation in accordance with the Chinese Pharmacopoeia standards. The preparation adhered to a standardized protocol, including precise weighing of herbs (±1% tolerance), controlled decoction parameters, and consistent concentration procedures to achieve a final volume of 200 mL per dose (±2% deviation). Each batch underwent rigorous organoleptic assessment for color, odor, and taste, as well as physical inspection for viscosity and absence of visible contaminants. The density of the final preparation was measured using a calibrated hydrometer (acceptable range: 1.05–1.10 g/mL). Batch-to-batch consistency was primarily ensured through strict adherence to the standardized preparation protocol and comprehensive documentation of each batch, including raw material sources, preparation details, and pharmacist observations. Prepared decoctions were stored at 4°C and used within a 7-day period. While quantitative analysis of individual compounds was not performed for each batch in this clinical setting, the rigorous standardization of the preparation process, combined with organoleptic and physical evaluations, provided a practical approach to maintaining consistent quality and composition. This method balances traditional practices with modern quality assurance principles, ensuring reliable production of FZJDF for clinical use.

**TABLE 1:** Composition of FZJDF.

Herb name	Herbs name in Latin	Abbreviation	Family	Dosage (g)
Fuzi	*Aconiti Lateralis Radix*	FZ	Ranunculaceae	10
Ganjiang	*Zingiberis Rhizoma*	GJ	Zingiberaceae	15
Wuzhaolong	*Ipomoeae cairicae herba*	WZL	Convolvulaceae	20
Zaojiaoci	*Gleditsiae Spina*	ZJC	Fabaceae	15
Jingyinhua	*Lonicerae Flos*	JYH	Caprifoliaceae	15
Huoxiang	*Pogostemon cablin (Blanco) Benth. Herba*	HX	Lamiaceae	15
Chenpi	*Citri Reticulatae Pericarpium*	CP	Rutaceae	5
Gancao	*Glycyrrhizae Radix*	GC	Fabaceae	20

Patients in the non-FZJD group received standard treatment and prone ventilation without FZJDF. The use of other TCM therapies, such as herbal injections, oral herbal preparations, or external application of herbal medicines, was not allowed in either group during the study period. The concomitant use of Western medications was permitted as clinically indicated and recorded in the case report form.

### Outcomes

The primary outcome was the change in PaO2/FiO2 ratio from baseline to day 7. The secondary outcomes included:

1) 28-day mortality;
2) Duration of mechanical ventilation;
3) Length of ICU stay;
4) PaCO2 and lactic acid levels on days 1 and 7;
5) APACHE II score and SOFA score on days 1 and 7;
6) Chinese Medicine Score (Jiang et al., 2012) on days 1 and 7; The Chinese Medicine Score was a self-designed scoring system based on TCM syndrome differentiation, which evaluated the severity of Yang deficiency, Qi deficiency, Damp-Heat and Evil-Toxins. Each item was scored from 0 (none) to 3 (severe), with a total score ranging from 0 to 12. Higher scores indicated more severe TCM syndromes.
7) Inflammatory markers (WBC, PCT, Hs-CRP, IL-6) on days 1 and 7;
8) Time to fever resolution;
9) Time to cough resolution;
10) Time to resolution of pulmonary rales.

### Data collection

Demographic and clinical data were extracted from the electronic medical records by trained research staff using a standardized data collection form. The collected data included age, sex, comorbidities (cerebral infarction, hypertension, coronary heart disease, diabetes, chronic kidney disease, chronic lung disease), laboratory tests (white blood cell count, lymphocyte count, C-reactive protein, procalcitonin), and clinical scores (Acute Physiology and Chronic Health Evaluation II [APACHE II] score, Sequential Organ Failure Assessment [SOFA] score).Data on interventions, including mechanical ventilation parameters (ventilation mode, tidal volume, positive end-expiratory pressure [PEEP], fraction of inspired oxygen [FiO2]), prone positioning, and medication administration (antibiotics, antivirals, glucocorticoids) were recorded daily.The primary outcome, PaO2/FiO2 ratio, was recorded at baseline and on day 7 after enrollment. Secondary outcomes, including inflammatory markers (interleukin-6 [IL-6], tumor necrosis factor-α [TNF-α]) and Chinese medicine syndrome scores, were also collected at these two time points.All data were entered into a secure electronic database and checked for accuracy and completeness by a second researcher. Any discrepancies were resolved through discussion with the principal investigator. The database was backed up regularly to ensure data integrity and security.

### Statistical analysis

Missing data were first analyzed for their missing mechanisms, and the possibility of non-random missingness was excluded. For data with missing completely at random (MCAR) or missing at random (MAR), multiple imputation using chained equations (MICE) was performed to generate five complete datasets. Analyses were then conducted on each imputed dataset, and the results were combined using Rubin’s rules. All missing data analyses were completed before the main outcome analyses.Continuous variables were expressed as mean ± standard deviation or median (interquartile range) and compared using the independent sample *t*-test or Mann-Whitney *U* test, as appropriate. Categorical variables were presented as numbers (percentages) and compared using the chi-square test or Fisher’s exact test.To minimize potential confounding factors, propensity score matching (PSM) was performed. The propensity score was estimated using a multivariable logistic regression model, with treatment assignment as the dependent variable and baseline characteristics as covariates. The covariates included in the propensity score model were age, gender, APACHE II score, SOFA score, PaO2/FiO2 ratio, and comorbidities at admission.Patients in the FZJD group were matched 1:1 to those in the non-FZJD group using the nearest neighbor matching algorithm with a caliper width of 0.04. The balance of baseline covariates between the two groups before and after PSM was assessed using standardized mean differences (SMDs), with an absolute SMD <0.1 indicating a negligible difference.The primary outcome, the change in PaO2/FiO2 ratio, and secondary outcomes, including inflammatory markers and Chinese medicine syndrome scores, were compared between the two groups using the independent sample *t*-test or Mann-Whitney *U* test, as appropriate. The paired *t*-test or Wilcoxon signed-rank test was used to compare the changes in these outcomes within each group before and after treatment.All statistical analyses were performed using SPSS 26.0 (IBM Corp., Armonk, NY, United States of America) and R 4.0.3 (R Foundation for Statistical Computing, Vienna, Austria). A two-sided *p*-value <0.05 was considered statistically significant.

## Results

### Composition and active ingredients of FZJDF

FZJDF consists of eight traditional Chinese herbs, as detailed in Table 1. To elucidate its potential mechanism of action, we performed a network pharmacology analysis.Through screening of the TCMSP and Herb databases, we identified 172 potential active ingredients in FZJDF (Table 2). These active ingredients were predicted to act on 462 gene targets (Supplementary Table S1).Using the GeneCards and OMIM databases, we identified 4,483 genes associated with severe pneumonia. By comparing the herb-related targets with disease-related targets, we identified 221 common targets, which may be crucial for the therapeutic effects of FZJDF (Supplementary Table S1).

**TABLE 2:** Metabolite of FZJDF.

Metabolite of Chinese herbs	Herb	Diabetes
sitosterol	CP	TCMSP
naringenin	CP	TCMSP
5,7-dihydroxy-2-(3-hydroxy-4-methoxyphenyl)chroman-4-one	CP	TCMSP
Citromitin	CP	TCMSP
nobiletin	CP	TCMSP
11,14-eicosadienoic acid	FZ	TCMSP
Delphin_qt	FZ	TCMSP
Deltoin	FZ	TCMSP
Demethyldelavaine A	FZ	TCMSP
Demethyldelavaine B	FZ	TCMSP
Deoxyandrographolide	FZ	TCMSP
karakoline	FZ	TCMSP
Karanjin	FZ	TCMSP
Neokadsuranic acid B	FZ	TCMSP
benzoylnapelline	FZ	TCMSP
6-Demethyldesoline	FZ	TCMSP
deoxyaconitine	FZ	TCMSP
(R)-Norcoclaurine	FZ	TCMSP
ignavine	FZ	TCMSP
isotalatizidine	FZ	TCMSP
jesaconitine	FZ	TCMSP
Carnosifloside I_qt	FZ	TCMSP
hypaconitine	FZ	TCMSP
1-Monolinolein	GJ	TCMSP
Sexangularetin	GJ	TCMSP
beta-sitosterol	GJ	TCMSP
Diop	HX	TCMSP
Genkwanin	HX	TCMSP
patchoulan 1,12-diol	HX	TCMSP
pachypodol	HX	TCMSP
5-Hydroxy-7,4′-dimethoxyflavanon	HX	TCMSP
irisolidone	HX	TCMSP
phenanthrone	HX	TCMSP
quercetin 7-O-β-D-glucoside	HX	TCMSP
Acanthoside B	HX	TCMSP
3,23-dihydroxy-12-oleanen-28-oic acid	HX	TCMSP
quercetin	HX	TCMSP
Mandenol	JYH	TCMSP
Ethyl linolenate	JYH	TCMSP
phytofluene	JYH	TCMSP
Eriodyctiol (flavanone)	JYH	TCMSP
(−)-(3R,8S,9R,9aS,10aS)-9-ethenyl-8-(beta-D-glucopyranosyloxy)-2,3,9,9a,10,10a-hexahydro-5-oxo-5H,8H-pyrano [4,3-d]oxazolo [3,2-a]pyridine-3-carboxylic acid_qt	JYH	TCMSP
secologanic dibutylacetal_qt	JYH	TCMSP
beta-carotene	JYH	TCMSP
ZINC03978781	JYH	TCMSP
Chryseriol	JYH	TCMSP
kryptoxanthin	JYH	TCMSP
7-epi-Vogeloside	JYH	TCMSP
Caeruloside C	JYH	TCMSP
Centauroside_qt	JYH	TCMSP
Ioniceracetalides B_qt	JYH	TCMSP
XYLOSTOSIDINE	JYH	TCMSP
dinethylsecologanoside	JYH	TCMSP
kaempferol	JYH	TCMSP
Stigmasterol	JYH	TCMSP
luteolin	JYH	TCMSP
alizarin	WZL	Herb
amygdalin	WZL	Herb
citric acid	WZL	Herb
dihydromollugin	WZL	Herb
ergocornine	WZL	Herb
ergocorninine	WZL	Herb
furomollugin	WZL	Herb
l-arctigenin	WZL	Herb
malic acid	WZL	Herb
munjistin	WZL	Herb
muricatin b	WZL	Herb
muricatocin a	WZL	Herb
(?)-nortrachelogenin	WZL	Herb
oleanolic acid	WZL	Herb
prunasin	WZL	Herb
purpurin	WZL	Herb
purpuroxanthin	WZL	Herb
ruberythric acid	WZL	Herb
rubiadin	WZL	Herb
rubimaillin	WZL	Herb
succine acid	WZL	Herb
tartaric acid	WZL	Herb
trachelogenin	WZL	Herb
tracheloside	WZL	Herb
fisetin	ZJC	TCMSP
Fustin	ZJC	TCMSP
(−)-taxifolin	ZJC	TCMSP
Stigmast-4-ene-3,6-dione	ZJC	TCMSP
ent-Epicatechin	ZJC	TCMSP
Inermine	GC	TCMSP
DFV	GC	TCMSP
Mairin	GC	TCMSP
Glycyrol	GC	TCMSP
Jaranol	GC	TCMSP
Medicarpin	GC	TCMSP
isorhamnetin	GC	TCMSP
Lupiwighteone	GC	TCMSP
7-Methoxy-2-methyl isoflavone	GC	TCMSP
formononetin	GC	TCMSP
Calycosin	GC	TCMSP
(2S)-2-[4-hydroxy-3-(3-methylbut-2-enyl)phenyl]-8,8-dimethyl-2,3-dihydropyrano [2,3-f]chromen-4-one	GC	TCMSP
euchrenone	GC	TCMSP
glyasperin B	GC	TCMSP
glyasperin F	GC	TCMSP
Glyasperin C	GC	TCMSP
Isotrifoliol	GC	TCMSP
(E)-1-(2,4-dihydroxyphenyl)-3-(2,2-dimethylchromen-6-yl)prop-2-en-1-one	GC	TCMSP
kanzonols W	GC	TCMSP
(2S)-6-(2,4-dihydroxyphenyl)-2-(2-hydroxypropan-2-yl)-4-methoxy-2,3-dihydrofuro [3,2-g]chromen-7-one	GC	TCMSP
Semilicoisoflavone B	GC	TCMSP
Glepidotin A	GC	TCMSP
Glepidotin B	GC	TCMSP
Phaseolinisoflavan	GC	TCMSP
Glypallichalcone	GC	TCMSP
8-(6-hydroxy-2-benzofuranyl)-2,2-dimethyl-5-chromenol	GC	TCMSP
Licochalcone B	GC	TCMSP
licochalcone G	GC	TCMSP
3-(2,4-dihydroxyphenyl)-8-(1,1-dimethylprop-2-enyl)-7-hydroxy-5-methoxy-coumarin	GC	TCMSP
Licoricone	GC	TCMSP
Gancaonin A	GC	TCMSP
Gancaonin B	GC	TCMSP
licorice glycoside E	GC	TCMSP
3-(3,4-dihydroxyphenyl)-5,7-dihydroxy-8-(3-methylbut-2-enyl)chromone	GC	TCMSP
5,7-dihydroxy-3-(4-methoxyphenyl)-8-(3-methylbut-2-enyl)chromone	GC	TCMSP
2-(3,4-dihydroxyphenyl)-5,7-dihydroxy-6-(3-methylbut-2-enyl)chromone	GC	TCMSP
Glycyrin	GC	TCMSP
Licocoumarone	GC	TCMSP
Licoisoflavone	GC	TCMSP
Licoisoflavone B	GC	TCMSP
licoisoflavanone	GC	TCMSP
shinpterocarpin	GC	TCMSP
(E)-3-[3,4-dihydroxy-5-(3-methylbut-2-enyl)phenyl]-1-(2,4-dihydroxyphenyl)prop-2-en-1-one	GC	TCMSP
liquiritin	GC	TCMSP
licopyranocoumarin	GC	TCMSP
Glyzaglabrin	GC	TCMSP
Glabridin	GC	TCMSP
Glabranin	GC	TCMSP
Glabrene	GC	TCMSP
Glabrone	GC	TCMSP
1,3-dihydroxy-9-methoxy-6-benzofurano [3,2-c]chromenone	GC	TCMSP
1,3-dihydroxy-8,9-dimethoxy-6-benzofurano [3,2-c]chromenone	GC	TCMSP
Eurycarpin A	GC	TCMSP
glycyroside	GC	TCMSP
(−)-Medicocarpin	GC	TCMSP
Sigmoidin-B	GC	TCMSP
(2R)-7-hydroxy-2-(4-hydroxyphenyl)chroman-4-one	GC	TCMSP
(2S)-7-hydroxy-2-(4-hydroxyphenyl)-8-(3-methylbut-2-enyl)chroman-4-one	GC	TCMSP
Isoglycyrol	GC	TCMSP
Isolicoflavonol	GC	TCMSP
HMO	GC	TCMSP
1-Methoxyphaseollidin	GC	TCMSP
Quercetin der	GC	TCMSP
3′-Hydroxy-4′-O-Methylglabridin	GC	TCMSP
licochalcone a	GC	TCMSP
3′-Methoxyglabridin	GC	TCMSP
2-[(3R)-8,8-dimethyl-3,4-dihydro-2H-pyrano [6,5-f]chromen-3-yl]-5-methoxyphenol	GC	TCMSP
Inflacoumarin A	GC	TCMSP
icos-5-enoic acid	GC	TCMSP
Kanzonol F	GC	TCMSP
6-prenylated eriodictyol	GC	TCMSP
7,2′,4′-trihydroxy-5-methoxy-3-arylcoumarin	GC	TCMSP
7-Acetoxy-2-methylisoflavone	GC	TCMSP
8-prenylated eriodictyol	GC	TCMSP
gadelaidic acid	GC	TCMSP
Vestitol	GC	TCMSP
Gancaonin G	GC	TCMSP
Gancaonin H	GC	TCMSP
Licoagrocarpin	GC	TCMSP
Glyasperins M	GC	TCMSP
Glycyrrhiza flavonol A	GC	TCMSP
Licoagroisoflavone	GC	TCMSP
18α-hydroxyglycyrrhetic acid	GC	TCMSP
Odoratin	GC	TCMSP
Phaseol	GC	TCMSP
Xambioona	GC	TCMSP
dehydroglyasperins C	GC	TCMSP

### Baseline characteristics of patients before propensity score matching

A total of 188 patients with severe pneumonia were admitted to the EICU during the study period. After screening for eligibility and propensity score matching, 64 patients were included in the final analysis, with 32 patients in each group. The baseline characteristics of the two groups were well balanced after matching (Table 3).

**TABLE 3:** Presents the baseline characteristics of the two groups.

Characteristic	FZJD group (n = 86)	Non-FZJD group (n = 102)	Statistical values	*P*
Sex,n (%)				
Male	58 (67.442)	70 (68.627)	0.030	0.862
Female	28 (32.558)	32 (31.373)		
cerebral infarction,n (%)	19 (22.093)	20 (19.608)	0.175	0.675
high blood pressure,n (%)	23 (26.744)	12 (11.765)	6.910	0.009
coronary heart disease,n (%)	20 (23.255)	29 (28.431)	1.533	0.216
diabetes,n (%)	29 (33.721)	36 (35.294)	0.051	0.821
chronic kidney disease,n (%)	6 (6.977)	10 (9.804)	0.479	0.489
Chronic lung disease,n (%)	10 (11.628)	15 (14.706)	0.383	0.536
age,years	75.953 ± 14.333	70.137 ± 15.602	2.628	0.009
heat generation time, days	4.000 [3.000,5.000]	5.000 [4.000,6.000]	−1.621	0.097
T,°C	39.125 ± 4.861	39.500 ± 3.332	−0.601	0.548
SBP,mmHg	107.221 ± 20.651	97.392 ± 19.347	3.347	<0.001
DBP,mmHg	76.616 ± 14.626	72.451 ± 10.804	2.174	0.031
HR,bpm	113.965 ± 22.285	121.225 ± 22.971	−2.177	0.031
R,bpm	26.977 ± 3.267	27.490 ± 3.469	−1.033	0.303
SPO2,%	84.698 ± 6.595	84.059 ± 7.222	0.625	0.533
BMI,kg/m^2^	28.384 ± 3.383	27.480 ± 3.572	1.760	0.080
CPIS, scores	9.000 [7.000,10.000]	8.000 [7.000,10.000]	1.158	0.243

### Baseline characteristics of patients after propensity score matching

To reduce confounding bias arising from differences in baseline characteristics between the two groups, propensity score matching (PSM) was performed using 1:1 nearest-neighbor matching. A caliper value of 0.04 was used. After PSM, 32 patients were matched from each group. Table 4 presents the baseline characteristics of the two matched groups. No significant differences were observed in baseline characteristics between the two groups after matching.

**TABLE 4:** Baseline characteristics of patients after propensity score matching.

Characteristic	FZJD group (n = 32)	Non-FZJD group (n = 32)	Statistical values	*P*
Sex,n (%)				
Male	18 (56.250)	17 (53.125)	0.063	0.802
Female	14 (43.750)	15 (46.875)		
cerebral infarction,n (%)	10 (31.250)	9 (28.125)	0.075	0.784
high blood pressure,n (%)	9 (28.125)	10 (31.250)	0.075	0.784
coronary heart disease,n (%)	9 (28.125)	9 (28.125)	0.000	1.000
diabetes,n (%)	11 (34.375)	9 (28.125)	0.291	0.590
chronic kidney disease,n (%)	4 (12.500)	3 (9.375)	0.000	1.000
Chronic lung disease,n (%)	5 (15.625)	7 (21.875)	0.410	0.522
age,years	76.314 ± 12.205	72.938 ± 10.565	−1.251	0.215
heat generation time, days	4.000 [3.000,4.000]	4.000 [3.000,4.000]	−0.832	0.373
T,°C	38.804 [38.242,39.561]	38.839 [38.593,39.255]	0.752	0.456
SBP,mmHg	120.125 ± 12.869	115.219 ± 13.747	−1.451	0.152
DBP,mmHg	77.500 ± 11.164	78.250 ± 7.814	0.306	0.760
HR,bpm	122.281 ± 19.189	117.625 ± 16.101	−1.035	0.305
R,bpm	27.219 ± 4.519	27.906 ± 5.083	0.563	0.576
SPO2,%	85.531 ± 10.695	86.906 ± 6.939	0.601	0.551
BMI,kg/m^2^	27.761 ± 6.292	27.439 ± 4.223	−0.237	0.814
CPIS, scores	8.000 [7.000,11.000]	8.000 [6.000,10.000]	−0.483	0.630

### 28-Day mortality

No significant difference in 28-day mortality was observed between the FZJD group (5 [15.625%]) and the non-FZJD group (7 [17.910%]) (*p* = 0.522) (Table 5).

**TABLE 5:** 28-Day mortality.

Characteristic	FZJD group (n = 32)	Non-FZJD group (n = 32)	Statistical values	*P*
28-d Death,n (%)	5 (15.625)	7 (17.910)	0.410	0.522

### Blood gas analysis

**FIGURE 2:**
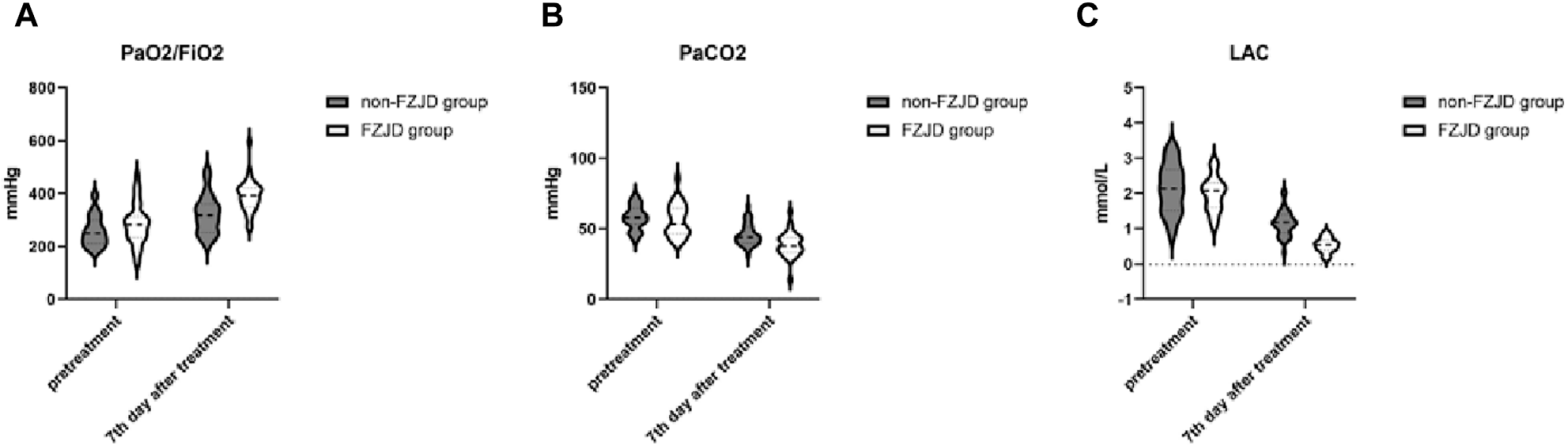
Changes in blood gas parameters over the treatment period. **(A)** PaO2/FiO2 ratio. **(B)** Partial pressure of arterial carbon dioxide (PaCO2). **(C)** Lactic acid (LAC) levels.

After treatment, compared to the non-FZJD group, the FZJD group had a significantly higher PaO2/FiO2 ratio (391.281 ± 61.727 vs. 321.344 ± 72.050 mmHg, *p* < 0.001), lower PaCO2 (38.844 ± 8.650 vs. 45.844 ± 7.807 mmHg, *p* = 0.001), and lower lactic acid levels (0.538 ± 0.207 vs. 1.165 ± 0.362 mmol/L, *p* < 0.001) (Table 6; Figure 2).

**TABLE 6:** Blood gas analysis.

Characteristic	FZJD group (n = 32)	Non-FZJD group (n = 32)	Statistical values	*P*
PaO2/FiO2,mmHg				
pretreatment	284.531 ± 77.246	258.719 ± 56.855	−1.498	0.139
7th day after treatment	391.281 ± 61.727	321.344 ± 72.050	−4.104	<0.001
PaCO2,mmHg				
pretreatment	56.031 ± 11.126	57.875 ± 8.241	0.741	0.461
7th day after treatment	38.844 ± 8.650	45.844 ± 7.807	3.345	0.001
LAC,mmol/L				
pretreatment	1.988 ± 0.492	2.143 ± 0.687	1.018	0.313
7th day after treatment	0.538 ± 0.207	1.165 ± 0.362	8.383	<0.001

### Clinical scores

**FIGURE 3:**
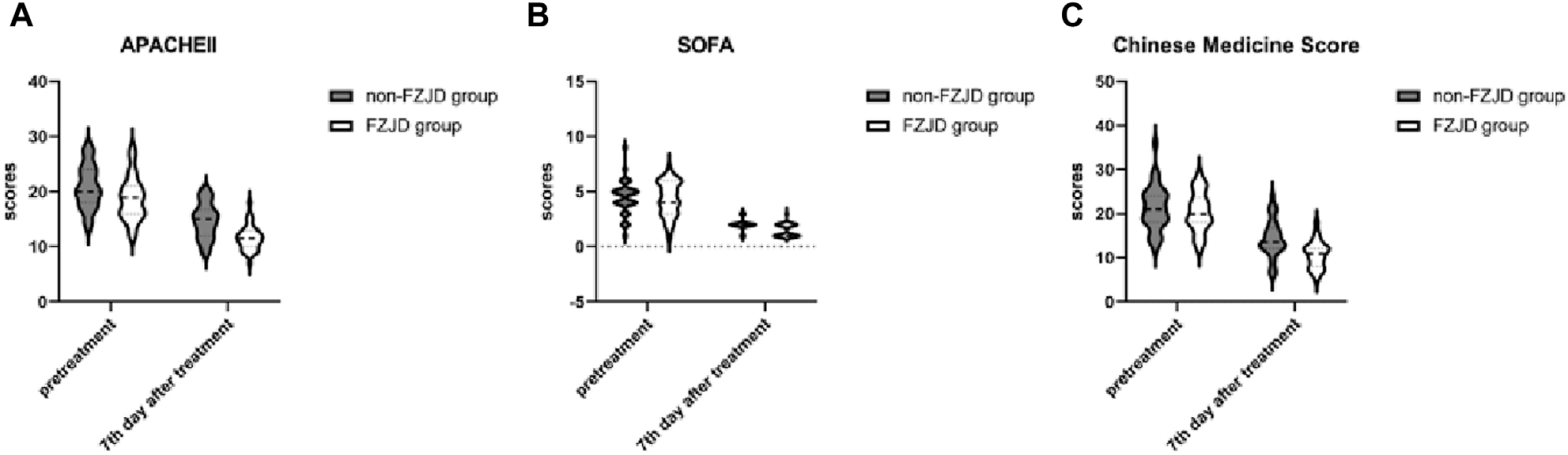
Changes in severity scores during treatment. **(A)** APACHE II (Acute Physiology and Chronic Health Evaluation II) score. **(B)** SOFA (Sequential Organ Failure Assessment) score. **(C)** Chinese Medicine Score.

The FZJD group had significantly lower APACHE II score (mean ± SD, 11.625 ± 2.274 vs. 14.719 ± 3.145 scores; *p* < 0.001), SOFA score (median [IQR], 1.000 [1.000,2.000] vs. 2.00 [2.00, 2.00] scores; *p* < 0.001), and Chinese Medicine Score (mean ± SD, 10.688 ± 2.984 vs. 14.344 ± 4.254 scores; *p* < 0.001) compared to the non-FZJD group after treatment (Table 7; Figure 3).

**TABLE 7:** Clinical scores.

Characteristic	FZJD group (n = 32)	Non-FZJD group (n = 32)	Statistical values	*P*
APACHEⅡ,scores				
pretreatment	18.781 ± 3.974	20.844 ± 3.850	2.075	0.042
7th day after treatment	11.625 ± 2.274	14.719 ± 3.145	4.438	<0.001
SOFA, scores				
pretreatment	4.000 [3.000,6.000]	5.000 [4.000,5.000]	0.040	0.973
7th day after treatment	1.000 [1.000,2.000]	2.000 [2.000,2.000]	3.216	<0.001
Chinese Medicine Score, scores				
pretreatment	20.406 ± 4.422	21.219 ± 5.097	0.670	0.505
7th day after treatment	10.688 ± 2.984	14.344 ± 4.254	3.918	<0.001

### Inflammatory markers

**FIGURE 4:**
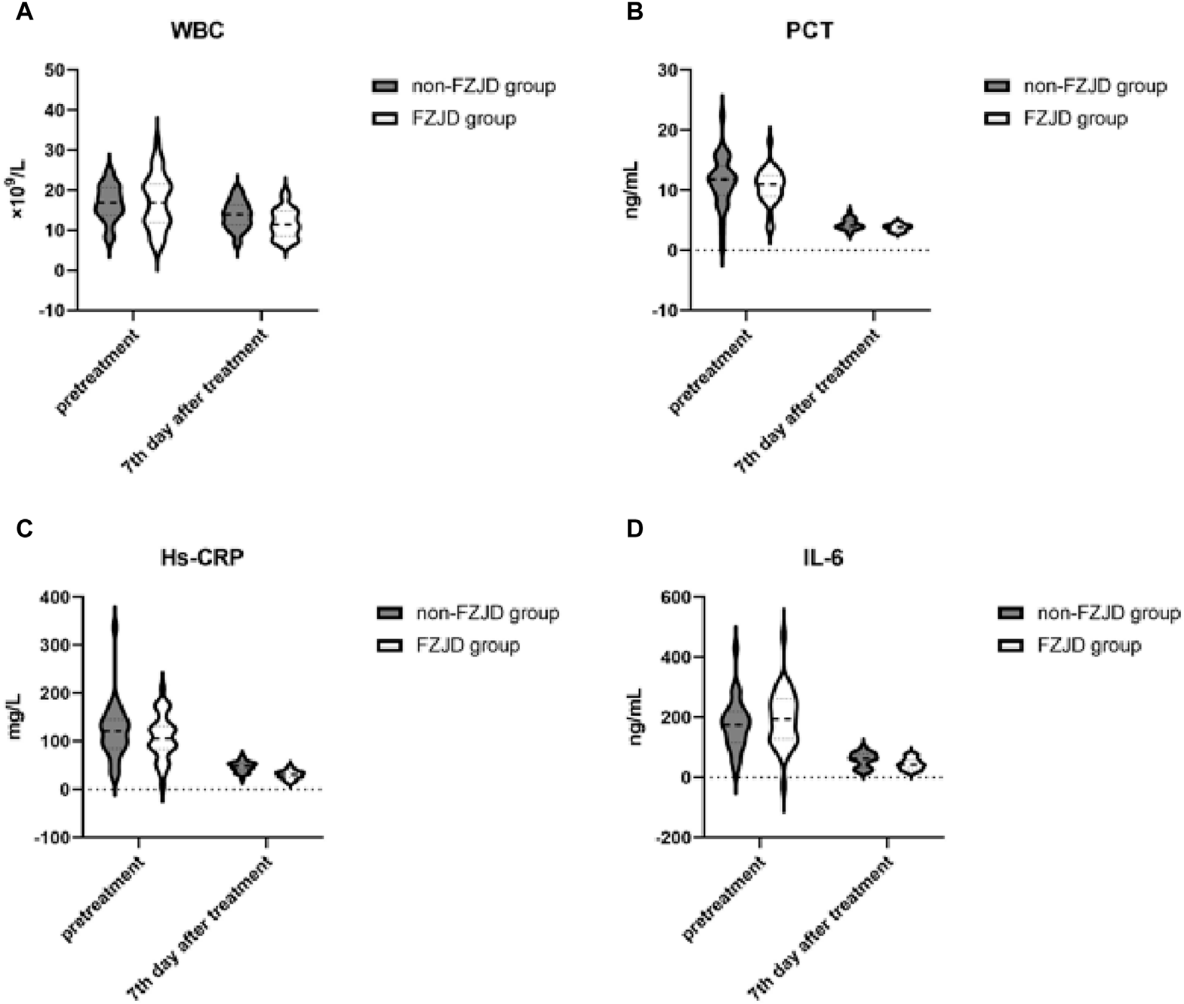
Changes in inflammatory markers during treatment. **(A)** White Blood Cell (WBC) count. **(B)** Procalcitonin (PCT) levels. **(C)** High-sensitivity C-Reactive Protein (Hs-CRP) concentration. **(D)** Interleukin-6 (IL-6) levels.

After treatment, the FZJD group had significantly lower levels of WBC (mean ± SD, 11.763 ± 3.560 vs. 13.778 ± 3.475 ×10^9^/L; *p* = 0.028), PCT (mean ± SD, 3.781 ± 0.548 vs. 4.317 ± 0.868 ng/mL; *p* = 0.005), Hs-CRP (mean ± SD, 30.172 ± 9.057 vs. 47.453 ± 10.662 mg/L; *p* < 0.001), and IL-6 (mean ± SD, 44.864 ± 18.460 vs. 59.092 ± 24.067 ng/mL; *p* = 0.011 compared to the non-FZJD group (Table 8; Figure 4).

**TABLE 8:** Inflammatory markers.

Characteristic	FZJD group (n = 32)	Non-FZJD group (n = 32)	Statistical values	*P*
WBC,×10^9^/L				
pretreatment	17.178 ± 6.654	16.836 ± 4.399	−0.238	0.813
7th day after treatment	11.763 ± 3.560	13.778 ± 3.475	2.255	0.028
PCT,ng/mL				
pretreatment	10.589 ± 2.907	11.378 ± 4.189	0.862	0.392
7th day after treatment	3.781 ± 0.548	4.317 ± 0.868	2.907	0.005
Hs-CRP,mg/L				
pretreatment	109.670 [86.524,131.148]	122.622 [87.997,142.407]	0.819	0.417
7th day after treatment	30.172 ± 9.057	47.453 ± 10.662	6.878	<0.001
IL-6,ng/mL				
pretreatment	195.511 ± 93.130	174.695 ± 81.946	−0.934	0.354
7th day after treatment	44.864 ± 18.460	59.092 ± 24.067	2.612	0.011

### Symptom relief time

**FIGURE 5:**
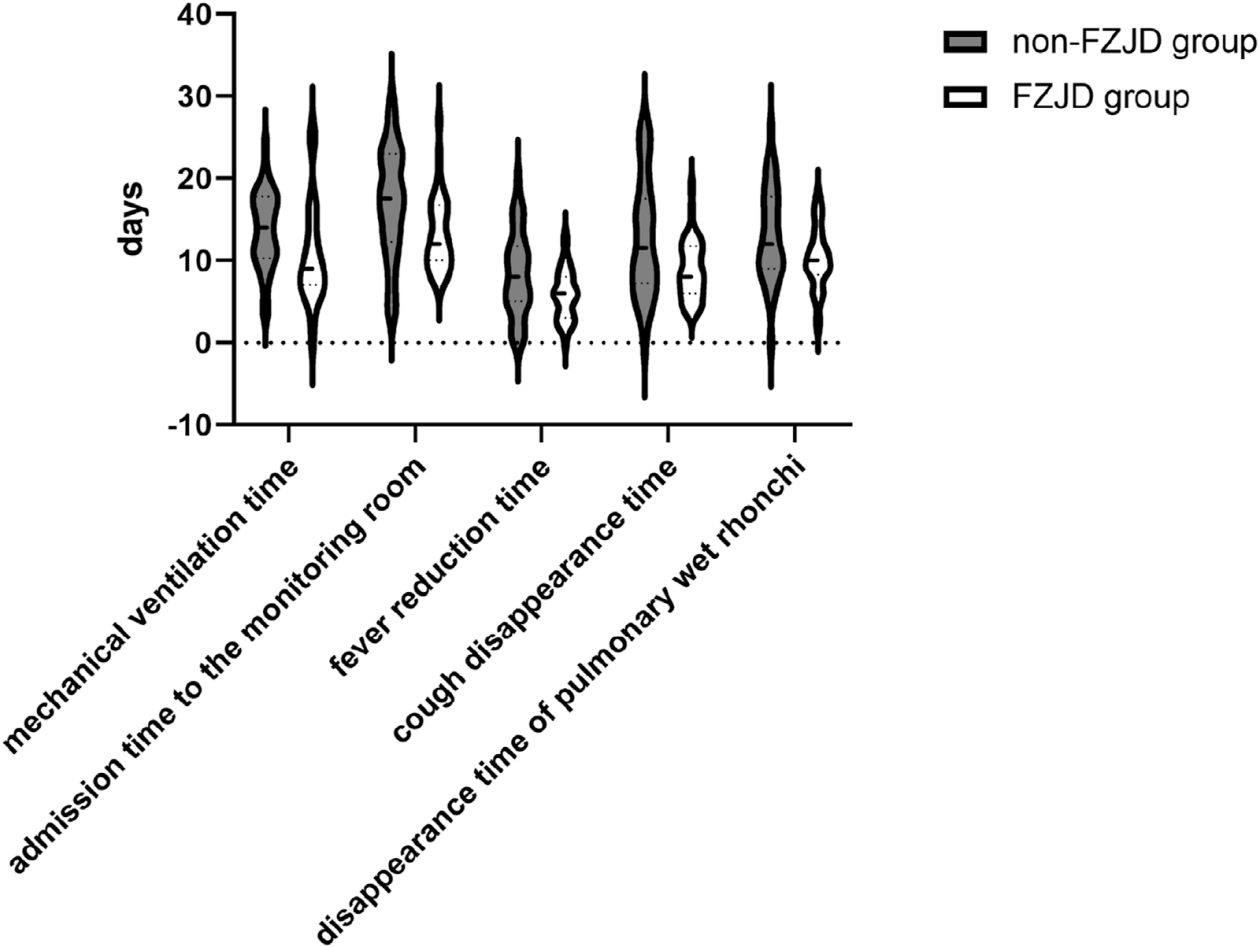
Symptom relief time.

The FZJD group had significantly shorter mechanical ventilation time (median [IQR], 9.000 [7.000,13.000] vs. 14.000 [11.000,17.000] days; *p* = 0.002), ICU stay (median [IQR], 12.000 [10.000,16.000] vs. 18.000 [13.000,23.000] days; *p* = 0.007), fever duration (mean ± SD, 5.875 ± 3.049 vs. 8.375 ± 5.079 days; *p* = 0.025), cough disappearance time (mean ± SD, 8.906 ± 3.617 vs. 12.688 ± 7.086 days; *p* = 0.008), and disappearance time of pulmonary rales (mean ± SD, 10.156 ± 3.784 vs. 13.063 ± 5.722 days; *p* = 0.022) compared to the control group (Table 9; Figure 5).

**TABLE 9:** Symptom Relief time.

Characteristic	FZJD group (n = 32)	Non-FZJD group (n = 32)	Statistical values	*P*
mechanical ventilation time, days	9.000 [7.000,13.000]	14.000 [11.000,17.000]	2.732	0.006
admission time to the monitoring room, days	12.000 [10.000,16.000]	18.000 [13.000,23.000]	2.652	0.008
fever reduction time, days	5.875 ± 3.049	8.375 ± 5.079	2.350	0.023
cough disappearance time, days	8.906 ± 3.617	12.688 ± 7.086	2.646	0.011
disappearance time of pulmonary wet rhonchi, days	10.156 ± 3.784	13.063 ± 5.722	2.359	0.022

## Discussion

Before delving into a detailed discussion of our findings and their implications, we present a glossary of key terms used in this study (Table 10). Clarifying these terms will facilitate a better understanding of the subsequent discussion. In the theoretical framework of Traditional Chinese Medicine (TCM), Fu Zheng Jie Du Formula is categorized as a combination of “qi-tonifying and yang-warming” and “heat-clearing and toxin-eliminating” herbs. This classification reflects the formula’s dual therapeutic approach within TCM theory. It is important to note that while these traditional concepts provide valuable historical context and insight into the formula’s intended effects, they cannot be directly evaluated or validated through contemporary biological science methods. These TCM classifications do not correspond directly to modern biomedical understandings of physiology and pharmacology. Nevertheless, they remain relevant as background information, offering perspective on the theoretical basis that guided the formula’s composition and its historical application in TCM practice. The clinical efficacy and mechanisms of action of FZJDF are subject to ongoing scientific investigation using evidence-based research methodologies, which aim to elucidate its effects in terms of modern medical understanding.In this retrospective cohort study, we found that the combination of FZJDF with conventional treatment and prone ventilation significantly improved oxygenation, reduced inflammatory response, and shortened the duration of mechanical ventilation and ICU stay in patients with severe pneumonia. Compared to the non-FZJD group, the FZJD group showed a significantly higher PaO2/FiO2 ratio, lower PaCO2, and lower lactic acid levels after treatment. The FZJD group also had significantly lower APACHE II score, SOFA score, Chinese Medicine Score, and levels of inflammatory markers, including WBC, PCT, Hs-CRP, and IL-6. These findings suggest that FZJDF may be an effective adjuvant therapy for severe pneumonia, potentially improving patient outcomes (Ren et al., 2021). Given the high mortality and complex pathophysiology of severe pneumonia, identifying effective comprehensive treatment strategies is of great importance (Phua et al., 2009; Zhang et al., 2020; Liu et al., 2021).

**TABLE 10:** Glossary of terms used in the study.

Term	Definition
Fu Zheng Jie Du Formula (FZJDF)	A traditional Chinese medicine formula composed of eight herbs, used in this study to treat COVID-19 patients
Qi	A vital energy or life force that flows through the body according to traditional Chinese medicine concepts
Yang	A concept in traditional Chinese medicine that represents the active, warming, and drying aspects of the body
Prone ventilation	A mechanical ventilation strategy where patients are positioned face-down to improve oxygenation and respiratory mechanics
Decoction	A method of extraction by boiling herbal materials to dissolve the chemicals of the material in the water
Propensity score matching	A statistical technique used to construct an artificial control group by matching treated and untreated subjects on their propensity scores
Chinese Medicine Score	A scoring system used to quantify the severity of COVID-19 symptoms based on traditional Chinese medicine principles
Herbal medicine	A medical system based on the use of plants or plant extracts to treat illnesses or promote health
Pathogenic toxins	In traditional Chinese medicine, pathogenic toxins refer to harmful substances or influences that can cause disease or exacerbate existing conditions. These toxins are believed to disrupt the balance of Qi, Yin, and Yang in the body
Wind	In traditional Chinese medicine, wind is considered one of the six external pathogens that can invade the body and cause disease. Wind is associated with symptoms such as sudden onset, moving pain, and rapid changes in symptoms
Cold	In traditional Chinese medicine, cold is another external pathogen that can cause disease. Cold is associated with symptoms such as chills, cold limbs, and a preference for warm drinks and environments
Heat	In traditional Chinese medicine, heat is an internal pathogen that can result from external factors or internal imbalances. Heat is associated with symptoms such as fever, thirst, and a preference for cold drinks and environments
Dampness	In traditional Chinese medicine, dampness is an external or internal pathogen that can cause disease. Dampness is associated with symptoms such as a feeling of heaviness, sticky secretions, and a preference for dry environments

The rationale for combining FZJDF with prone ventilation is based on their complementary mechanisms of action. Prone ventilation improves oxygenation by optimizing ventilation-perfusion matching and reducing ventilator-induced lung injury (Li et al., 2020; Li BH. et al., 2021). FZJDF, on the other hand, targets the underlying pathophysiology of severe pneumonia from the perspective of TCM. According to TCM theory, severe pneumonia is caused by the invasion of pathogenic factors, such as wind, cold, heat, and dampness, which lead to deficiency of Yang Qi and the invasion of pathogenic toxins into the lungs. FZJDF contains a combination of herbs that can benefiting Qi and warming Yang, resolving dampness and detoxifying toxins, thereby restoring the balance of Qi and Yang in the lung (Liu et al., 2022).

The improvement in oxygenation with FZJDF may be attributed to its anti-inflammatory and anti-oxidative effects. Previous studies have demonstrated that FZJDF can inhibit the production of pro-inflammatory cytokines, such as TNF-α, IL-1β, and IL-6, and reduce oxidative stress in lung tissue (Butt et al., 2016; Li G. et al., 2021; Liu et al., 2022). These findings are consistent with our observation of a significant reduction in inflammatory markers, including WBC, PCT, Hs-CRP, and IL-6, in the FZJD group compared to the non-FZJD group. The alleviation of inflammation and oxidative stress may contribute to the preservation of alveolar-capillary membrane integrity, reduction of pulmonary edema, and improvement of gas exchange (Ware, 2006; Matthay et al., 2019).

The shortened duration of mechanical ventilation and ICU stay in the FZJD group may be a result of the faster resolution of symptoms and improvement in overall condition. We found that patients in the FZJD group had significantly lower APACHE II score, SOFA score, and Chinese Medicine Score compared to the non-FZJD group, indicating a better response to treatment (Knaus et al., 1985; Vincent et al., 1996). The faster resolution of fever, cough, and pulmonary rales in the FZJD group also suggests that FZJDF may promote the recovery of lung function and reduce the risk of ventilator-associated complications (Cavallazzi et al., 2015; Béduneau et al., 2017). These findings highlight the potential of FZJDF as an adjuvant therapy to enhance the efficacy of conventional treatment and prone ventilation in patients with severe pneumonia (Liu et al., 2004; Guo et al., 2022).

A recent study by Lu et al. (2024) has suggested that FZJDF may exert its therapeutic effects in severe pneumonia, at least in part, by modulating the gut-lung axis. Using a lipopolysaccharide-induced acute lung injury mouse model, they found that FZJDF treatment alleviated intestinal barrier dysfunction, reduced systemic and pulmonary inflammation, and attenuated lung histopathological changes, which were associated with the regulation of key signaling pathways, such as NF-κB and MAPK, in both the gut and the lung. These findings provide new insights into the potential mechanisms of action of FZJDF in severe pneumonia, highlighting the importance of the gut-lung axis in the pathogenesis and treatment of respiratory diseases (Sencio et al., 2021). However, further clinical studies are needed to validate this hypothesis and guide the optimal use of FZJDF in patients with severe pneumonia.

Our study has several limitations. First, it was a single-center retrospective study with a relatively small sample size, which may limit the generalizability of the findings. Second, the use of a self-designed Chinese Medicine Score may not be validated and could be subject to bias. Third, we did not assess the long-term outcomes or quality of life of the patients after discharge. Fourth, the mechanism of action of FZJDF in severe pneumonia remains to be fully elucidated.

In conclusion, our study suggests that FZJDF combined with prone ventilation may be an effective adjuvant therapy for patients with severe pneumonia. It can improve oxygenation, reduce inflammation, and shorten the duration of mechanical ventilation and ICU stay. However, prospective, multicenter, randomized controlled trials are needed to confirm these findings and further evaluate the safety and efficacy of FZJDF in severe pneumonia. The underlying mechanisms of FZJDF should also be investigated in future studies to better guide its clinical application.

## Conclusion

Our study demonstrates that FZJDF combined with prone ventilation is superior to prone ventilation alone in improving oxygenation, reducing inflammation, and shortening the duration of mechanical ventilation and ICU stay in patients with severe pneumonia. These findings suggest that FZJDF is a promising adjuvant therapy for severe pneumonia.

The beneficial effects of FZJDF may be attributed to its unique composition and multifaceted pharmacological actions, which could alleviate the inflammatory storm, regulate immune dysfunction, and inhibit viral replication. This hypothesis is supported by previous studies on the efficacy of traditional Chinese medicine formulas in severe pneumonia.

However, our study has limitations, including the retrospective design, single-center setting, and lack of long-term follow-up. Further high-quality research is needed to validate our findings and guide the evidence-based use of FZJDF in clinical practice. Future studies should include large-scale, prospective, randomized controlled trials, investigate the mechanisms of action, evaluate long-term effects, optimize dosage and administration regimens, and identify subgroups of patients who may benefit most from this intervention.

In conclusion, our study highlights the potential of FZJDF as an adjuvant therapy for severe pneumonia and supports the integration of traditional Chinese medicine and Western medicine in treating critically ill patients. Further research is warranted to validate these findings and guide evidence-based clinical practice.

## Data Availability

The raw data supporting the conclusions of this article will be made available by the authors, without undue reservation.
