# Supplementary material for: Effect of Fu Zheng Jie Du Formula on outcomes in patients with severe pneumonia receiving prone ventilation: a retrospective cohort study
Source: Front Pharmacol. 2024 Jul 24;15:1428817. doi: 10.3389/fphar.2024.1428817 (PMC11303160; doi:10.3389/fphar.2024.1428817)
Supplement: Supplementary file 1 [file Table1.DOCX]

Supplementary Table S1

| Active ingredients targets（462） | Severe pneumonia targets | | Common targets（221） |
| --- | --- | --- | --- |
|  | GeneCard（4476） | OMIM（50） |  |
| ABAT | JAK3 | ABCA4 | PGR |
| ABCC1 | RAG1 | ACTA1 | NR3C2 |
| ABCF2 | ADA | ACTL6B | PTGS1 |
| ABCF3 | DCLRE1C | ADA | PTGS2 |
| ABCG2 | IL2RG | APOE | RELA |
| ACACA | RAG2 | BCL11B | AKT1 |
| ACHE | MECP2 | CACNA1G | BCL2 |
| ACOT11 | CSF3R | CD3D | MAPK3 |
| ACOT12 | ELANE | CLPB | MAPK14 |
| ACP3 | ABCA4 | CR1 | CASP3 |
| ACSF3 | PRKDC | CSF3R | FASN |
| ACVR1 | TNF | DCLRE1C | BAD |
| ADCY2 | SCN1A | ELANE | SOD1 |
| ADH1C | G6PC3 | FCGR2A | TYRP1 |
| ADIPOQ | HAX1 | FHL1 | HMGCR |
| ADRA1A | IL6 | FOCAD | CYP19A1 |
| ADRA1B | WAS | G6PC3 | GSTP1 |
| ADRA1D | SLC17A5 | GFI1 | PPARA |
| ADRA2A | PURA | HAX1 | SREBF1 |
| ADRB1 | IKBKB | IFITM3 | GSR |
| ADRB2 | CRP | IL2RG | ABCC1 |
| ADSL | CARD11 | IL7R | ADIPOQ |
| ADSS2 | VPS45 | JAGN1 | CES1 |
| AHR | ZAP70 | KRT14 | SOAT1 |
| AHRR | LIG4 | KRT5 | CALML5 |
| AHSA1 | IL10 | LAMA3 | SCN5A |
| AKR1B10 | CPT2 | LAMB3 | TOP3A |
| AKR1C1 | LINC02258 | LAMC2 | KCNH2 |
| AKR1C3 | IFNG | LIPT2 | F8 |
| AKT1 | IDS | LRAT | NOS2 |
| ALG5 | CASR | MC3R | ESR2 |
| ALOX5 | JAGN1 | MECP2 | CHEK1 |
| ALOX5AP | CXCL8 | MEGF10 | PRSS1 |
| AMY2A | MEGF10 | NHEJ1 | GSK3B |
| AMZ1 | SKAP1-AS1 | PPARG | BAX |
| AMZ2 | LOC107303343 | PPP1R3A | CASP9 |
| APOBR | IL1B | PTPRC | MMP9 |
| APP | IL7R | RAG1 | TP53 |
| ATP5F1B | ACE | RAG2 | MAPK8IP1 |
| ATP6V1B1 | MTM1 | SCN4A | TIMP1 |
| ATP6V1B2 | LAMA3 | SEC61A1 | CREB1 |
| AZIN1 | CTNNB1 | SLC2A1 | CD163 |
| AZIN2 | TGFB1 | SRP54 | HSP90AB1 |
| BAD | KRT5 | SRP68 | ACHE |
| BAIAP2L1 | LAMC2 | TAF8 | ADRB2 |
| BAX | GFI1 | TNNT1 | DPP9 |
| BCAT2 | TLR4 | UCP3 | CALML3 |
| BCL2 | SFTPC | VPS45 | ESR1 |
| BCL2L11 | ALB | WAS | DRD1 |
| BCL2L12 | LOC102724058 | WNT7A | CHRM3 |
| BIRC5 | CORO1A |  | CHRM1 |
| BMAL1 | IL4 |  | CHRM2 |
| BMP10 | LCK |  | SLC6A4 |
| BMP15 | CD3E |  | OPRM1 |
| BMP3 | ACE2 |  | GABRA1 |
| BMP5 | MUC5B |  | CASP8 |
| BMP8A | SFTPA1 |  | PRKCA |
| BMP8B | PTPRC |  | TGFB1 |
| BRSK1 | APOE |  | PON1 |
| BRSK2 | LINC02605 |  | MAP2 |
| CA2 | CCL2 |  | NSD1 |
| CA3 | IL13 |  | RXRA |
| CACNA2D1 | CFTR |  | CAMKMT |
| CALM2 | HLA-B |  | LTA4H |
| CALML3 | LAMB3 |  | PPARGC1A |
| CALML5 | FGFR3 |  | COLQ |
| CAMKMT | MARS1 |  | CDK2 |
| CAMTA1 | CD40LG |  | CCNA2 |
| CASP1 | IL17A |  | IL1B |
| CASP3 | MBL2 |  | CPB2 |
| CASP7 | FOXP1 |  | TOP2A |
| CASP8 | CSF3 |  | F7 |
| CASP9 | PARN |  | MAOB |
| CAV1 | SERPINA1 |  | VEGFA |
| CCL2 | LOC126863274 |  | CCND1 |
| CCL28 | TERT |  | BCL2L11 |
| CCNA2 | TLR2 |  | FOS |
| CCNB1 | ACTA1 |  | CDKN1A |
| CCND1 | SFTPA2 |  | EIF6 |
| CCND2 | DOCK8 |  | MRC2 |
| CD163 | KRT14 |  | MMP2 |
| CD36 | MPO |  | IL10RB |
| CD40LG | NHEJ1 |  | EGF |
| CD79B | IL2 |  | E2F1 |
| CDC25C | ABCA3 |  | IL6 |
| CDK1 | LACTB |  | NFKBIA |
| CDK2 | GPT |  | POR |
| CDK20 | HMOX1 |  | XDH |
| CDK4 | IL1A |  | RAF1 |
| CDK6 | HLA-DRB1 |  | MMP1 |
| CDKN1A | IL5 |  | STAT1 |
| CDKN1B | IL18 |  | RUNX1T1 |
| CDKN1C | LOC106050102 |  | CDK1 |
| CES1 | TP53 |  | HSPA5 |
| CHEK1 | LTBP4 |  | ERBB2 |
| CHEK2 | SRP54 |  | ACACA |
| CHRM1 | DSP |  | HMOX1 |
| CHRM2 | MYH7 |  | CYP3A4 |
| CHRM3 | CCL5 |  | CYP1A2 |
| CHRM4 | RTEL1 |  | CAV1 |
| CHRM5 | CD4 |  | MYC |
| CHRNA2 | CBS |  | TFPI |
| CHUK | MMP1 |  | GJA1 |
| CLDN4 | TERC |  | CYP1A1 |
| COL1A1 | F2 |  | ICAM1 |
| COL3A1 | SFTPD |  | CCL2 |
| COLQ | CD8A |  | SELE |
| CORO7-PAM16 | STAT3 |  | VCAM1 |
| CPB2 | CSF2 |  | CXCL8 |
| CPSF2 | MIR155 |  | BIRC5 |
| CPSF3 | CXCL10 |  | DUOX2 |
| CREB1 | DHFR |  | NOS3 |
| CRP | PGM3 |  | IFNL2 |
| CTDSP1 | ASXL3 |  | CYP1B1 |
| CTNNB1 | MMP9 |  | PLAT |
| CTRB1 | PNP |  | THBD |
| CTRB2 | INS |  | SERPINE1 |
| CTSD | HLA-A |  | COL1A1 |
| CUTA | IL1RN |  | CXCL11 |
| CXCL10 | HMGB1 |  | ALOX5 |
| CXCL11 | AK2 |  | IL1A |
| CXCL2 | NEU1 |  | MPO |
| CXCL8 | SFTPB |  | NCF1 |
| CYP19A1 | CHAT |  | ABCG2 |
| CYP1A1 | RAC2 |  | NFE2L2 |
| CYP1A2 | CCL11 |  | NQO1 |
| CYP1B1 | CTPS1 |  | PSMD3 |
| CYP3A4 | H19 |  | SLC2A4 |
| CYP3A43 | HBB |  | COL3A1 |
| CYTH3 | LMNA |  | CXCL2 |
| DCAF5 | LEP |  | CHEK2 |
| DCLRE1A | CD3D |  | PPARD |
| DCLRE1B | ICAM1 |  | CRP |
| DCLRE1C | LINC01672 |  | CXCL10 |
| DIO1 | MIR146B |  | CHUK |
| DNAJC15 | IFIH1 |  | SPP1 |
| DNTTIP2 | FCGR2A |  | RUNX2 |
| DOK4 | CLN3 |  | CTSD |
| DOK5 | CCL3 |  | IGF2 |
| DPEP3 | IGF1 |  | CD40LG |
| DPM1 | ATM |  | IRF1 |
| DPP6 | FOXN1 |  | SYNGAP1 |
| DPP8 | NPC1 |  | GSTM1 |
| DPP9 | ALMS1 |  | HSP90AA1 |
| DRD1 | MIR21 |  | PYGM |
| DUOX2 | TCIRG1 |  | F2R |
| E2F1 | CLPB |  | CTNNB1 |
| E2F2 | COL1A1 |  | CASP7 |
| EBF1 | COL7A1 |  | F3 |
| EBF2 | MIR146A |  | MMP10 |
| EBF3 | F8 |  | CACNA2D1 |
| EBF4 | PGR-AS1 |  | APP |
| ECE2 | MFN2 |  | LTBP1 |
| ECEL1 | TLR3 |  | SLC6A2 |
| EEFSEC | HLA-DQB1 |  | IKBKB |
| EGF | SERPINE1 |  | ALOX5AP |
| EGFLAM | MTHFD1 |  | BMAL1 |
| EIF6 | IL7 |  | PPP3CA |
| ELK1 | SCN2A |  | SLPI |
| ENO4 | TTC7A |  | SLC6A3 |
| ENPP4 | BSCL2 |  | PLAU |
| EPHB2 | STAT1 |  | MAOA |
| EPS15 | IL2RA |  | CDKN1B |
| EPS15L1 | TTN |  | MAPK1 |
| EPS8L3 | TLR5 |  | IL10RA |
| ERBB2 | F3 |  | CDK4 |
| ERBB3 | FAM13A |  | MDM2 |
| ESR1 | F5 |  | MCL1 |
| ESR2 | EGFR |  | IL27 |
| ESRRB | DPM2 |  | IL4R |
| F2R | CTLA4 |  | XIAP |
| F2RL1 | ATP11A |  | MET |
| F2RL3 | SCN9A |  | HIF1A |
| F3 | F9 |  | PARP1 |
| F7 | TMX2-CTNND1 |  | ACVR1 |
| F8 | CXCR2 |  | ADSL |
| FASN | RMRP |  | AMY2A |
| FBP2 | G6PD |  | ATP6V1B1 |
| FOS | NFKB1 |  | ATP6V1B2 |
| FOSB | TSC2 |  | CD79B |
| GABRA1 | RPE65 |  | IREB2 |
| GBF1 | FHL1 |  | ITPA |
| GDF11 | CD3G |  | SH2D1A |
| GDF3 | CERNA3 |  | NODAL |
| GDF5 | GABRG2 |  | S100A12 |
| GDF6 | VWF |  | XPNPEP2 |
| GDF7 | MIR223 |  | NME5 |
| GDF9 | PPARG |  | DPM1 |
| GDPD1 | COL1A2 |  | NAE1 |
| GDPD2 | FBN1 |  | UBA2 |
| GDPD4 | BTK |  | NOD1 |
| GFPT1 | MUC1 |  | TNFSF13B |
| GFPT2 | MYOC |  | LEMD3 |
| GIG44 | NPC2 |  | KPNA6 |
| GJA1 | AR |  | BMP10 |
| GMPS | AGT |  | ALG5 |
| GNPDA2 | IFNB1 |  | CPSF3 |
| GOT1 | CFH |  | RTEL1 |
| GRIA2 | MEG3 |  | DCLRE1C |
| GSK3B | COL2A1 |  | DCLRE1B |
| GSR | LDLR |  | PAPOLG |
| GSTM1 | NLRP3 |  | LEMD2 |
| GSTM2 | RTEL1-TNFRSF6B |  | RTEL1-TNFRSF6B |
| GSTP1 | CARD11-AS1 |  | PSMD2 |
| HAS2 | GBA1 |  | MMP7 |
| HDHD2 | ITGAM |  | CASP1 |
| HDHD5 | MASP2 |  | CCND2 |
| HIF1A | BDNF-AS |  | CD36 |
| HIF1AN | DHX30 |  | AHR |
| HIPK3 | PIK3C2A |  | MMP3 |
| HK2 | TNFRSF1A |  | HSPB1 |
| HMGCR | PLEC |  | IFNGR2 |
| HMOX1 | CD14 |  | HTR3A |
| HRCT1 | TREM1 |  | CALM2 |
| HSD3B1 | CDKL5 |  | KDR |
| HSD3B2 | MAPT |  | F2RL1 |
| HSF1 | IFNA1 |  | MAPK10 |
| HSP90AA1 | BDNF |  | GRIA2 |
| HSP90AA2P | FOXP3 |  | OLR1 |
| HSP90AB1 | GATAD2B |  | TNFRSF1B |
| HSPA5 | FKRP |  | LRP5 |
| HSPB1 | PRPH2 |  |  |
| HTR3A | LRBA |  |  |
| ICAM1 | CYBB |  |  |
| IDH3A | VEGFA |  |  |
| IFNGR2 | NR3C2 |  |  |
| IFNL2 | CACNA1G |  |  |
| IFT25 | NOS3 |  |  |
| IGF2 | PTPN11 |  |  |
| IGFBP3 | IGHE |  |  |
| IGFN1 | STK4 |  |  |
| IGHG1 | PRF1 |  |  |
| IGSF22 | MIF |  |  |
| IKBKB | SBDS |  |  |
| IL10RA | DMD |  |  |
| IL10RB | TTR |  |  |
| IL1A | FIG4 |  |  |
| IL1B | MFSD8 |  |  |
| IL27 | PKD1 |  |  |
| IL27RA | MMP8 |  |  |
| IL4R | SCGB1A1 |  |  |
| IL6 | MALT1 |  |  |
| INHBC | RYR1 |  |  |
| INTS11 | IDUA |  |  |
| INTS9 | CD79A |  |  |
| IPPK | SOD1 |  |  |
| IREB2 | LOC126861898 |  |  |
| IRF1 | CR1 |  |  |
| ITPA | IKBKG |  |  |
| JUND | TYK2 |  |  |
| KCNH2 | INSR |  |  |
| KDR | NOS2 |  |  |
| KEL | MALAT1 |  |  |
| KPNA5 | CD19 |  |  |
| KPNA6 | GJB2 |  |  |
| KPNA7 | C3 |  |  |
| L3HYPDH | CXCL1 |  |  |
| LACTB2 | DPP9 |  |  |
| LCMT1 | MED25 |  |  |
| LDLRAD1 | IL2RB |  |  |
| LEFTY2 | TMPRSS2 |  |  |
| LEMD2 | B2M |  |  |
| LEMD3 | LOC110806306 |  |  |
| LHPP | SPAST |  |  |
| LRP5 | PEX1 |  |  |
| LTA4H | MMP2 |  |  |
| LTBP1 | CHD7 |  |  |
| MAOA | SELL |  |  |
| MAOB | CXCR4 |  |  |
| MAP1LC3C | DEFB4A |  |  |
| MAP2 | LOC129994826 |  |  |
| MAPK1 | SMN1 |  |  |
| MAPK10 | ELN |  |  |
| MAPK14 | AKT1 |  |  |
| MAPK15 | CD27 |  |  |
| MAPK3 | STN1 |  |  |
| MAPK8IP1 | LAT |  |  |
| MAPK8IP3 | AVP |  |  |
| MARK1 | PTEN |  |  |
| MARK2 | CD36 |  |  |
| MARK3 | MTHFR |  |  |
| MBLAC2 | SRP19 |  |  |
| MCL1 | SLPI |  |  |
| MDM2 | LOC110806263 |  |  |
| MDM4 | LTA |  |  |
| MET | VCAM1 |  |  |
| MMACHC | CCL22 |  |  |
| MMEL1 | CACNA1A |  |  |
| MMP1 | SH2D1A |  |  |
| MMP10 | CEP290 |  |  |
| MMP2 | SPP1 |  |  |
| MMP3 | PAFAH1B1 |  |  |
| MMP7 | NPPB |  |  |
| MMP9 | FGFR1 |  |  |
| MPO | RNASE3 |  |  |
| MRC2 | HNRNPUL2-BSCL2 |  |  |
| MTERF1 | MMP12 |  |  |
| MTTP | MIR7-3HG |  |  |
| MYC | REN |  |  |
| NAE1 | HSPD1 |  |  |
| NCF1 | NEB |  |  |
| NCOA1 | APP |  |  |
| NCOA2 | GH-LCR |  |  |
| NDC80 | MTOR |  |  |
| NFE2L2 | PROM1 |  |  |
| NFKBIA | CD247 |  |  |
| NME5 | PLG |  |  |
| NME6 | SELENON |  |  |
| NMNAT1 | GLB1 |  |  |
| NMNAT2 | GSN |  |  |
| NMNAT3 | DHPS |  |  |
| NOD1 | ARX |  |  |
| NODAL | IGHMBP2 |  |  |
| NOS2 | POMC |  |  |
| NOS3 | SERPINH1 |  |  |
| NPEPPS | FCGR3B |  |  |
| NPEPPSL1 | LOC107133510 |  |  |
| NQO1 | PIK3CA |  |  |
| NR1I2 | IFNAR1 |  |  |
| NR1I3 | MIR30A |  |  |
| NR3C2 | SPATA7 |  |  |
| NSD1 | ACTL6B |  |  |
| NUDT6 | CALCA |  |  |
| NUF2 | MYD88 |  |  |
| ODF1 | CIITA |  |  |
| OLR1 | BRCA2 |  |  |
| OPRD1 | POMT1 |  |  |
| OPRM1 | ADM |  |  |
| PAM16 | IRAK4 |  |  |
| PAPOLB | SERPINC1 |  |  |
| PAPOLG | NOD2 |  |  |
| PARP1 | LBP |  |  |
| PARP10 | MIR140 |  |  |
| PARP14 | IL15 |  |  |
| PCNA | PRTN3 |  |  |
| PCOLCE | MT-ND1 |  |  |
| PDXP | FLNA |  |  |
| PERM1 | SCN1A-AS1 |  |  |
| PGR | KCNQ2 |  |  |
| PHEX | IL1R1 |  |  |
| PIAS2 | CTSB |  |  |
| PILRA | FN1 |  |  |
| PKIA | MEFV |  |  |
| PLA2G4D | TNFRSF13B |  |  |
| PLAT | DOP1A |  |  |
| PLAU | ARSB |  |  |
| PLB1 | UNC80 |  |  |
| PON1 | GUSB |  |  |
| POR | IL3 |  |  |
| PPARA | EDN1 |  |  |
| PPARD | AGTR1 |  |  |
| PPARGC1A | ADIPOQ |  |  |
| PPARGC1B | TUBA1A |  |  |
| PPP3CA | SMN2 |  |  |
| PPRC1 | CCR5 |  |  |
| PRKCA | LPL |  |  |
| PRKCB | IL22 |  |  |
| PRSS1 | ETFA |  |  |
| PSMD2 | FKTN |  |  |
| PSMD3 | C5AR1 |  |  |
| PSME1 | CTSD |  |  |
| PTGER3 | CYBA |  |  |
| PTGES3L | HFE |  |  |
| PTGS1 | TIMP1 |  |  |
| PTGS2 | MMP3 |  |  |
| PUM1 | PIK3R1 |  |  |
| PUM2 | XIST |  |  |
| PYGM | CHI3L1 |  |  |
| RAD54L2 | STXBP1 |  |  |
| RAF1 | CXCL2 |  |  |
| RASA1 | ITGB2 |  |  |
| RASSF1 | LOC106099062 |  |  |
| RASSF10 | FCHO1 |  |  |
| RCL1 | FCGR3A |  |  |
| RELA | SNCA |  |  |
| REXO2 | FOXG1 |  |  |
| RGS11 | TRPV4 |  |  |
| RGS12 | LOC126861358 |  |  |
| RGS13 | ETFDH |  |  |
| RGS14 | HP |  |  |
| RGS17 | SMPD1 |  |  |
| RGS18 | DSG1 |  |  |
| RGS21 | SLC2A1 |  |  |
| RGS6 | IFNGR1 |  |  |
| RGS8 | CXCL12 |  |  |
| RIMKLB | DPYD |  |  |
| RNH1 | TCF4 |  |  |
| RPH3A | FGFR2 |  |  |
| RTEL1 | TLR7 |  |  |
| RTEL1-TNFRSF6B | GDAP1 |  |  |
| RUNX1T1 | PLA2G6 |  |  |
| RUNX2 | TNNI3 |  |  |
| RXRA | DKC1 |  |  |
| S100A12 | SCN8A |  |  |
| SCN5A | HLA-C |  |  |
| SELE | ALOX5 |  |  |
| SERBP1 | SCN1B |  |  |
| SERPINE1 | STAT5B |  |  |
| SH2D1A | FAS |  |  |
| SIK2 | IFITM3 |  |  |
| SIK3 | IKZF1 |  |  |
| SLC13A1 | ARSA |  |  |
| SLC13A2 | MIR335 |  |  |
| SLC13A3 | RFXANK |  |  |
| SLC13A4 | IL6ST |  |  |
| SLC13A5 | STAT6 |  |  |
| SLC2A4 | GAA |  |  |
| SLC35G3 | NEFL |  |  |
| SLC6A2 | PTGS2 |  |  |
| SLC6A3 | KATNB1 |  |  |
| SLC6A4 | CCL18 |  |  |
| SLPI | MT-CYB |  |  |
| SNX27 | CCL4 |  |  |
| SOAT1 | CXCL5 |  |  |
| SOAT2 | ESR1 |  |  |
| SOD1 | IL4R |  |  |
| SPAG6 | PCDH19 |  |  |
| SPP1 | ERCC6 |  |  |
| SRARP | CARMIL2 |  |  |
| SREBF1 | PMM2 |  |  |
| ST13 | APOA1 |  |  |
| STAT1 | IL9 |  |  |
| STAU1 | TNFRSF1B |  |  |
| STRADA | NEAT1 |  |  |
| SULT1E1 | TBK1 |  |  |
| SYNGAP1 | MAPK1 |  |  |
| TATDN1 | ALPL |  |  |
| TATDN2 | CCL17 |  |  |
| TATDN3 | ZBTB25 |  |  |
| TCF20 | MIR125A |  |  |
| TCF7 | FBLN5 |  |  |
| TFPI | POMGNT1 |  |  |
| TGFB1 | MCFD2 |  |  |
| THBD | MIR34A |  |  |
| THTPA | PDCD1 |  |  |
| TIMP1 | RPGR |  |  |
| TMEM219 | C4A |  |  |
| TNFRSF10D | KMT2D |  |  |
| TNFRSF1B | CFI |  |  |
| TNFSF13B | EFEMP2 |  |  |
| TOP1 | CDKN2A |  |  |
| TOP2A | LTF |  |  |
| TOP3A | MIR210 |  |  |
| TOP3B | HIF1A |  |  |
| TP53 | XIAP |  |  |
| TRADD | SMC1A |  |  |
| TSC22D1 | SOD2-OT1 |  |  |
| TYR | SMAD5-AS1 |  |  |
| TYRP1 | GSTM1 |  |  |
| UBA2 | PROC |  |  |
| UBA6 | VDR |  |  |
| UGT1A1 | LOC121587541 |  |  |
| UGT1A10 | CD209 |  |  |
| USP15 | CXCR3 |  |  |
| VCAM1 | MYO7A |  |  |
| VEGFA | FASLG |  |  |
| WDR36 | IL12RB1 |  |  |
| XDH | NAGLU |  |  |
| XIAP | ASAH1 |  |  |
| XPNPEP2 | ADAMTS13 |  |  |
| YWHAH | SLC6A4 |  |  |
| ZP3 | HLA-DQA1 |  |  |
|  | CASP3 |  |  |
|  | MIR222 |  |  |
|  | GALNS |  |  |
|  | SCN5A |  |  |
|  | MIR29A |  |  |
|  | JUN |  |  |
|  | TBC1D24 |  |  |
|  | L1CAM |  |  |
|  | SLC37A4 |  |  |
|  | MVK |  |  |
|  | EP300 |  |  |
|  | SHH |  |  |
|  | GHR |  |  |
|  | SYK |  |  |
|  | SLC6A3 |  |  |
|  | CREBBP |  |  |
|  | POLG |  |  |
|  | TUG1 |  |  |
|  | AGER |  |  |
|  | APOB |  |  |
|  | TH |  |  |
|  | GSTP1 |  |  |
|  | FOCAD |  |  |
|  | NCF2 |  |  |
|  | JAK1 |  |  |
|  | TMEM67 |  |  |
|  | HRAS |  |  |
|  | USB1 |  |  |
|  | KLHL40 |  |  |
|  | ADRB2 |  |  |
|  | PLAT |  |  |
|  | TULP1 |  |  |
|  | IGF2 |  |  |
|  | THBD |  |  |
|  | BRCA1 |  |  |
|  | LOC126862707 |  |  |
|  | UBE3A |  |  |
|  | SFTA3 |  |  |
|  | DOCK2 |  |  |
|  | GRIN2B |  |  |
|  | DYNC1H1 |  |  |
|  | EPO |  |  |
|  | USH2A |  |  |
|  | COL17A1 |  |  |
|  | RNU4ATAC |  |  |
|  | CP |  |  |
|  | CKMT2 |  |  |
|  | PLP1 |  |  |
|  | DNM2 |  |  |
|  | COMT |  |  |
|  | GABRA1 |  |  |
|  | FGF2 |  |  |
|  | PIK3CD |  |  |
|  | SARDH |  |  |
|  | CYP21A2 |  |  |
|  | MIR221 |  |  |
|  | SRPRA |  |  |
|  | FMR1 |  |  |
|  | CR2 |  |  |
|  | BCL2 |  |  |
|  | RFX5 |  |  |
|  | GATA2 |  |  |
|  | SMAD4 |  |  |
|  | TGFB2 |  |  |
|  | KITLG |  |  |
|  | COL3A1 |  |  |
|  | TPP1 |  |  |
|  | IRF3 |  |  |
|  | MIR148A |  |  |
|  | CKB |  |  |
|  | GNPTAB |  |  |
|  | NF1 |  |  |
|  | POMT2 |  |  |
|  | SELE |  |  |
|  | NKX2-5 |  |  |
|  | NLRC4 |  |  |
|  | ETFB |  |  |
|  | MIR126 |  |  |
|  | SMARCA2 |  |  |
|  | PIK3CG |  |  |
|  | MMP7 |  |  |
|  | STAT2 |  |  |
|  | MIR17 |  |  |
|  | MIR132 |  |  |
|  | ASXL1 |  |  |
|  | SMARCD2 |  |  |
|  | NDE1 |  |  |
|  | ERCC2 |  |  |
|  | MUC5AC |  |  |
|  | TNFSF11 |  |  |
|  | PSEN1 |  |  |
|  | ACTB |  |  |
|  | PDGFRB |  |  |
|  | TF |  |  |
|  | CAT |  |  |
|  | GAS5 |  |  |
|  | SETD2 |  |  |
|  | TBX1 |  |  |
|  | KRAS |  |  |
|  | PRKN |  |  |
|  | USH1C |  |  |
|  | SGSH |  |  |
|  | CXCL9 |  |  |
|  | TRA-TGC7-1 |  |  |
|  | ANK1 |  |  |
|  | PKHD1 |  |  |
|  | C4B |  |  |
|  | GABRB3 |  |  |
|  | IQSEC2 |  |  |
|  | TLR9 |  |  |
|  | FGF8 |  |  |
|  | CD34 |  |  |
|  | GLI2 |  |  |
|  | TGFBR2 |  |  |
|  | BCAP31 |  |  |
|  | MIR30B |  |  |
|  | BRAF |  |  |
|  | LAMB1 |  |  |
|  | PDGFRA |  |  |
|  | IFNAR2 |  |  |
|  | BCL11B |  |  |
|  | NCF1 |  |  |
|  | RIGI |  |  |
|  | EGR2 |  |  |
|  | SERPINB1 |  |  |
|  | GRN |  |  |
|  | IL33 |  |  |
|  | GALC |  |  |
|  | NBN |  |  |
|  | NR3C1 |  |  |
|  | DPP4 |  |  |
|  | LAMA2 |  |  |
|  | VCP |  |  |
|  | LYST |  |  |
|  | MTMR2 |  |  |
|  | MIR409 |  |  |
|  | RHO |  |  |
|  | NRXN1 |  |  |
|  | MT-ATP6 |  |  |
|  | CD274 |  |  |
|  | TSC1 |  |  |
|  | TLR6 |  |  |
|  | SIRT1 |  |  |
|  | RAC1 |  |  |
|  | PAX6 |  |  |
|  | MAPK3 |  |  |
|  | ADSL |  |  |
|  | MIR22 |  |  |
|  | PAH |  |  |
|  | ADNP |  |  |
|  | SH2B1 |  |  |
|  | PWAR1 |  |  |
|  | CARD9 |  |  |
|  | BMP6 |  |  |
|  | GUCY2D |  |  |
|  | ITGB4 |  |  |
|  | CDH1 |  |  |
|  | EMSLR |  |  |
|  | CKM |  |  |
|  | SEC61A1 |  |  |
|  | ABCC8 |  |  |
|  | LCN2 |  |  |
|  | NTRK2 |  |  |
|  | ABCA1 |  |  |
|  | ACTC1 |  |  |
|  | PEX5 |  |  |
|  | CYP2C9 |  |  |
|  | IRF7 |  |  |
|  | COL4A5 |  |  |
|  | CD163 |  |  |
|  | CDC42 |  |  |
|  | COL6A1 |  |  |
|  | AP4E1 |  |  |
|  | SH3TC2 |  |  |
|  | LOC130068781 |  |  |
|  | SRC |  |  |
|  | CSF1 |  |  |
|  | RDH12 |  |  |
|  | IL12B |  |  |
|  | EGF |  |  |
|  | LOC129930930 |  |  |
|  | SPARC |  |  |
|  | PMP22 |  |  |
|  | COL4A1 |  |  |
|  | WDR45 |  |  |
|  | NKX2-1 |  |  |
|  | IL21 |  |  |
|  | OFD1 |  |  |
|  | CLDN19 |  |  |
|  | CD40 |  |  |
|  | VTRNA1-1 |  |  |
|  | TOLLIP |  |  |
|  | TNNT2 |  |  |
|  | RFXAP |  |  |
|  | NFE2L2 |  |  |
|  | XRCC4 |  |  |
|  | CKMT1B |  |  |
|  | NFKBIA |  |  |
|  | WNT1 |  |  |
|  | HLA-G |  |  |
|  | TOR1A |  |  |
|  | HBG2 |  |  |
|  | ARID1B |  |  |
|  | ANGPT2 |  |  |
|  | HGF |  |  |
|  | CCR4 |  |  |
|  | GCH1 |  |  |
|  | ATRX |  |  |
|  | CCDC40 |  |  |
|  | GRIN2A |  |  |
|  | ARID1A |  |  |
|  | FURIN |  |  |
|  | GAPDH |  |  |
|  | DNM1 |  |  |
|  | HBA1 |  |  |
|  | PRPS1 |  |  |
|  | PEX2 |  |  |
|  | C11orf65 |  |  |
|  | NPPA |  |  |
|  | CYP3A4 |  |  |
|  | KCNJ6 |  |  |
|  | PPP3CA |  |  |
|  | MIR127 |  |  |
|  | TNFRSF11B |  |  |
|  | KCNJ11 |  |  |
|  | MIRLET7B |  |  |
|  | CDH23 |  |  |
|  | RPGRIP1 |  |  |
|  | AIPL1 |  |  |
|  | FLAD1 |  |  |
|  | CASK |  |  |
|  | NTRK1 |  |  |
|  | PTCH1 |  |  |
|  | LTBP1 |  |  |
|  | GLA |  |  |
|  | NDRG1 |  |  |
|  | CHD8 |  |  |
|  | MC4R |  |  |
|  | AP4M1 |  |  |
|  | ENG |  |  |
|  | FLT1 |  |  |
|  | NOS1 |  |  |
|  | ABCB1 |  |  |
|  | EXTL3 |  |  |
|  | DNMT3A |  |  |
|  | DAG1 |  |  |
|  | GATA1 |  |  |
|  | TNFSF13B |  |  |
|  | PRKCD |  |  |
|  | KNG1 |  |  |
|  | TNFRSF11A |  |  |
|  | JAK2 |  |  |
|  | PVT1 |  |  |
|  | HBA2 |  |  |
|  | LARGE1 |  |  |
|  | MIR214 |  |  |
|  | STING1 |  |  |
|  | KCNQ1 |  |  |
|  | MIR199B |  |  |
|  | IL21R |  |  |
|  | BCS1L |  |  |
|  | MIR379 |  |  |
|  | GLI3 |  |  |
|  | LRRC56 |  |  |
|  | TGFBR1 |  |  |
|  | SGCG |  |  |
|  | MIR143 |  |  |
|  | SETBP1 |  |  |
|  | COL6A2 |  |  |
|  | CACNA1C |  |  |
|  | SMARCB1 |  |  |
|  | PPT1 |  |  |
|  | LRP5 |  |  |
|  | ANK2 |  |  |
|  | SPTLC1 |  |  |
|  | KRT18 |  |  |
|  | GNAS |  |  |
|  | PDGFB |  |  |
|  | NAA10 |  |  |
|  | ADA2 |  |  |
|  | BSG |  |  |
|  | PEPD |  |  |
|  | ITGB3 |  |  |
|  | LOC126805948 |  |  |
|  | F7 |  |  |
|  | MIR381 |  |  |
|  | MAOA |  |  |
|  | CAV1 |  |  |
|  | RETN |  |  |
|  | GJB1 |  |  |
|  | TUBB3 |  |  |
|  | IL18R1 |  |  |
|  | PRX |  |  |
|  | PTX3 |  |  |
|  | MIR23A |  |  |
|  | NFKB2 |  |  |
|  | ACADVL |  |  |
|  | MAGT1 |  |  |
|  | DHCR7 |  |  |
|  | ERBB2 |  |  |
|  | ITGA4 |  |  |
|  | GFAP |  |  |
|  | GRIN1 |  |  |
|  | SBF2 |  |  |
|  | CREB1 |  |  |
|  | MT-CO1 |  |  |
|  | CD44 |  |  |
|  | IL11 |  |  |
|  | SELP |  |  |
|  | F10 |  |  |
|  | SLC26A4 |  |  |
|  | COL11A2 |  |  |
|  | WDR19 |  |  |
|  | NGF |  |  |
|  | MIR342 |  |  |
|  | PEX12 |  |  |
|  | PLOD1 |  |  |
|  | KRT10 |  |  |
|  | SMARCA4 |  |  |
|  | IFNA2 |  |  |
|  | DCN |  |  |
|  | MIR486-1 |  |  |
|  | IL6R |  |  |
|  | LGALS3 |  |  |
|  | CYP2R1 |  |  |
|  | PON1 |  |  |
|  | CAMP |  |  |
|  | CTSL |  |  |
|  | ISG15 |  |  |
|  | SURF1 |  |  |
|  | OTC |  |  |
|  | GHRL |  |  |
|  | NCF4 |  |  |
|  | ST20 |  |  |
|  | MT-TL1 |  |  |
|  | RELN |  |  |
|  | COL5A1 |  |  |
|  | SOD2 |  |  |
|  | LEPR |  |  |
|  | DNM1L |  |  |
|  | NIPBL |  |  |
|  | POSTN |  |  |
|  | ATP1A2 |  |  |
|  | SCO2 |  |  |
|  | RELA |  |  |
|  | PSAP |  |  |
|  | MIR320A |  |  |
|  | EPM2A |  |  |
|  | ATP1A3 |  |  |
|  | SDHA |  |  |
|  | CHD2 |  |  |
|  | PLAUR |  |  |
|  | PEX19 |  |  |
|  | MAPK8 |  |  |
|  | ABCB11 |  |  |
|  | DYNC2H1 |  |  |
|  | SHANK3 |  |  |
|  | FKBP10 |  |  |
|  | MIR199A1 |  |  |
|  | DNAJC21 |  |  |
|  | SREBF1 |  |  |
|  | PDHA1 |  |  |
|  | PRNP |  |  |
|  | IDH1 |  |  |
|  | NSMCE3 |  |  |
|  | PLA2G7 |  |  |
|  | TRIM21 |  |  |
|  | THPO |  |  |
|  | CST3 |  |  |
|  | LOX |  |  |
|  | HAMP |  |  |
|  | CLCN7 |  |  |
|  | SLC35C1 |  |  |
|  | ATP7B |  |  |
|  | C2 |  |  |
|  | AP3B1 |  |  |
|  | DCX |  |  |
|  | IL1RAPL2 |  |  |
|  | MAN2B1 |  |  |
|  | IGF1R |  |  |
|  | CLEC7A |  |  |
|  | WNT5A |  |  |
|  | MCOLN1 |  |  |
|  | SERPINF1 |  |  |
|  | SLC26A2 |  |  |
|  | TAP2 |  |  |
|  | NSD1 |  |  |
|  | MBTPS2 |  |  |
|  | MAPK14 |  |  |
|  | CFP |  |  |
|  | SYNGAP1 |  |  |
|  | SGCA |  |  |
|  | TBX5 |  |  |
|  | GJC2 |  |  |
|  | HNF1A |  |  |
|  | SP110 |  |  |
|  | SBF1 |  |  |
|  | AARS1 |  |  |
|  | HLA-DPB1 |  |  |
|  | TAP1 |  |  |
|  | CLDN16 |  |  |
|  | S100B |  |  |
|  | CCND1 |  |  |
|  | AHSG |  |  |
|  | MYBPC3 |  |  |
|  | MIR148B |  |  |
|  | EPHX1 |  |  |
|  | PPARA |  |  |
|  | NOTCH3 |  |  |
|  | RHOA |  |  |
|  | IGHM |  |  |
|  | POLA1 |  |  |
|  | TPO |  |  |
|  | CDKN1A |  |  |
|  | SAMD9 |  |  |
|  | HSPG2 |  |  |
|  | HADHA |  |  |
|  | TPM3 |  |  |
|  | TLR1 |  |  |
|  | CYLD |  |  |
|  | VPS13B |  |  |
|  | BMP1 |  |  |
|  | PEX11B |  |  |
|  | KCNT1 |  |  |
|  | HSPB1 |  |  |
|  | RBP4 |  |  |
|  | MIR142 |  |  |
|  | LOC106627981 |  |  |
|  | DRD2 |  |  |
|  | IFNGR2 |  |  |
|  | KAT6A |  |  |
|  | MIR34C |  |  |
|  | RET |  |  |
|  | COL11A1 |  |  |
|  | FKBP5 |  |  |
|  | GATA6 |  |  |
|  | IQCB1 |  |  |
|  | CD28 |  |  |
|  | BCL11A |  |  |
|  | ICOS |  |  |
|  | SOX10 |  |  |
|  | MIR4260 |  |  |
|  | ADAR |  |  |
|  | CHIT1 |  |  |
|  | PEX26 |  |  |
|  | ALG13 |  |  |
|  | CDH2 |  |  |
|  | MT-CO3 |  |  |
|  | TBR1 |  |  |
|  | PEX13 |  |  |
|  | DYRK1A |  |  |
|  | TCF3 |  |  |
|  | CNTNAP1 |  |  |
|  | ITGAL |  |  |
|  | HGSNAT |  |  |
|  | CDKN2B-AS1 |  |  |
|  | CXCR1 |  |  |
|  | HSPA5 |  |  |
|  | GDNF |  |  |
|  | AIRE |  |  |
|  | TNFRSF13C |  |  |
|  | F11 |  |  |
|  | MT-ND2 |  |  |
|  | LOC129930068 |  |  |
|  | LIPA |  |  |
|  | ZIC2 |  |  |
|  | CYP46A1 |  |  |
|  | COL6A3 |  |  |
|  | BMP4 |  |  |
|  | UCP3 |  |  |
|  | TYMS |  |  |
|  | GJB6 |  |  |
|  | MT-ND4 |  |  |
|  | HTT |  |  |
|  | CLTCL1 |  |  |
|  | B3GALT6 |  |  |
|  | PGF |  |  |
|  | PPIB |  |  |
|  | KIT |  |  |
|  | PECAM1 |  |  |
|  | TINF2 |  |  |
|  | CLN5 |  |  |
|  | CYP2D6 |  |  |
|  | BCHE |  |  |
|  | UNC119 |  |  |
|  | PANK2 |  |  |
|  | LTBP3 |  |  |
|  | SLC26A1 |  |  |
|  | WFS1 |  |  |
|  | OSTM1 |  |  |
|  | LOC126861258 |  |  |
|  | PEX16 |  |  |
|  | ALG12 |  |  |
|  | GNE |  |  |
|  | FANCA |  |  |
|  | PIGA |  |  |
|  | FMO3 |  |  |
|  | FTO |  |  |
|  | CD79B |  |  |
|  | CAPN3 |  |  |
|  | SMAD3 |  |  |
|  | FBN2 |  |  |
|  | KIF1A |  |  |
|  | S100A9 |  |  |
|  | ERCC5 |  |  |
|  | BAG3 |  |  |
|  | ATP7A |  |  |
|  | TIRAP |  |  |
|  | SRP68 |  |  |
|  | BBS2 |  |  |
|  | DES |  |  |
|  | CYP2C19 |  |  |
|  | CYBC1 |  |  |
|  | CHUK |  |  |
|  | MIR382 |  |  |
|  | MT-CO2 |  |  |
|  | CCR7 |  |  |
|  | FLT3 |  |  |
|  | MIR130A |  |  |
|  | PSMC3 |  |  |
|  | ERCC1 |  |  |
|  | MIR98 |  |  |
|  | CD70 |  |  |
|  | KAT6B |  |  |
|  | B4GAT1 |  |  |
|  | RUNX1 |  |  |
|  | CD69 |  |  |
|  | IFT140 |  |  |
|  | CHKA |  |  |
|  | MYLK |  |  |
|  | BBS1 |  |  |
|  | ITGB1 |  |  |
|  | FCGR2B |  |  |
|  | OXT |  |  |
|  | EEF1A2 |  |  |
|  | HERC2 |  |  |
|  | KARS1 |  |  |
|  | TSEN54 |  |  |
|  | RAB3GAP1 |  |  |
|  | CYP1A1 |  |  |
|  | SH2D3A |  |  |
|  | MIR125B1 |  |  |
|  | TFRC |  |  |
|  | GAD1 |  |  |
|  | FOXP2 |  |  |
|  | DYSF |  |  |
|  | DNAH8 |  |  |
|  | MIR93 |  |  |
|  | NTF3 |  |  |
|  | CASP1 |  |  |
|  | EYS |  |  |
|  | NHLRC1 |  |  |
|  | TTC21B |  |  |
|  | PRKAR1A |  |  |
|  | ORAI1 |  |  |
|  | XDH |  |  |
|  | MET |  |  |
|  | GATA4 |  |  |
|  | CCN2 |  |  |
|  | HTR1A |  |  |
|  | MBD5 |  |  |
|  | MRC1 |  |  |
|  | TTN-AS1 |  |  |
|  | EMD |  |  |
|  | MT-ND5 |  |  |
|  | VPS33A |  |  |
|  | SDHD |  |  |
|  | MB |  |  |
|  | STXBP2 |  |  |
|  | TRAF3 |  |  |
|  | HTR2A |  |  |
|  | AGTR2 |  |  |
|  | LINC01554 |  |  |
|  | SNRPN |  |  |
|  | APOH |  |  |
|  | DNAH11 |  |  |
|  | PLAU |  |  |
|  | SIX3 |  |  |
|  | SNORD15A |  |  |
|  | TET2 |  |  |
|  | BLM |  |  |
|  | MIR150 |  |  |
|  | GSTT1 |  |  |
|  | ABCB4 |  |  |
|  | SERPINF2 |  |  |
|  | APC |  |  |
|  | PC |  |  |
|  | PTPN22 |  |  |
|  | MYC |  |  |
|  | GZMB |  |  |
|  | NAT2 |  |  |
|  | HNF1B |  |  |
|  | LOC106780800 |  |  |
|  | PTH1R |  |  |
|  | PHOX2B |  |  |
|  | MMP13 |  |  |
|  | CHEK2 |  |  |
|  | MAPK10 |  |  |
|  | BMPR2 |  |  |
|  | VIM |  |  |
|  | KRT1 |  |  |
|  | EDNRA |  |  |
|  | ADAM17 |  |  |
|  | ZFYVE26 |  |  |
|  | PYCR1 |  |  |
|  | TGIF1 |  |  |
|  | IRS1 |  |  |
|  | FGB |  |  |
|  | CFAP410 |  |  |
|  | CTSG |  |  |
|  | SNAP25 |  |  |
|  | SMAD2 |  |  |
|  | AICDA |  |  |
|  | DNMT3B |  |  |
|  | TOP2A |  |  |
|  | ERCC4 |  |  |
|  | HTRA1 |  |  |
|  | S100A1 |  |  |
|  | EHMT1 |  |  |
|  | CFB |  |  |
|  | PRL |  |  |
|  | TSLP |  |  |
|  | RECQL4 |  |  |
|  | RIPK1 |  |  |
|  | CRH |  |  |
|  | COL5A2 |  |  |
|  | ACADM |  |  |
|  | TNXB |  |  |
|  | UCA1 |  |  |
|  | ESR2 |  |  |
|  | GYPA |  |  |
|  | PSMA7 |  |  |
|  | CTSA |  |  |
|  | SPG11 |  |  |
|  | RRM2B |  |  |
|  | HPRT1 |  |  |
|  | WDR62 |  |  |
|  | TRPV1 |  |  |
|  | ZMPSTE24 |  |  |
|  | CCR2 |  |  |
|  | MIR99B |  |  |
|  | CENPJ |  |  |
|  | SCNN1B |  |  |
|  | CCR1 |  |  |
|  | HNF4A |  |  |
|  | GCDH |  |  |
|  | KDM5C |  |  |
|  | MIR30D |  |  |
|  | SUOX |  |  |
|  | CD81 |  |  |
|  | PARP1 |  |  |
|  | TRIP11 |  |  |
|  | KCNH2 |  |  |
|  | FGG |  |  |
|  | HOTAIR |  |  |
|  | EXOSC3 |  |  |
|  | ATP8B1 |  |  |
|  | SLX1A-SULT1A3 |  |  |
|  | PRRT2 |  |  |
|  | VTN |  |  |
|  | ACKR1 |  |  |
|  | MT-ND3 |  |  |
|  | TPM2 |  |  |
|  | C9orf72 |  |  |
|  | TECPR2 |  |  |
|  | TARDBP |  |  |
|  | KDM6A |  |  |
|  | SQSTM1 |  |  |
|  | MDM2 |  |  |
|  | YARS1 |  |  |
|  | PPARGC1A |  |  |
|  | LAMP2 |  |  |
|  | GNAO1 |  |  |
|  | CLN8 |  |  |
|  | MAF |  |  |
|  | SMARCE1 |  |  |
|  | PTAFR |  |  |
|  | TFG |  |  |
|  | ABL1 |  |  |
|  | ELP1 |  |  |
|  | SUMF1 |  |  |
|  | CLN6 |  |  |
|  | LONP1 |  |  |
|  | UNC13D |  |  |
|  | PIGN |  |  |
|  | MIR29C |  |  |
|  | MIR197 |  |  |
|  | TUBB |  |  |
|  | SPINK5 |  |  |
|  | NIN |  |  |
|  | SP1 |  |  |
|  | MT-TS1 |  |  |
|  | CGAS |  |  |
|  | JAG1 |  |  |
|  | TLX1NB |  |  |
|  | TAF1 |  |  |
|  | AHI1 |  |  |
|  | TK2 |  |  |
|  | ANXA5 |  |  |
|  | KMT2A |  |  |
|  | ABCD1 |  |  |
|  | CD86 |  |  |
|  | SPTAN1 |  |  |
|  | PNPLA6 |  |  |
|  | FGF13 |  |  |
|  | PKD2 |  |  |
|  | MIAT |  |  |
|  | TP63 |  |  |
|  | PRKG1 |  |  |
|  | TRAF6 |  |  |
|  | OCRL |  |  |
|  | OPRM1 |  |  |
|  | ENO1 |  |  |
|  | MIR495 |  |  |
|  | BLNK |  |  |
|  | FGF23 |  |  |
|  | CYB5R3 |  |  |
|  | SST |  |  |
|  | SLC6A8 |  |  |
|  | IDO1 |  |  |
|  | SCNN1A |  |  |
|  | PUF60 |  |  |
|  | TAC1 |  |  |
|  | EPRS1 |  |  |
|  | CYP51A1 |  |  |
|  | MECOM |  |  |
|  | MIR154 |  |  |
|  | COL9A2 |  |  |
|  | SLC1A2 |  |  |
|  | IRF8 |  |  |
|  | LBR |  |  |
|  | AGRN |  |  |
|  | NPHP4 |  |  |
|  | MMP14 |  |  |
|  | BGLAP |  |  |
|  | CFTR-AS1 |  |  |
|  | NPHS1 |  |  |
|  | CYP19A1 |  |  |
|  | CCND2 |  |  |
|  | MIR27A |  |  |
|  | C1QBP |  |  |
|  | LOC126861245 |  |  |
|  | TIMM50 |  |  |
|  | WT1 |  |  |
|  | IRF1 |  |  |
|  | KCNA2 |  |  |
|  | SIGMAR1 |  |  |
|  | HMGCR |  |  |
|  | SNAP29 |  |  |
|  | PCDH15 |  |  |
|  | CDK5RAP2 |  |  |
|  | BIN1 |  |  |
|  | MIR106B |  |  |
|  | GRIA1 |  |  |
|  | COPB1 |  |  |
|  | RPS19 |  |  |
|  | CASP8 |  |  |
|  | ADGRG1 |  |  |
|  | SMC3 |  |  |
|  | CACNA1F |  |  |
|  | SKIC2 |  |  |
|  | PLA2G2A |  |  |
|  | TBX4 |  |  |
|  | CSF1R |  |  |
|  | MIR15A |  |  |
|  | DEPDC5 |  |  |
|  | MKS1 |  |  |
|  | PGR |  |  |
|  | TG |  |  |
|  | ACHE |  |  |
|  | MIR369 |  |  |
|  | PINK1 |  |  |
|  | IGLL1 |  |  |
|  | MYL2 |  |  |
|  | MAP2K1 |  |  |
|  | VAMP1 |  |  |
|  | RAD21 |  |  |
|  | ABCA12 |  |  |
|  | MIRLET7C |  |  |
|  | FOS |  |  |
|  | SETX |  |  |
|  | ACTA2 |  |  |
|  | FUCA1 |  |  |
|  | BIRC5 |  |  |
|  | PSMB8 |  |  |
|  | TBX6 |  |  |
|  | STIL |  |  |
|  | NRG1 |  |  |
|  | HCRT |  |  |
|  | POLR3A |  |  |
|  | GCG |  |  |
|  | PCSK9 |  |  |
|  | TNFSF12 |  |  |
|  | LRRK2 |  |  |
|  | IRF5 |  |  |
|  | SATB2 |  |  |
|  | DDX3X |  |  |
|  | KIAA0586 |  |  |
|  | FGA |  |  |
|  | BMPR1B |  |  |
|  | GSK3B |  |  |
|  | CTSC |  |  |
|  | KPTN |  |  |
|  | ABCC1 |  |  |
|  | EIF2AK2 |  |  |
|  | CHM |  |  |
|  | STAT4 |  |  |
|  | VHL |  |  |
|  | TGM1 |  |  |
|  | TXN |  |  |
|  | GGT1 |  |  |
|  | MSH2 |  |  |
|  | PAX1 |  |  |
|  | C1R |  |  |
|  | ATP6V0A2 |  |  |
|  | FHIT |  |  |
|  | MIR452 |  |  |
|  | WRN |  |  |
|  | NPY |  |  |
|  | DYNC2LI1 |  |  |
|  | ITPA |  |  |
|  | FRMD4A |  |  |
|  | BICD2 |  |  |
|  | NOTCH1 |  |  |
|  | SCGB3A2 |  |  |
|  | ATN1 |  |  |
|  | DCTN1 |  |  |
|  | GRIK2 |  |  |
|  | NRAS |  |  |
|  | IRF6 |  |  |
|  | TGFB3 |  |  |
|  | TGFBI |  |  |
|  | IL23A |  |  |
|  | TRPS1 |  |  |
|  | RAF1 |  |  |
|  | ALG14 |  |  |
|  | RPSA |  |  |
|  | PIGO |  |  |
|  | GBE1 |  |  |
|  | DYNC2I2 |  |  |
|  | NSD2 |  |  |
|  | IL16 |  |  |
|  | INF2 |  |  |
|  | TCOF1 |  |  |
|  | AUTS2 |  |  |
|  | FUS |  |  |
|  | MME |  |  |
|  | FOXH1 |  |  |
|  | COQ2 |  |  |
|  | IL10RA |  |  |
|  | MIR338 |  |  |
|  | OCA2 |  |  |
|  | IRF9 |  |  |
|  | PAX2 |  |  |
|  | BCL10 |  |  |
|  | MTR |  |  |
|  | CDKN1B |  |  |
|  | GRP |  |  |
|  | ARG1 |  |  |
|  | ERF |  |  |
|  | FGF10 |  |  |
|  | SOCS1 |  |  |
|  | CD55 |  |  |
|  | DBH |  |  |
|  | PI4KA |  |  |
|  | FGD4 |  |  |
|  | SLC25A4 |  |  |
|  | LIPC |  |  |
|  | RB1 |  |  |
|  | FA2H |  |  |
|  | CYP2E1 |  |  |
|  | NODAL |  |  |
|  | DUOX2 |  |  |
|  | NHS |  |  |
|  | GYPC |  |  |
|  | UCHL1 |  |  |
|  | ATXN3 |  |  |
|  | UGT1A9 |  |  |
|  | PROCR |  |  |
|  | C1S |  |  |
|  | CUX2 |  |  |
|  | S100A8 |  |  |
|  | BCR |  |  |
|  | LTBP2 |  |  |
|  | MBP |  |  |
|  | TUBB2B |  |  |
|  | CCR3 |  |  |
|  | XRCC6 |  |  |
|  | KATNAL2 |  |  |
|  | LOC109610631 |  |  |
|  | STRA6 |  |  |
|  | KCNQ1OT1 |  |  |
|  | HSPA4 |  |  |
|  | MIR145 |  |  |
|  | TWNK |  |  |
|  | BAX |  |  |
|  | ALDH18A1 |  |  |
|  | THBS1 |  |  |
|  | CRPPA |  |  |
|  | CHRNA7 |  |  |
|  | HDAC8 |  |  |
|  | GJA1 |  |  |
|  | TIMP3 |  |  |
|  | SLC25A22 |  |  |
|  | CARD14 |  |  |
|  | SNHG16 |  |  |
|  | HEXA |  |  |
|  | TBP |  |  |
|  | SCN11A |  |  |
|  | DNAJC5 |  |  |
|  | MS4A1 |  |  |
|  | ASPA |  |  |
|  | OLR1 |  |  |
|  | DEFA1 |  |  |
|  | TNFRSF8 |  |  |
|  | ALG1 |  |  |
|  | LIF |  |  |
|  | ENO2 |  |  |
|  | ATRIP |  |  |
|  | HAVCR2 |  |  |
|  | KMT5B |  |  |
|  | OTOF |  |  |
|  | SUCLG1 |  |  |
|  | NAGA |  |  |
|  | PNPO |  |  |
|  | SOX11 |  |  |
|  | POGZ |  |  |
|  | S100A12 |  |  |
|  | BMP2 |  |  |
|  | PLCG2 |  |  |
|  | TPR |  |  |
|  | NAIP |  |  |
|  | ENPP2 |  |  |
|  | NACC1 |  |  |
|  | RBCK1 |  |  |
|  | CAMK2G |  |  |
|  | RNASEH2C |  |  |
|  | KCNQ3 |  |  |
|  | PLAA |  |  |
|  | DMPK |  |  |
|  | CTNS |  |  |
|  | INPP5D |  |  |
|  | ATXN2 |  |  |
|  | ERCC3 |  |  |
|  | CYP1B1 |  |  |
|  | ST3GAL3 |  |  |
|  | FLVCR1 |  |  |
|  | CSNK2B |  |  |
|  | LOC111674472 |  |  |
|  | MAP3K7 |  |  |
|  | ACAD9 |  |  |
|  | MIR423 |  |  |
|  | IRS2 |  |  |
|  | MID1 |  |  |
|  | RD3 |  |  |
|  | PLD3 |  |  |
|  | DRD4 |  |  |
|  | FGF7 |  |  |
|  | FLNC |  |  |
|  | KLF4 |  |  |
|  | NAMPT |  |  |
|  | LOC130064475 |  |  |
|  | CSTB |  |  |
|  | TICAM1 |  |  |
|  | SPG7 |  |  |
|  | SCN3A |  |  |
|  | MERTK |  |  |
|  | CD80 |  |  |
|  | XRCC5 |  |  |
|  | ESCO2 |  |  |
|  | VCL |  |  |
|  | BAP1 |  |  |
|  | DNASE1 |  |  |
|  | ANTXR2 |  |  |
|  | KIF5A |  |  |
|  | CCK |  |  |
|  | KMT2C |  |  |
|  | TBC1D23 |  |  |
|  | MYOT |  |  |
|  | CD46 |  |  |
|  | CSNK2A1 |  |  |
|  | PCNT |  |  |
|  | MAGEL2 |  |  |
|  | IBA57 |  |  |
|  | SOCS3 |  |  |
|  | MICA |  |  |
|  | TUBB4B |  |  |
|  | ROGDI |  |  |
|  | RNF168 |  |  |
|  | MIR144 |  |  |
|  | SERPINA6 |  |  |
|  | CACNA1D |  |  |
|  | EXOSC10 |  |  |
|  | L2HGDH |  |  |
|  | ATR |  |  |
|  | PPIG |  |  |
|  | AEBP1 |  |  |
|  | EPX |  |  |
|  | STIM1 |  |  |
|  | PIGV |  |  |
|  | VRK1 |  |  |
|  | MT-RNR1 |  |  |
|  | APOC3 |  |  |
|  | IL5RA |  |  |
|  | KMT2B |  |  |
|  | TNFAIP3 |  |  |
|  | ATL1 |  |  |
|  | EEF1A1 |  |  |
|  | CYCS |  |  |
|  | EMG1 |  |  |
|  | CLU |  |  |
|  | PCAT1 |  |  |
|  | MIRLET7E |  |  |
|  | TCF7L2 |  |  |
|  | FBXL4 |  |  |
|  | DST |  |  |
|  | SLC25A1 |  |  |
|  | MIR29B1 |  |  |
|  | PQBP1 |  |  |
|  | GNS |  |  |
|  | RILPL1 |  |  |
|  | IRAK1 |  |  |
|  | PAX8 |  |  |
|  | RAD51 |  |  |
|  | ECHS1 |  |  |
|  | IL12A |  |  |
|  | KDR |  |  |
|  | CDK4 |  |  |
|  | CDON |  |  |
|  | MAMLD1 |  |  |
|  | CHRNB2 |  |  |
|  | PXDN |  |  |
|  | MPL |  |  |
|  | SLC22A5 |  |  |
|  | SPTLC2 |  |  |
|  | PYY |  |  |
|  | RANBP2 |  |  |
|  | MEN1 |  |  |
|  | CYP2B6 |  |  |
|  | RXYLT1 |  |  |
|  | SLC52A2 |  |  |
|  | GLDC |  |  |
|  | CLEC4M |  |  |
|  | MIR193A |  |  |
|  | RUNX2 |  |  |
|  | CC2D2A |  |  |
|  | MYH6 |  |  |
|  | BBS10 |  |  |
|  | B3GALNT2 |  |  |
|  | DEAF1 |  |  |
|  | POMGNT2 |  |  |
|  | FYN |  |  |
|  | SUCLA2 |  |  |
|  | ERVW-1 |  |  |
|  | MIR331 |  |  |
|  | GARS1 |  |  |
|  | TPM1 |  |  |
|  | RPS6KA3 |  |  |
|  | PML |  |  |
|  | WDR35 |  |  |
|  | LAMP1 |  |  |
|  | LMNB1 |  |  |
|  | NEFH |  |  |
|  | GPI |  |  |
|  | BPI |  |  |
|  | LINC-ROR |  |  |
|  | CACNA1B |  |  |
|  | OAS1 |  |  |
|  | IL17RA |  |  |
|  | LOC102723407 |  |  |
|  | DGUOK |  |  |
|  | MR1 |  |  |
|  | ADAMTSL2 |  |  |
|  | DPF2 |  |  |
|  | HDAC9 |  |  |
|  | CACNA1S |  |  |
|  | PADI4 |  |  |
|  | NFIX |  |  |
|  | ANGPT1 |  |  |
|  | MED13L |  |  |
|  | NHP2 |  |  |
|  | IRAK3 |  |  |
|  | NF2 |  |  |
|  | TRAPPC9 |  |  |
|  | MIRLET7D |  |  |
|  | MT-TK |  |  |
|  | NDUFS4 |  |  |
|  | RBBP8 |  |  |
|  | ATP6V1A |  |  |
|  | ITGA7 |  |  |
|  | MAG |  |  |
|  | RPL35A |  |  |
|  | LPA |  |  |
|  | EDNRB |  |  |
|  | FANCG |  |  |
|  | ZEB2 |  |  |
|  | RAB5A |  |  |
|  | ADORA2A |  |  |
|  | LAMTOR2 |  |  |
|  | EFL1 |  |  |
|  | ALDOA |  |  |
|  | NOP10 |  |  |
|  | NOG |  |  |
|  | DKK1 |  |  |
|  | MDH2 |  |  |
|  | STX3 |  |  |
|  | KLF1 |  |  |
|  | DNAH5 |  |  |
|  | TREX1 |  |  |
|  | MIR299 |  |  |
|  | WDR1 |  |  |
|  | GAD2 |  |  |
|  | SOX9 |  |  |
|  | S100A4 |  |  |
|  | ACADS |  |  |
|  | GMPPB |  |  |
|  | ARPC1B |  |  |
|  | ALDH7A1 |  |  |
|  | BRAT1 |  |  |
|  | SCNN1G |  |  |
|  | RPL5 |  |  |
|  | PTH |  |  |
|  | HMGCL |  |  |
|  | CNR1 |  |  |
|  | CPS1 |  |  |
|  | TRD-GTC9-1 |  |  |
|  | FCGR1A |  |  |
|  | NEXMIF |  |  |
|  | WIPF1 |  |  |
|  | STAG2 |  |  |
|  | MIR23B |  |  |
|  | DLK1 |  |  |
|  | FXN |  |  |
|  | PDGFA |  |  |
|  | CDK5 |  |  |
|  | UMOD |  |  |
|  | BCKDHB |  |  |
|  | SPI1 |  |  |
|  | MIR376C |  |  |
|  | SEPSECS |  |  |
|  | C5 |  |  |
|  | COL4A6 |  |  |
|  | TRNT1 |  |  |
|  | GSR |  |  |
|  | MX1 |  |  |
|  | ACP5 |  |  |
|  | GREM1 |  |  |
|  | ATP6AP1 |  |  |
|  | AHCY |  |  |
|  | ABCC2 |  |  |
|  | IRF4 |  |  |
|  | CDK1 |  |  |
|  | IGES |  |  |
|  | RPGRIP1L |  |  |
|  | MIR133B |  |  |
|  | RPL11 |  |  |
|  | SLC25A20 |  |  |
|  | KIF5C |  |  |
|  | PIGG |  |  |
|  | DSG2 |  |  |
|  | KAT5 |  |  |
|  | SOD3 |  |  |
|  | GNB1 |  |  |
|  | LMO2 |  |  |
|  | GABRB2 |  |  |
|  | HSD17B10 |  |  |
|  | OTUD5 |  |  |
|  | TUBB4A |  |  |
|  | FLT4 |  |  |
|  | SNX14 |  |  |
|  | CACNA1H |  |  |
|  | KIF1B |  |  |
|  | MT-TS2 |  |  |
|  | PPFIBP1 |  |  |
|  | LAMA5 |  |  |
|  | SAMHD1 |  |  |
|  | MYH11 |  |  |
|  | FAAH |  |  |
|  | AHR |  |  |
|  | MORC2 |  |  |
|  | APRT |  |  |
|  | PDHX |  |  |
|  | TRAPPC2L |  |  |
|  | DNMT1 |  |  |
|  | TTC19 |  |  |
|  | SATB1 |  |  |
|  | HAVCR1 |  |  |
|  | RAB27A |  |  |
|  | ARID2 |  |  |
|  | AGL |  |  |
|  | CHRM3 |  |  |
|  | NEK1 |  |  |
|  | STX1A |  |  |
|  | CEACAM3 |  |  |
|  | VAC14 |  |  |
|  | TUSC3 |  |  |
|  | FLT3LG |  |  |
|  | ST7-OT3 |  |  |
|  | RNASE2 |  |  |
|  | SLC11A1 |  |  |
|  | ASL |  |  |
|  | LOC129931597 |  |  |
|  | HSPA8 |  |  |
|  | MIR122 |  |  |
|  | DEFB1 |  |  |
|  | HDAC4 |  |  |
|  | ORC6 |  |  |
|  | SLC25A13 |  |  |
|  | SPINK1 |  |  |
|  | MIR20A |  |  |
|  | PPP1CA |  |  |
|  | HACD1 |  |  |
|  | SAMD9L |  |  |
|  | HADHB |  |  |
|  | SYNE2 |  |  |
|  | MT-ATP8 |  |  |
|  | SLC35A2 |  |  |
|  | LZTFL1 |  |  |
|  | SNHG5 |  |  |
|  | COL4A2 |  |  |
|  | TBCE |  |  |
|  | COX5A |  |  |
|  | GABRA5 |  |  |
|  | STAT5A |  |  |
|  | IL17F |  |  |
|  | GDF15 |  |  |
|  | ORC1 |  |  |
|  | NCAM1 |  |  |
|  | IGFBP2 |  |  |
|  | CHRNA4 |  |  |
|  | TRIP4 |  |  |
|  | NOD1 |  |  |
|  | NCKAP1L |  |  |
|  | MIR487B |  |  |
|  | BCL6 |  |  |
|  | XRCC1 |  |  |
|  | EZH2 |  |  |
|  | DOLK |  |  |
|  | GALT |  |  |
|  | GEMIN4 |  |  |
|  | MAOB |  |  |
|  | IFT172 |  |  |
|  | P4HTM |  |  |
|  | NRP1 |  |  |
|  | RPS27A |  |  |
|  | B9D1 |  |  |
|  | GPX1 |  |  |
|  | VARS1 |  |  |
|  | PLOD2 |  |  |
|  | PTGS1 |  |  |
|  | LIG3 |  |  |
|  | LDB3 |  |  |
|  | PRSS1 |  |  |
|  | MIR141 |  |  |
|  | SGK1 |  |  |
|  | MITF |  |  |
|  | SLC9A3 |  |  |
|  | FGF20 |  |  |
|  | DPAGT1 |  |  |
|  | RAD50 |  |  |
|  | GRIA3 |  |  |
|  | GM2A |  |  |
|  | CFL1 |  |  |
|  | ITK |  |  |
|  | GORAB |  |  |
|  | ADAMTSL1 |  |  |
|  | NR1H4 |  |  |
|  | JUP |  |  |
|  | USP26 |  |  |
|  | UNG |  |  |
|  | HPS1 |  |  |
|  | POLR2A |  |  |
|  | OPN1LW |  |  |
|  | STAR |  |  |
|  | NRXN2 |  |  |
|  | CRLF1 |  |  |
|  | IGHG2 |  |  |
|  | P4HB |  |  |
|  | PYGM |  |  |
|  | TCTN3 |  |  |
|  | DLL1 |  |  |
|  | TP53BP1 |  |  |
|  | APTX |  |  |
|  | GNRH1 |  |  |
|  | DDC |  |  |
|  | SOX4 |  |  |
|  | DISC1 |  |  |
|  | PRKCA |  |  |
|  | NPM1 |  |  |
|  | FARSB |  |  |
|  | NDUFAF5 |  |  |
|  | GLUL |  |  |
|  | TAPBP |  |  |
|  | SOX2-OT |  |  |
|  | VIP |  |  |
|  | CBL |  |  |
|  | RAB8A |  |  |
|  | NOX4 |  |  |
|  | TAFAZZIN |  |  |
|  | RPL10 |  |  |
|  | TMEM231 |  |  |
|  | IREB2 |  |  |
|  | IRF2BP2 |  |  |
|  | HYAL1 |  |  |
|  | SMARCD1 |  |  |
|  | MAD1L1 |  |  |
|  | IL18BP |  |  |
|  | BBS4 |  |  |
|  | KCTD7 |  |  |
|  | MSH3 |  |  |
|  | AFP |  |  |
|  | ALS2 |  |  |
|  | H2AX |  |  |
|  | POU5F1 |  |  |
|  | BCYRN1 |  |  |
|  | MT-TW |  |  |
|  | CD38 |  |  |
|  | LAMB2 |  |  |
|  | SAA1 |  |  |
|  | CDKN3 |  |  |
|  | ASPM |  |  |
|  | ADAMTS2 |  |  |
|  | FZD4 |  |  |
|  | CSPP1 |  |  |
|  | MYH14 |  |  |
|  | AGPS |  |  |
|  | VKORC1 |  |  |
|  | TSR2 |  |  |
|  | ANXA11 |  |  |
|  | COPA |  |  |
|  | RNASEH2B |  |  |
|  | TBCB |  |  |
|  | FANCD2 |  |  |
|  | RAB7A |  |  |
|  | RBFOX1 |  |  |
|  | RPS10 |  |  |
|  | REL |  |  |
|  | CRYAB |  |  |
|  | PIGT |  |  |
|  | TSPAN1 |  |  |
|  | ATP12A |  |  |
|  | ANK3 |  |  |
|  | RPL36A-HNRNPH2 |  |  |
|  | DYNC2I1 |  |  |
|  | IL1RL1 |  |  |
|  | IPW |  |  |
|  | PTPN6 |  |  |
|  | RARB |  |  |
|  | NQO1 |  |  |
|  | SCN10A |  |  |
|  | GRM7 |  |  |
|  | TNC |  |  |
|  | MHRT |  |  |
|  | HABP2 |  |  |
|  | LOC111674475 |  |  |
|  | BCKDHA |  |  |
|  | MED12 |  |  |
|  | MIR200C |  |  |
|  | KCNJ13 |  |  |
|  | TRB |  |  |
|  | LAS1L |  |  |
|  | HLA-DMA |  |  |
|  | HRH2 |  |  |
|  | SYNJ1 |  |  |
|  | MAVS |  |  |
|  | IGBP1 |  |  |
|  | CALR |  |  |
|  | FTL |  |  |
|  | AMT |  |  |
|  | DHODH |  |  |
|  | IGF2R |  |  |
|  | MSH6 |  |  |
|  | TSPAN12 |  |  |
|  | ZFAS1 |  |  |
|  | LY96 |  |  |
|  | MIR200A |  |  |
|  | PAPPA |  |  |
|  | ITGA2 |  |  |
|  | PMPCA |  |  |
|  | YAP1 |  |  |
|  | REEP1 |  |  |
|  | CUL4B |  |  |
|  | PI3 |  |  |
|  | SYNE1 |  |  |
|  | TCF12 |  |  |
|  | ATXN1 |  |  |
|  | GAS8 |  |  |
|  | LEPQTL1 |  |  |
|  | NPHP1 |  |  |
|  | AKT2 |  |  |
|  | ABCG1 |  |  |
|  | P2RX7 |  |  |
|  | MIR25 |  |  |
|  | VDAC1 |  |  |
|  | HSP90AA1 |  |  |
|  | DUSP1 |  |  |
|  | NPHS2 |  |  |
|  | MEG8 |  |  |
|  | TMEM237 |  |  |
|  | AFF4 |  |  |
|  | FKBP14 |  |  |
|  | DPM1 |  |  |
|  | CETP |  |  |
|  | LOC106029312 |  |  |
|  | WDR81 |  |  |
|  | UPF1 |  |  |
|  | BTD |  |  |
|  | CAV3 |  |  |
|  | SLC6A1 |  |  |
|  | CHAMP1 |  |  |
|  | KDM5B |  |  |
|  | PIGR |  |  |
|  | STOX1 |  |  |
|  | MSX1 |  |  |
|  | ASCL1 |  |  |
|  | LYN |  |  |
|  | CARMN |  |  |
|  | ADAM33 |  |  |
|  | KANSL1 |  |  |
|  | GSS |  |  |
|  | FBXW7 |  |  |
|  | GZMA |  |  |
|  | LRP12 |  |  |
|  | TRIM32 |  |  |
|  | AVPR2 |  |  |
|  | SNORD118 |  |  |
|  | SMARCAL1 |  |  |
|  | KCNIP1 |  |  |
|  | B4GALT7 |  |  |
|  | PLCB1 |  |  |
|  | PRKAG2 |  |  |
|  | ATF6 |  |  |
|  | SIM1 |  |  |
|  | ABCG5 |  |  |
|  | NPHP3 |  |  |
|  | TONSL |  |  |
|  | UROS |  |  |
|  | HBG1 |  |  |
|  | ATP6V1B2 |  |  |
|  | AFF2 |  |  |
|  | SMARCC2 |  |  |
|  | GAN |  |  |
|  | ZNHIT3 |  |  |
|  | ENTREP2 |  |  |
|  | ANKRD11 |  |  |
|  | HSPA1A |  |  |
|  | ZNF341 |  |  |
|  | FAS-AS1 |  |  |
|  | CEBPB |  |  |
|  | TPMT |  |  |
|  | GIPC1 |  |  |
|  | IGFBP5 |  |  |
|  | SMCHD1 |  |  |
|  | MC2R |  |  |
|  | PPBP |  |  |
|  | TBCD |  |  |
|  | LSS |  |  |
|  | MAP2 |  |  |
|  | CHKB |  |  |
|  | ODAD4 |  |  |
|  | DISP1 |  |  |
|  | MPDU1 |  |  |
|  | HLA-E |  |  |
|  | MT-ND4L |  |  |
|  | RYR2 |  |  |
|  | MCPH1 |  |  |
|  | CMA1 |  |  |
|  | TREM2 |  |  |
|  | EXT2 |  |  |
|  | PSMB1 |  |  |
|  | KCNE1 |  |  |
|  | ARF1 |  |  |
|  | CLCN2 |  |  |
|  | MICB |  |  |
|  | BANF1 |  |  |
|  | ATP4A |  |  |
|  | ARFGEF2 |  |  |
|  | RAB3GAP2 |  |  |
|  | PGAP2 |  |  |
|  | CUBN |  |  |
|  | PAK2 |  |  |
|  | PGM1 |  |  |
|  | PDE6B |  |  |
|  | RPS29 |  |  |
|  | AASS |  |  |
|  | MIR493 |  |  |
|  | LOC107303340 |  |  |
|  | ENTPD1 |  |  |
|  | HUWE1 |  |  |
|  | DRC1 |  |  |
|  | C1QB |  |  |
|  | RELB |  |  |
|  | DDR1 |  |  |
|  | QDPR |  |  |
|  | MIRLET7G |  |  |
|  | MRE11 |  |  |
|  | CCL24 |  |  |
|  | HCCAT5 |  |  |
|  | POMK |  |  |
|  | ALG3 |  |  |
|  | MIR26B |  |  |
|  | MANBA |  |  |
|  | MIRLET7I |  |  |
|  | PSMB4 |  |  |
|  | GCLC |  |  |
|  | FCN2 |  |  |
|  | TPI1 |  |  |
|  | HFE-AS1 |  |  |
|  | SLC5A7 |  |  |
|  | STX11 |  |  |
|  | AMPD1 |  |  |
|  | SMAD7 |  |  |
|  | CACNA1G-AS1 |  |  |
|  | MIR19A |  |  |
|  | MIR483 |  |  |
|  | NOTCH2NLC |  |  |
|  | CHRNA3 |  |  |
|  | NDUFAF2 |  |  |
|  | DHX9 |  |  |
|  | CSF2RA |  |  |
|  | HLA-DPA1 |  |  |
|  | MIR362 |  |  |
|  | GHRH |  |  |
|  | PRPF31 |  |  |
|  | MIR200B |  |  |
|  | HMBS |  |  |
|  | C1QA |  |  |
|  | LOC108167311 |  |  |
|  | LOC108167312 |  |  |
|  | PRODH |  |  |
|  | APLN |  |  |
|  | MIR323A |  |  |
|  | SERPING1 |  |  |
|  | NDN |  |  |
|  | RHD |  |  |
|  | TMEM38B |  |  |
|  | TSPO |  |  |
|  | PVALB |  |  |
|  | NARS2 |  |  |
|  | RPS24 |  |  |
|  | UMPS |  |  |
|  | MIR485 |  |  |
|  | ATG16L1 |  |  |
|  | PMS2 |  |  |
|  | CCL26 |  |  |
|  | ARMS2 |  |  |
|  | DGCR5 |  |  |
|  | CCL27 |  |  |
|  | TBX21 |  |  |
|  | MUC7 |  |  |
|  | DRD1 |  |  |
|  | ADAM9 |  |  |
|  | CSMD1 |  |  |
|  | PABPN1 |  |  |
|  | MT-TI |  |  |
|  | MKRN3 |  |  |
|  | RPS26 |  |  |
|  | DAOA-AS1 |  |  |
|  | MUSK |  |  |
|  | IL10RB |  |  |
|  | DZIP1L |  |  |
|  | MIR501 |  |  |
|  | IL37 |  |  |
|  | SNRPB |  |  |
|  | UBE2A |  |  |
|  | SNRNP70 |  |  |
|  | RAB18 |  |  |
|  | CLCN5 |  |  |
|  | MUC16 |  |  |
|  | SCARB1 |  |  |
|  | DOCK8-AS1 |  |  |
|  | DNA2 |  |  |
|  | KL |  |  |
|  | YWHAE |  |  |
|  | PF4 |  |  |
|  | CPLX1 |  |  |
|  | GTF2H5 |  |  |
|  | ODAD2 |  |  |
|  | PKP1 |  |  |
|  | BACE1 |  |  |
|  | DNAAF4 |  |  |
|  | MIR15B |  |  |
|  | IGKC |  |  |
|  | LOC130001437 |  |  |
|  | TUBA8 |  |  |
|  | TUBB2A |  |  |
|  | GP1BB |  |  |
|  | CPT1A |  |  |
|  | MUC2 |  |  |
|  | IDH2 |  |  |
|  | HSP90B1 |  |  |
|  | LEMD2 |  |  |
|  | SHOC2 |  |  |
|  | PRSS2 |  |  |
|  | OSM |  |  |
|  | CDC6 |  |  |
|  | GIGYF2 |  |  |
|  | MKI67 |  |  |
|  | PIGU |  |  |
|  | POR |  |  |
|  | TIMP2 |  |  |
|  | KRT17 |  |  |
|  | KLK3 |  |  |
|  | CHEK1 |  |  |
|  | MPV17 |  |  |
|  | HSPA1B |  |  |
|  | LOC126860552 |  |  |
|  | POLG2 |  |  |
|  | ARSG |  |  |
|  | CDSN |  |  |
|  | TRRAP |  |  |
|  | CPLANE1 |  |  |
|  | FANCC |  |  |
|  | HLA-DMB |  |  |
|  | PCSK1 |  |  |
|  | CYP1A2 |  |  |
|  | CNTF |  |  |
|  | RN7SL1 |  |  |
|  | THY1 |  |  |
|  | AGXT |  |  |
|  | CANX |  |  |
|  | DDX41 |  |  |
|  | FIP1L1 |  |  |
|  | SF3B1 |  |  |
|  | HNRNPK |  |  |
|  | ARL3 |  |  |
|  | DNAH9 |  |  |
|  | FOXE3 |  |  |
|  | PITX2 |  |  |
|  | CRKL |  |  |
|  | ANG |  |  |
|  | ACVRL1 |  |  |
|  | CCL21 |  |  |
|  | CX3CR1 |  |  |
|  | ABCB7 |  |  |
|  | AXL |  |  |
|  | ITLN1 |  |  |
|  | PCYT1A |  |  |
|  | TRP-AGG2-5 |  |  |
|  | CDH5 |  |  |
|  | FCER2 |  |  |
|  | CHGA |  |  |
|  | CTCF |  |  |
|  | PHB2 |  |  |
|  | AP3D1 |  |  |
|  | HDAC2 |  |  |
|  | TBC1D20 |  |  |
|  | MAP2K2 |  |  |
|  | PHYH |  |  |
|  | MIR24-1 |  |  |
|  | B3GAT3 |  |  |
|  | SOS1 |  |  |
|  | SERPINA3 |  |  |
|  | MMUT |  |  |
|  | RPS17 |  |  |
|  | EIF2S3 |  |  |
|  | DNAJB4 |  |  |
|  | MIR10A |  |  |
|  | GLRA2 |  |  |
|  | RNPC3 |  |  |
|  | CCL7 |  |  |
|  | CHRNA5 |  |  |
|  | MESP2 |  |  |
|  | SLC12A2 |  |  |
|  | MT-TD |  |  |
|  | PIGK |  |  |
|  | EXOSC9 |  |  |
|  | FABP2 |  |  |
|  | LITAF |  |  |
|  | CCDC39 |  |  |
|  | PLCH1 |  |  |
|  | ESM1 |  |  |
|  | PRIM1 |  |  |
|  | LOC126805877 |  |  |
|  | SPEN |  |  |
|  | EPG5 |  |  |
|  | RNASEH2A |  |  |
|  | CXCR6 |  |  |
|  | P2RY12 |  |  |
|  | ANXA2 |  |  |
|  | CEP104 |  |  |
|  | CLEC12A |  |  |
|  | PXDNL |  |  |
|  | NPPC |  |  |
|  | PSORS1C1 |  |  |
|  | WDR26 |  |  |
|  | MTUS1 |  |  |
|  | LRSAM1 |  |  |
|  | KPNA2 |  |  |
|  | IVL |  |  |
|  | CASC2 |  |  |
|  | IFI44 |  |  |
|  | GNPTG |  |  |
|  | NFASC |  |  |
|  | CYP27A1 |  |  |
|  | LMNB2 |  |  |
|  | BRD4 |  |  |
|  | CD27-AS1 |  |  |
|  | PLEK |  |  |
|  | NBEA |  |  |
|  | WHRN |  |  |
|  | CXCR5 |  |  |
|  | COG8 |  |  |
|  | CLEC6A |  |  |
|  | PIGL |  |  |
|  | TNFSF10 |  |  |
|  | NCF4-AS1 |  |  |
|  | LOC126861897 |  |  |
|  | CCN4 |  |  |
|  | SERAC1 |  |  |
|  | CALM2 |  |  |
|  | ROR2 |  |  |
|  | DLEU2 |  |  |
|  | IFNL3 |  |  |
|  | DYNLT2B |  |  |
|  | MTUS2 |  |  |
|  | HLA-DRB5 |  |  |
|  | LIAS |  |  |
|  | ALPP |  |  |
|  | SLC25A24 |  |  |
|  | OTOA |  |  |
|  | DHCR24 |  |  |
|  | CPA6 |  |  |
|  | ABCG2 |  |  |
|  | MAP1B |  |  |
|  | MT-TN |  |  |
|  | MIR107 |  |  |
|  | PTCD3 |  |  |
|  | TRIM8 |  |  |
|  | TRAPPC12 |  |  |
|  | COPB2 |  |  |
|  | ACER3 |  |  |
|  | TIMM8A |  |  |
|  | ADK |  |  |
|  | RARA |  |  |
|  | TLR10 |  |  |
|  | RPL15 |  |  |
|  | TMEM216 |  |  |
|  | ALDH2 |  |  |
|  | MT-TH |  |  |
|  | PLA2G4A |  |  |
|  | OSGEP |  |  |
|  | MASP1 |  |  |
|  | TUFM |  |  |
|  | PSEN2 |  |  |
|  | PDE4A |  |  |
|  | NOTCH2 |  |  |
|  | FANCB |  |  |
|  | PPP1R12C |  |  |
|  | FAT1 |  |  |
|  | PSMA6 |  |  |
|  | MT-TP |  |  |
|  | PIGQ |  |  |
|  | EIF2AK3 |  |  |
|  | LOC129999940 |  |  |
|  | ERC1 |  |  |
|  | FOXM1 |  |  |
|  | NME5 |  |  |
|  | IKBKE |  |  |
|  | KLRK1 |  |  |
|  | D2HGDH |  |  |
|  | MT-TQ |  |  |
|  | VPS13D |  |  |
|  | PPIA |  |  |
|  | EPHB4 |  |  |
|  | GMNN |  |  |
|  | HTR3A |  |  |
|  | ATP13A2 |  |  |
|  | ATP2A2 |  |  |
|  | CASP9 |  |  |
|  | ATP6V1B1 |  |  |
|  | UGDH |  |  |
|  | PTPN1 |  |  |
|  | LOC126862902 |  |  |
|  | UGP2 |  |  |
|  | OPTN |  |  |
|  | SMO |  |  |
|  | MYPN |  |  |
|  | SNAI1 |  |  |
|  | LOC130058751 |  |  |
|  | AGPAT2 |  |  |
|  | MIR16-1 |  |  |
|  | ZBTB24 |  |  |
|  | NFU1 |  |  |
|  | ELP4 |  |  |
|  | KNSTRN |  |  |
|  | TRAPPC2 |  |  |
|  | RERE |  |  |
|  | PLCG1 |  |  |
|  | NEK9 |  |  |
|  | CIT |  |  |
|  | MCCC2 |  |  |
|  | MT-TR |  |  |
|  | IFT80 |  |  |
|  | SLC2A10 |  |  |
|  | FGF9 |  |  |
|  | SGCE |  |  |
|  | FGF3 |  |  |
|  | CEP250 |  |  |
|  | NR1H2 |  |  |
|  | SZT2 |  |  |
|  | F2RL1 |  |  |
|  | STT3B |  |  |
|  | LYSET |  |  |
|  | TMPO |  |  |
|  | EZR |  |  |
|  | EDA |  |  |
|  | LRP1 |  |  |
|  | F12 |  |  |
|  | WNK1 |  |  |
|  | SLC35A1 |  |  |
|  | KCNB1 |  |  |
|  | IAPP |  |  |
|  | SRY |  |  |
|  | GC |  |  |
|  | CTBP1 |  |  |
|  | YY1 |  |  |
|  | SLC12A1 |  |  |
|  | CDC42BPB |  |  |
|  | RECK |  |  |
|  | GRM1 |  |  |
|  | IL23R |  |  |
|  | CEP120 |  |  |
|  | ATP6V1E1 |  |  |
|  | CLCN4 |  |  |
|  | MIR188 |  |  |
|  | CYP24A1 |  |  |
|  | MAP3K20 |  |  |
|  | GRK2 |  |  |
|  | HTR2C |  |  |
|  | CHST14 |  |  |
|  | HULC |  |  |
|  | RPS20 |  |  |
|  | ADRB3 |  |  |
|  | SAG |  |  |
|  | HIBCH |  |  |
|  | LOC130008987 |  |  |
|  | MIR100 |  |  |
|  | NTRK3 |  |  |
|  | LCP2 |  |  |
|  | RPS6KB1 |  |  |
|  | CCL15-CCL14 |  |  |
|  | NSMCE2 |  |  |
|  | VCAN |  |  |
|  | FRAXA |  |  |
|  | FGF17 |  |  |
|  | VPS4A |  |  |
|  | HEXB |  |  |
|  | GAL |  |  |
|  | EXOSC8 |  |  |
|  | ADCY10 |  |  |
|  | MT-TC |  |  |
|  | SLC1A3 |  |  |
|  | SEC23B |  |  |
|  | NLRP1 |  |  |
|  | HSPA9 |  |  |
|  | TNRC6B |  |  |
|  | FASN |  |  |
|  | NPAP1 |  |  |
|  | NCR3 |  |  |
|  | FERMT3 |  |  |
|  | ALOXE3 |  |  |
|  | SLC40A1 |  |  |
|  | SHANK2 |  |  |
|  | PROP1 |  |  |
|  | LOC107988032 |  |  |
|  | POLE |  |  |
|  | SIL1 |  |  |
|  | SLC3A1 |  |  |
|  | IL9R |  |  |
|  | SDC1 |  |  |
|  | BTNL2 |  |  |
|  | DLG4 |  |  |
|  | MSMB |  |  |
|  | DLL3 |  |  |
|  | BCL2L1 |  |  |
|  | EDN3 |  |  |
|  | RAB35 |  |  |
|  | NADK2 |  |  |
|  | TFE3 |  |  |
|  | GPT2 |  |  |
|  | XK |  |  |
|  | GSTM3 |  |  |
|  | TRIM2 |  |  |
|  | GHSR |  |  |
|  | INVS |  |  |
|  | IFT74 |  |  |
|  | SIAH1 |  |  |
|  | LOC129994126 |  |  |
|  | WASHC5 |  |  |
|  | FANCF |  |  |
|  | MYOD1 |  |  |
|  | HADH |  |  |
|  | PARK7 |  |  |
|  | AURKA |  |  |
|  | FLII |  |  |
|  | PHKA2 |  |  |
|  | LARP7 |  |  |
|  | PIK3CB |  |  |
|  | SKIC3 |  |  |
|  | SRCAP |  |  |
|  | SEMA3E |  |  |
|  | PKP2 |  |  |
|  | DDX39B |  |  |
|  | TYMP |  |  |
|  | PIGW |  |  |
|  | DLG1 |  |  |
|  | MT-TM |  |  |
|  | ODC1 |  |  |
|  | DCC |  |  |
|  | EXT1 |  |  |
|  | FARS2 |  |  |
|  | MT-TA |  |  |
|  | SDCCAG8 |  |  |
|  | UBB |  |  |
|  | PTS |  |  |
|  | FCRL6 |  |  |
|  | ACP1 |  |  |
|  | CTC1 |  |  |
|  | MIR494 |  |  |
|  | FYCO1 |  |  |
|  | MPZL1 |  |  |
|  | RASGRP1 |  |  |
|  | HELLS |  |  |
|  | PRMT7 |  |  |
|  | ITPR1 |  |  |
|  | AP1S2 |  |  |
|  | TET3 |  |  |
|  | LRP2 |  |  |
|  | LOC130068796 |  |  |
|  | BBS7 |  |  |
|  | MT-TG |  |  |
|  | UTRN |  |  |
|  | SELPLG |  |  |
|  | KCNJ1 |  |  |
|  | HOXA13 |  |  |
|  | PLCB4 |  |  |
|  | CRIPT |  |  |
|  | DVL1 |  |  |
|  | LEMD3 |  |  |
|  | MCL1 |  |  |
|  | LAG3 |  |  |
|  | CLPP |  |  |
|  | AMFR |  |  |
|  | TRMU |  |  |
|  | RYR3 |  |  |
|  | LMAN1 |  |  |
|  | COLQ |  |  |
|  | SCT |  |  |
|  | MOGS |  |  |
|  | DCHS1 |  |  |
|  | MIR196A1 |  |  |
|  | SULT1A3 |  |  |
|  | OGG1 |  |  |
|  | GYG1 |  |  |
|  | SLC6A2 |  |  |
|  | DNAJB2 |  |  |
|  | IFT56 |  |  |
|  | RNASET2 |  |  |
|  | RNF2 |  |  |
|  | C19orf12 |  |  |
|  | PWRN1 |  |  |
|  | NT5E |  |  |
|  | CCAT1 |  |  |
|  | KRT19 |  |  |
|  | DYM |  |  |
|  | CSF2RB |  |  |
|  | ACADL |  |  |
|  | CASP6 |  |  |
|  | FAT4 |  |  |
|  | RARRES2 |  |  |
|  | WDFY3 |  |  |
|  | PWAR4 |  |  |
|  | RFX1 |  |  |
|  | CSN1S1 |  |  |
|  | NIPAL4 |  |  |
|  | NEDD4 |  |  |
|  | ASCC1 |  |  |
|  | ALG6 |  |  |
|  | ODAD1 |  |  |
|  | ZBTB7A |  |  |
|  | HSPB8 |  |  |
|  | PDE5A |  |  |
|  | STX6 |  |  |
|  | CHCHD10 |  |  |
|  | TPSAB1 |  |  |
|  | BIVM-ERCC5 |  |  |
|  | FABP4 |  |  |
|  | TBX3 |  |  |
|  | MIR128-1 |  |  |
|  | HDAC6 |  |  |
|  | STX1B |  |  |
|  | MPLKIP |  |  |
|  | CEP164 |  |  |
|  | SCARB2 |  |  |
|  | MIR181A1 |  |  |
|  | ITGA3 |  |  |
|  | SYT1 |  |  |
|  | TRIO |  |  |
|  | ZNFX1 |  |  |
|  | VPS13C |  |  |
|  | PIK3R2 |  |  |
|  | LOC109504725 |  |  |
|  | GOSR2 |  |  |
|  | CCL19 |  |  |
|  | SLC10A7 |  |  |
|  | CADM1 |  |  |
|  | TFEB |  |  |
|  | CDK2 |  |  |
|  | FANCL |  |  |
|  | OR1F1 |  |  |
|  | SI |  |  |
|  | NAGS |  |  |
|  | WLS |  |  |
|  | NDUFS6 |  |  |
|  | ARSH |  |  |
|  | LGALS9 |  |  |
|  | NRCAM |  |  |
|  | NALT1 |  |  |
|  | BCORL1 |  |  |
|  | MIR328 |  |  |
|  | TNFRSF9 |  |  |
|  | VPS11 |  |  |
|  | CHKB-CPT1B |  |  |
|  | ALOX12B |  |  |
|  | FER |  |  |
|  | CDC45 |  |  |
|  | MYO1H |  |  |
|  | LYZ |  |  |
|  | CD1A |  |  |
|  | GABRB1 |  |  |
|  | PCDH10 |  |  |
|  | GK |  |  |
|  | IGH |  |  |
|  | TMPPE |  |  |
|  | HTR6 |  |  |
|  | HARS1 |  |  |
|  | HBEGF |  |  |
|  | STAC3 |  |  |
|  | HPSE2 |  |  |
|  | FGF6 |  |  |
|  | NDUFA9 |  |  |
|  | MIR708 |  |  |
|  | PAX7 |  |  |
|  | ARHGEF10 |  |  |
|  | HYOU1 |  |  |
|  | LOXL1 |  |  |
|  | DRD3 |  |  |
|  | PLVAP |  |  |
|  | TBX20 |  |  |
|  | PIGY |  |  |
|  | PABPC1 |  |  |
|  | CSNK2A2 |  |  |
|  | CAVIN1 |  |  |
|  | LOC126863256 |  |  |
|  | HNF1A-AS1 |  |  |
|  | HNRNPA1 |  |  |
|  | ETV6 |  |  |
|  | MGAM |  |  |
|  | EDAR |  |  |
|  | SLC5A1 |  |  |
|  | SLC5A6 |  |  |
|  | POLR1D |  |  |
|  | DHX37 |  |  |
|  | GGCX |  |  |
|  | RPL26 |  |  |
|  | MIR195 |  |  |
|  | PAX9 |  |  |
|  | PLA2G1B |  |  |
|  | CYSLTR1 |  |  |
|  | SPEG |  |  |
|  | TBC1D5 |  |  |
|  | GCM2 |  |  |
|  | CTSK |  |  |
|  | LOXL2 |  |  |
|  | PRKG2 |  |  |
|  | CXCL11 |  |  |
|  | TRAF2 |  |  |
|  | CD276 |  |  |
|  | MIR363 |  |  |
|  | MST1R |  |  |
|  | COLGALT1 |  |  |
|  | ACAD8 |  |  |
|  | IER3IP1 |  |  |
|  | PTHLH |  |  |
|  | EGR1 |  |  |
|  | IFT81 |  |  |
|  | VPS33B |  |  |
|  | GLP1R |  |  |
|  | ADAMTS3 |  |  |
|  | KRT12 |  |  |
|  | SLC39A8 |  |  |
|  | NBAS |  |  |
|  | TSEN2 |  |  |
|  | NNT-AS1 |  |  |
|  | ADCY3 |  |  |
|  | CFLAR |  |  |
|  | MIR183 |  |  |
|  | UGCG |  |  |
|  | STK36 |  |  |
|  | NLRP12 |  |  |
|  | DSG4 |  |  |
|  | TPT1 |  |  |
|  | HDAC1 |  |  |
|  | ACAT1 |  |  |
|  | PSTPIP1 |  |  |
|  | PALB2 |  |  |
|  | OGA |  |  |
|  | TUBG1 |  |  |
|  | SMAD6 |  |  |
|  | NGFR |  |  |
|  | CTNNA3 |  |  |
|  | CD52 |  |  |
|  | VIPAS39 |  |  |
|  | SH2B3 |  |  |
|  | FCAR |  |  |
|  | SMC5 |  |  |
|  | TLR8 |  |  |
|  | UBE4A |  |  |
|  | MOG |  |  |
|  | NTNG1 |  |  |
|  | FLCN |  |  |
|  | GAS1 |  |  |
|  | RNU7-1 |  |  |
|  | TRIM25 |  |  |
|  | UTP4 |  |  |
|  | TCTN2 |  |  |
|  | PSMC1 |  |  |
|  | SPTBN2 |  |  |
|  | ATXN8OS |  |  |
|  | EMC1 |  |  |
|  | SYP |  |  |
|  | PSMB9 |  |  |
|  | TEK |  |  |
|  | COQ8B |  |  |
|  | CDKN2B |  |  |
|  | SLC35D1 |  |  |
|  | MIR370 |  |  |
|  | ATXN7 |  |  |
|  | CALM3 |  |  |
|  | ALG11 |  |  |
|  | DNAAF2 |  |  |
|  | TARS1 |  |  |
|  | VAPB |  |  |
|  | CRHR1 |  |  |
|  | TAPBPL |  |  |
|  | STUB1 |  |  |
|  | DNAH1 |  |  |
|  | NEK8 |  |  |
|  | MSH5 |  |  |
|  | TEC |  |  |
|  | AREG |  |  |
|  | NDEL1 |  |  |
|  | CEBPA |  |  |
|  | PIKFYVE |  |  |
|  | RPA1 |  |  |
|  | GLUD1 |  |  |
|  | ATP6V0A1 |  |  |
|  | AHDC1 |  |  |
|  | UBE3B |  |  |
|  | KRT3 |  |  |
|  | LAMA4 |  |  |
|  | CENPF |  |  |
|  | MIR32 |  |  |
|  | SPTBN1 |  |  |
|  | TAB2 |  |  |
|  | IL12RB2 |  |  |
|  | LOC126862264 |  |  |
|  | GPR55 |  |  |
|  | DUX4 |  |  |
|  | PERCC1 |  |  |
|  | HLA-DRA |  |  |
|  | GPD1 |  |  |
|  | RPS27 |  |  |
|  | TNPO3 |  |  |
|  | PTPRO |  |  |
|  | TJP1 |  |  |
|  | NANS |  |  |
|  | MIR211 |  |  |
|  | TRAF1 |  |  |
|  | LOC126862864 |  |  |
|  | MYH3 |  |  |
|  | MDC1 |  |  |
|  | ADIPOR2 |  |  |
|  | FH |  |  |
|  | PKM |  |  |
|  | SALL1 |  |  |
|  | LIG1 |  |  |
|  | AKAP9 |  |  |
|  | GOLGA2 |  |  |
|  | TRE-TTC3-1 |  |  |
|  | MEIG1 |  |  |
|  | TRC-GCA24-1 |  |  |
|  | TNFSF4 |  |  |
|  | IKZF3 |  |  |
|  | KDM1A |  |  |
|  | GTPBP2 |  |  |
|  | KIAA0753 |  |  |
|  | FAH |  |  |
|  | TLL1 |  |  |
|  | NSUN2 |  |  |
|  | XCR1 |  |  |
|  | ACVR1 |  |  |
|  | RXRA |  |  |
|  | NR1H3 |  |  |
|  | SLC11A2 |  |  |
|  | SPART |  |  |
|  | PLEKHM1 |  |  |
|  | KATNIP |  |  |
|  | MIR9-1 |  |  |
|  | LRPPRC |  |  |
|  | SETD1A |  |  |
|  | ARSK |  |  |
|  | SNX10 |  |  |
|  | SERPINA7 |  |  |
|  | FOXC2 |  |  |
|  | SAA4 |  |  |
|  | MCM5 |  |  |
|  | MIR491 |  |  |
|  | MIR103A2 |  |  |
|  | IL13RA1 |  |  |
|  | MYO5A |  |  |
|  | GP9 |  |  |
|  | SCARF2 |  |  |
|  | LOC130003418 |  |  |
|  | COX20 |  |  |
|  | CCL20 |  |  |
|  | ALAD |  |  |
|  | FERMT1 |  |  |
|  | KRT8 |  |  |
|  | AZU1 |  |  |
|  | PCCB |  |  |
|  | FGF4 |  |  |
|  | MC3R |  |  |
|  | CYTH1 |  |  |
|  | DNAI2 |  |  |
|  | SLC39A7 |  |  |
|  | MSR1 |  |  |
|  | FGF21 |  |  |
|  | CGA |  |  |
|  | SLC19A3 |  |  |
|  | BIRC3 |  |  |
|  | MSTN |  |  |
|  | RAP1A |  |  |
|  | BRIP1 |  |  |
|  | TSEN34 |  |  |
|  | NFS1 |  |  |
|  | APBB1 |  |  |
|  | RFT1 |  |  |
|  | IL27 |  |  |
|  | ATG5 |  |  |
|  | RPS28 |  |  |
|  | DDIT3 |  |  |
|  | ORMDL3 |  |  |
|  | LZTR1 |  |  |
|  | FABP3 |  |  |
|  | FUT2 |  |  |
|  | TWIST1 |  |  |
|  | VPS51 |  |  |
|  | MYO5B |  |  |
|  | GRHL3 |  |  |
|  | ADAM10 |  |  |
|  | CCR8 |  |  |
|  | APOL1 |  |  |
|  | RPL31 |  |  |
|  | CYP2A6 |  |  |
|  | ITCH |  |  |
|  | ATRIP-TREX1 |  |  |
|  | FANCI |  |  |
|  | ZFPM2 |  |  |
|  | OPHN1 |  |  |
|  | KCNJ6-AS1 |  |  |
|  | SERPINI1 |  |  |
|  | RPL22 |  |  |
|  | DSE |  |  |
|  | CEP135 |  |  |
|  | TFPI |  |  |
|  | FGF18 |  |  |
|  | WFDC2 |  |  |
|  | PIGH |  |  |
|  | LRPAP1 |  |  |
|  | SLURP1 |  |  |
|  | CARM1 |  |  |
|  | MT-TY |  |  |
|  | KPNA3 |  |  |
|  | COG4 |  |  |
|  | TMSB4X |  |  |
|  | PTPN3 |  |  |
|  | HSPA1L |  |  |
|  | ALOX5AP |  |  |
|  | WNT7B |  |  |
|  | SUMO1 |  |  |
|  | PLCE1 |  |  |
|  | SMARCA5 |  |  |
|  | SSB |  |  |
|  | SPECC1L |  |  |
|  | ARID4A |  |  |
|  | DSC2 |  |  |
|  | HGD |  |  |
|  | TERF1 |  |  |
|  | FOSL1 |  |  |
|  | FANCE |  |  |
|  | LSM11 |  |  |
|  | ACKR3 |  |  |
|  | RASAL3 |  |  |
|  | AIF1 |  |  |
|  | TAF6 |  |  |
|  | LSM2 |  |  |
|  | LIMK1 |  |  |
|  | SLC30A9 |  |  |
|  | COASY |  |  |
|  | MIR139 |  |  |
|  | NDUFA6 |  |  |
|  | PHIP |  |  |
|  | MIR151A |  |  |
|  | INSL3 |  |  |
|  | EIF3F |  |  |
|  | ADCYAP1 |  |  |
|  | FOXA2 |  |  |
|  | MIR378A |  |  |
|  | MMP10 |  |  |
|  | AGA |  |  |
|  | H3C1 |  |  |
|  | TRDN |  |  |
|  | TRAPPC4 |  |  |
|  | APEX1 |  |  |
|  | MED1 |  |  |
|  | PPP4C |  |  |
|  | IMPG1 |  |  |
|  | NR0B1 |  |  |
|  | CLTC |  |  |
|  | MGP |  |  |
|  | C1QC |  |  |
|  | TCEAL1 |  |  |
|  | RABIF |  |  |
|  | ACVR2A |  |  |
|  | CCBE1 |  |  |
|  | ANGPTL3 |  |  |
|  | ADCY6 |  |  |
|  | HPSE |  |  |
|  | SLC46A1 |  |  |
|  | STX2 |  |  |
|  | GOT2 |  |  |
|  | RACK1 |  |  |
|  | MBNL1 |  |  |
|  | TXNRD2 |  |  |
|  | E2F1 |  |  |
|  | KRT7 |  |  |
|  | UROD |  |  |
|  | TBL1XR1 |  |  |
|  | LCT |  |  |
|  | NUP214 |  |  |
|  | FRMD7 |  |  |
|  | IVNS1ABP |  |  |
|  | BCL2L11 |  |  |
|  | CLASP1 |  |  |
|  | AGRP |  |  |
|  | H1-4 |  |  |
|  | LY86 |  |  |
|  | ICMT |  |  |
|  | HIRA |  |  |
|  | MIR215 |  |  |
|  | ALG8 |  |  |
|  | ANXA6 |  |  |
|  | MIR185 |  |  |
|  | TGFA |  |  |
|  | PDYN |  |  |
|  | DOCK6 |  |  |
|  | PGBD3 |  |  |
|  | LRRC8A |  |  |
|  | INPPL1 |  |  |
|  | ISCU |  |  |
|  | TACR1 |  |  |
|  | FCSK |  |  |
|  | FASTKD2 |  |  |
|  | NOTCH4 |  |  |
|  | PMVK |  |  |
|  | NTS |  |  |
|  | GNAI1 |  |  |
|  | MIR424 |  |  |
|  | DSG3 |  |  |
|  | XBP1 |  |  |
|  | LTB |  |  |
|  | CACNA2D1 |  |  |
|  | TGM5 |  |  |
|  | CASQ2 |  |  |
|  | PRKACA |  |  |
|  | PSMD8 |  |  |
|  | HMOX2 |  |  |
|  | UBC |  |  |
|  | THRA |  |  |
|  | CD59 |  |  |
|  | IL32 |  |  |
|  | CRIPTO |  |  |
|  | BPIFA1 |  |  |
|  | MAN1B1 |  |  |
|  | OGT |  |  |
|  | ZIC3 |  |  |
|  | PSORS1C3 |  |  |
|  | EYA4 |  |  |
|  | HLCS |  |  |
|  | LAMA1 |  |  |
|  | RNF8 |  |  |
|  | RPS14 |  |  |
|  | ITPR3 |  |  |
|  | HYDIN |  |  |
|  | SRP72 |  |  |
|  | MORC3 |  |  |
|  | GLRX5 |  |  |
|  | UCP1 |  |  |
|  | CHMP2B |  |  |
|  | PLK4 |  |  |
|  | AGK |  |  |
|  | CYP4F22 |  |  |
|  | DNASE2 |  |  |
|  | MTO1 |  |  |
|  | GAST |  |  |
|  | AQP5 |  |  |
|  | PET100 |  |  |
|  | SLC9A1 |  |  |
|  | CEP128 |  |  |
|  | FOXF1 |  |  |
|  | DIAPH1 |  |  |
|  | SLC29A3 |  |  |
|  | NFKBIL1 |  |  |
|  | IFT88 |  |  |
|  | GPX2 |  |  |
|  | ARSL |  |  |
|  | GPC3 |  |  |
|  | IRX2-DT |  |  |
|  | GBA2 |  |  |
|  | SCAMP5 |  |  |
|  | SLC1A4 |  |  |
|  | SCARNA5 |  |  |
|  | ARMC9 |  |  |
|  | ARRB2 |  |  |
|  | MYOM2 |  |  |
|  | EN1 |  |  |
|  | LOC126860438 |  |  |
|  | BCOR |  |  |
|  | CD5 |  |  |
|  | SLC2A4 |  |  |
|  | B4GALT1 |  |  |
|  | TOE1 |  |  |
|  | TUBA1B |  |  |
|  | RETREG1 |  |  |
|  | SLC52A3 |  |  |
|  | IFT122 |  |  |
|  | FHL2 |  |  |
|  | RAB1A |  |  |
|  | MYH2 |  |  |
|  | PFN1 |  |  |
|  | CERS1 |  |  |
|  | TRH |  |  |
|  | NEGR1 |  |  |
|  | SIRT6 |  |  |
|  | CD1D |  |  |
|  | SLC5A2 |  |  |
|  | ALOX12 |  |  |
|  | HEATR3 |  |  |
|  | ITIH4 |  |  |
|  | NUDT15 |  |  |
|  | FGF14 |  |  |
|  | POT1 |  |  |
|  | RPL4 |  |  |
|  | USP9X |  |  |
|  | VEGFD |  |  |
|  | HAR1B |  |  |
|  | ITGAV |  |  |
|  | ALG2 |  |  |
|  | CNR2 |  |  |
|  | HRH1 |  |  |
|  | BRD2 |  |  |
|  | MIR99A |  |  |
|  | TRAF3IP1 |  |  |
|  | LPAR1 |  |  |
|  | AKR1A1 |  |  |
|  | ADIPOR1 |  |  |
|  | NR2F2 |  |  |
|  | EIF3K |  |  |
|  | EPHA2 |  |  |
|  | KIAA0319L |  |  |
|  | SETD1B |  |  |
|  | CRBN |  |  |
|  | HOOK1 |  |  |
|  | RAB11A |  |  |
|  | TUBB6 |  |  |
|  | LOC129936434 |  |  |
|  | NELFA |  |  |
|  | KIDINS220 |  |  |
|  | THOC6 |  |  |
|  | MIR499A |  |  |
|  | TMPRSS11D |  |  |
|  | ADAT3 |  |  |
|  | GLRX |  |  |
|  | ZFP57 |  |  |
|  | GTF3A |  |  |
|  | SLC6A14 |  |  |
|  | MSN |  |  |
|  | ILK |  |  |
|  | HES7 |  |  |
|  | HPS6 |  |  |
|  | DIO2 |  |  |
|  | CFHR3 |  |  |
|  | DNAH14 |  |  |
|  | DDR2 |  |  |
|  | DEFB103B |  |  |
|  | CHRM2 |  |  |
|  | LCN1 |  |  |
|  | SNORD115-1 |  |  |
|  | MCCC1 |  |  |
|  | MIR203A |  |  |
|  | FZD2 |  |  |
|  | IL13RA2 |  |  |
|  | EPCAM |  |  |
|  | SULT2B1 |  |  |
|  | BICC1 |  |  |
|  | PNKD |  |  |
|  | HSPA2 |  |  |
|  | C3AR1 |  |  |
|  | SYT2 |  |  |
|  | WASL |  |  |
|  | LOC113664106 |  |  |
|  | DAXX |  |  |
|  | H3-3B |  |  |
|  | SARM1 |  |  |
|  | DHTKD1 |  |  |
|  | PPP1R21 |  |  |
|  | KDM2B |  |  |
|  | PTCHD1 |  |  |
|  | MIR181C |  |  |
|  | MIRLET7A1 |  |  |
|  | UFD1 |  |  |
|  | CYP7A1 |  |  |
|  | GFM2 |  |  |
|  | EFNA5 |  |  |
|  | CD1E |  |  |
|  | PAX4 |  |  |
|  | DNAAF3 |  |  |
|  | NUP98 |  |  |
|  | HSP90AB1 |  |  |
|  | EDARADD |  |  |
|  | MIR27B |  |  |
|  | PRDM16 |  |  |
|  | LTBR |  |  |
|  | UNC93B1 |  |  |
|  | IL25 |  |  |
|  | TCN2 |  |  |
|  | AP2M1 |  |  |
|  | FPR2 |  |  |
|  | FBXO28 |  |  |
|  | COG7 |  |  |
|  | ATP5F1A |  |  |
|  | ERV3-1 |  |  |
|  | CDH17 |  |  |
|  | MLKL |  |  |
|  | SIN3A |  |  |
|  | DDRGK1 |  |  |
|  | ECE1 |  |  |
|  | RAB1B |  |  |
|  | CALB1 |  |  |
|  | ATG7 |  |  |
|  | OAT |  |  |
|  | IL31 |  |  |
|  | TRIM9 |  |  |
|  | RSPH4A |  |  |
|  | CS |  |  |
|  | CHD4 |  |  |
|  | MIR181A2 |  |  |
|  | TP73 |  |  |
|  | RALGAPA1 |  |  |
|  | MTX2 |  |  |
|  | MCM10 |  |  |
|  | AQP2 |  |  |
|  | OTULIN |  |  |
|  | KISS1 |  |  |
|  | PSMA4 |  |  |
|  | SOAT1 |  |  |
|  | TLN1 |  |  |
|  | LOC111674477 |  |  |
|  | TRPA1 |  |  |
|  | TIMMDC1 |  |  |
|  | HS6ST2 |  |  |
|  | RASGRP2 |  |  |
|  | ERBB4 |  |  |
|  | TKT |  |  |
|  | LETM1 |  |  |
|  | MIR511 |  |  |
|  | KCNJ5 |  |  |
|  | UBE2L3 |  |  |
|  | FADD |  |  |
|  | FGF5 |  |  |
|  | RNF113A |  |  |
|  | MID2 |  |  |
|  | APAF1 |  |  |
|  | LOC112997540 |  |  |
|  | SLC4A4 |  |  |
|  | BMPR1A |  |  |
|  | ETS1 |  |  |
|  | IFNL1 |  |  |
|  | SLC6A20 |  |  |
|  | ACO1 |  |  |
|  | DUOX1 |  |  |
|  | PRDM1 |  |  |
|  | SLC39A4 |  |  |
|  | MIR205 |  |  |
|  | SNORD44 |  |  |
|  | TMCO1 |  |  |
|  | LOC126859690 |  |  |
|  | PLEKHG2 |  |  |
|  | CC2D1A |  |  |
|  | ABO |  |  |
|  | PSMD3 |  |  |
|  | RNU6ATAC |  |  |
|  | RFX7 |  |  |
|  | DPYSL5 |  |  |
|  | PALS1 |  |  |
|  | NEPRO |  |  |
|  | LOC130059762 |  |  |
|  | BARD1 |  |  |
|  | PTGDS |  |  |
|  | GRB2 |  |  |
|  | IFNL2 |  |  |
|  | BST2 |  |  |
|  | SH2D3C |  |  |
|  | CYP21A1P |  |  |
|  | SPG21 |  |  |
|  | CD9 |  |  |
|  | FAM124A |  |  |
|  | NEDD4L |  |  |
|  | PTGES |  |  |
|  | FBXO7 |  |  |
|  | NCOR2 |  |  |
|  | WRAP53 |  |  |
|  | CACNA1E |  |  |
|  | NES |  |  |
|  | CNBP |  |  |
|  | EIF2B1 |  |  |
|  | MIR212 |  |  |
|  | ADAMTS7 |  |  |
|  | RIC1 |  |  |
|  | ROM1 |  |  |
|  | TUBGCP6 |  |  |
|  | COPS8 |  |  |
|  | EIF6 |  |  |
|  | NAT1 |  |  |
|  | DCK |  |  |
|  | RNF31 |  |  |
|  | NCAPD2 |  |  |
|  | HOXA2 |  |  |
|  | VASP |  |  |
|  | INPP5K |  |  |
|  | MYOCD |  |  |
|  | LOC106694315 |  |  |
|  | CRNDE |  |  |
|  | CAST |  |  |
|  | MYO9A |  |  |
|  | COL13A1 |  |  |
|  | RIT1 |  |  |
|  | COG6 |  |  |
|  | CD1C |  |  |
|  | RAB33B |  |  |
|  | PRMT1 |  |  |
|  | DRP2 |  |  |
|  | USP18 |  |  |
|  | POLD3 |  |  |
|  | KCNJ2 |  |  |
|  | ALX4 |  |  |
|  | ANKRD26 |  |  |
|  | CXCL16 |  |  |
|  | CD248 |  |  |
|  | SLC18A2 |  |  |
|  | KLHL3 |  |  |
|  | RAB6A |  |  |
|  | STX5 |  |  |
|  | CEBPE |  |  |
|  | ARL13B |  |  |
|  | FGF16 |  |  |
|  | ASAH2 |  |  |
|  | MCIDAS |  |  |
|  | CD1B |  |  |
|  | MATR3 |  |  |
|  | GDAP1L1 |  |  |
|  | QRSL1 |  |  |
|  | ITGAX |  |  |
|  | RNF216 |  |  |
|  | TPP2 |  |  |
|  | UQCRFS1 |  |  |
|  | MIR410 |  |  |
|  | TNFRSF18 |  |  |
|  | TMEM70 |  |  |
|  | FANCM |  |  |
|  | IL1F10 |  |  |
|  | MAPKAPK5 |  |  |
|  | RING1 |  |  |
|  | RAD51D |  |  |
|  | PRPF19 |  |  |
|  | COX6A1 |  |  |
|  | AOC1 |  |  |
|  | GSK3A |  |  |
|  | HSPB3 |  |  |
|  | TGM2 |  |  |
|  | PDE4B |  |  |
|  | FPR1 |  |  |
|  | FGF22 |  |  |
|  | LGALS1 |  |  |
|  | APOD |  |  |
|  | EIF2S1 |  |  |
|  | OTUD7A |  |  |
|  | PSMD4 |  |  |
|  | ACADSB |  |  |
|  | CA4 |  |  |
|  | MGLL |  |  |
|  | LGR5 |  |  |
|  | AOC3 |  |  |
|  | RAD51C |  |  |
|  | MARS2 |  |  |
|  | SLC6A19 |  |  |
|  | MFGE8 |  |  |
|  | LTA4H |  |  |
|  | FOXO3 |  |  |
|  | CARS1 |  |  |
|  | MINPP1 |  |  |
|  | JPH3 |  |  |
|  | TYRP1 |  |  |
|  | MIR155HG |  |  |
|  | PIGM |  |  |
|  | CAPN1 |  |  |
|  | PRKAR1B |  |  |
|  | SUPT5H |  |  |
|  | CRELD1 |  |  |
|  | BMX |  |  |
|  | MIR373 |  |  |
|  | ICAM3 |  |  |
|  | SLC38A8 |  |  |
|  | TOMM40 |  |  |
|  | CABIN1 |  |  |
|  | CLIC1 |  |  |
|  | IL36G |  |  |
|  | MIR216A |  |  |
|  | ACTL6A |  |  |
|  | SORL1 |  |  |
|  | ERLIN1 |  |  |
|  | HOTTIP |  |  |
|  | SCN4B |  |  |
|  | AGO2 |  |  |
|  | PSMC4 |  |  |
|  | GANAB |  |  |
|  | PITRM1 |  |  |
|  | CDCA7L |  |  |
|  | RBX1 |  |  |
|  | ACTN4 |  |  |
|  | MVP |  |  |
|  | ANXA1 |  |  |
|  | BORCS5 |  |  |
|  | PCCA |  |  |
|  | TXNL4A |  |  |
|  | TYSND1 |  |  |
|  | TUBA4A |  |  |
|  | SON |  |  |
|  | SEMA3A |  |  |
|  | TNFSF14 |  |  |
|  | ATF2 |  |  |
|  | FCN1 |  |  |
|  | VAPA |  |  |
|  | MPI |  |  |
|  | CDA |  |  |
|  | POLI |  |  |
|  | DDOST |  |  |
|  | KPNA1 |  |  |
|  | PHKG2 |  |  |
|  | WASF1 |  |  |
|  | GNLY |  |  |
|  | CLEC4A |  |  |
|  | HSPE1 |  |  |
|  | DIS3 |  |  |
|  | XRCC3 |  |  |
|  | NAE1 |  |  |
|  | RUSC2 |  |  |
|  | FCRL4 |  |  |
|  | FLI1 |  |  |
|  | SLC26A3 |  |  |
|  | SCLT1 |  |  |
|  | WAC |  |  |
|  | C4BPA |  |  |
|  | MTMR14 |  |  |
|  | CCDC78 |  |  |
|  | NUMA1 |  |  |
|  | ERVFRD-1 |  |  |
|  | MBTPS1 |  |  |
|  | MCM4 |  |  |
|  | LY75 |  |  |
|  | DNAJC19 |  |  |
|  | RPS6KA5 |  |  |
|  | DICER1 |  |  |
|  | P4HA2 |  |  |
|  | ALK |  |  |
|  | CFAP92 |  |  |
|  | CTRL |  |  |
|  | IFT52 |  |  |
|  | ALDH4A1 |  |  |
|  | RECQL |  |  |
|  | GRIN2D |  |  |
|  | DDB1 |  |  |
|  | DNAI1 |  |  |
|  | ATL3 |  |  |
|  | AFG2B |  |  |
|  | CYLD-AS2 |  |  |
|  | DLEU1 |  |  |
|  | ARHGAP6 |  |  |
|  | VIPR1 |  |  |
|  | LRP1B |  |  |
|  | ZNF668 |  |  |
|  | DALRD3 |  |  |
|  | PCAT2 |  |  |
|  | DVL3 |  |  |
|  | PSMD2 |  |  |
|  | CD33 |  |  |
|  | TRIM37 |  |  |
|  | DNAJC3 |  |  |
|  | TMEM165 |  |  |
|  | LOC105371046 |  |  |
|  | SEPT5-GP1BB |  |  |
|  | PAPOLG |  |  |
|  | ANKH |  |  |
|  | A2ML1 |  |  |
|  | SPEF2 |  |  |
|  | MIR124-1 |  |  |
|  | PRDX5 |  |  |
|  | STT3A |  |  |
|  | FNDC5 |  |  |
|  | MAN2C1 |  |  |
|  | P2RY1 |  |  |
|  | CPSF4 |  |  |
|  | CRK |  |  |
|  | HPS4 |  |  |
|  | BACE1-AS |  |  |
|  | TRAIP |  |  |
|  | PRKCZ |  |  |
|  | CARD10 |  |  |
|  | TNFRSF6B |  |  |
|  | VPS35L |  |  |
|  | MRAP2 |  |  |
|  | ZFYVE27 |  |  |
|  | TRL-TAG1-1 |  |  |
|  | HTATIP2 |  |  |
|  | FABP1 |  |  |
|  | SPAG17 |  |  |
|  | CWC27 |  |  |
|  | SLC26A9 |  |  |
|  | IVD |  |  |
|  | RFC1 |  |  |
|  | SNCB |  |  |
|  | PPP2CA |  |  |
|  | EXOSC2 |  |  |
|  | CLEC1A |  |  |
|  | MIR324 |  |  |
|  | CFAP70 |  |  |
|  | PRKCQ |  |  |
|  | PYCARD |  |  |
|  | SNCG |  |  |
|  | MTMR10 |  |  |
|  | PSMD1 |  |  |
|  | LOC112694756 |  |  |
|  | MT-RNR2 |  |  |
|  | RDX |  |  |
|  | BECN1 |  |  |
|  | YWHAQ |  |  |
|  | CALM1 |  |  |
|  | LOC130059394 |  |  |
|  | MAP3K5 |  |  |
|  | SNX9 |  |  |
|  | IFT27 |  |  |
|  | DCLRE1B |  |  |
|  | TNIP1 |  |  |
|  | UQCRC1 |  |  |
|  | LIPE |  |  |
|  | CCHCR1 |  |  |
|  | ARCN1 |  |  |
|  | NUDC |  |  |
|  | TNPO1 |  |  |
|  | MIR298 |  |  |
|  | MTHFS |  |  |
|  | CPB2 |  |  |
|  | GPR35 |  |  |
|  | UGT2B7 |  |  |
|  | ST2 |  |  |
|  | NMT1 |  |  |
|  | NAA11 |  |  |
|  | AP1S1 |  |  |
|  | PHF21A |  |  |
|  | DNAJC30 |  |  |
|  | USP8 |  |  |
|  | PMPCB |  |  |
|  | ELP6 |  |  |
|  | PSMA3 |  |  |
|  | WIPI2 |  |  |
|  | KIF20A |  |  |
|  | PDHB |  |  |
|  | DZIP1 |  |  |
|  | CEACAM6 |  |  |
|  | DHFR2 |  |  |
|  | PPCS |  |  |
|  | PTK2B |  |  |
|  | MYT1L |  |  |
|  | APOBEC1 |  |  |
|  | SPN |  |  |
|  | ROCR |  |  |
|  | CD47 |  |  |
|  | AMACR |  |  |
|  | SMAD9 |  |  |
|  | RPL9 |  |  |
|  | CCNA2 |  |  |
|  | LOC126860933 |  |  |
|  | A2M |  |  |
|  | RIN2 |  |  |
|  | MIR532 |  |  |
|  | MIR124-3 |  |  |
|  | FIS1 |  |  |
|  | WDR5 |  |  |
|  | CD63 |  |  |
|  | MIR125B2 |  |  |
|  | MPIG6B |  |  |
|  | ELP2 |  |  |
|  | IL4I1 |  |  |
|  | LOC126863207 |  |  |
|  | RORC |  |  |
|  | REEP6 |  |  |
|  | PLCB3 |  |  |
|  | SERPINE2 |  |  |
|  | TBX2 |  |  |
|  | ADD1 |  |  |
|  | LIPT1 |  |  |
|  | ATF6B |  |  |
|  | HAR1A |  |  |
|  | MS4A2 |  |  |
|  | CAV2 |  |  |
|  | ATF4 |  |  |
|  | NME4 |  |  |
|  | CARTPT |  |  |
|  | DANCR |  |  |
|  | RNF125 |  |  |
|  | PPARD |  |  |
|  | SMC6 |  |  |
|  | USP14 |  |  |
|  | XYLT2 |  |  |
|  | FGL2 |  |  |
|  | CORIN |  |  |
|  | UBQLN2 |  |  |
|  | CSK |  |  |
|  | SLC25A6 |  |  |
|  | ERVK-6 |  |  |
|  | SLC9A7 |  |  |
|  | CASC9 |  |  |
|  | KCNC3 |  |  |
|  | KCNN4 |  |  |
|  | PSMA5 |  |  |
|  | MIR18A |  |  |
|  | ZNF622 |  |  |
|  | AHSP |  |  |
|  | PSMD11 |  |  |
|  | ARHGEF1 |  |  |
|  | TAT |  |  |
|  | NOL10 |  |  |
|  | OMP |  |  |
|  | VDAC2 |  |  |
|  | ODAD3 |  |  |
|  | IARS1 |  |  |
|  | TRAK1 |  |  |
|  | PSMA2 |  |  |
|  | LRIG2 |  |  |
|  | LINC01772 |  |  |
|  | ALDH9A1 |  |  |
|  | BLZF1 |  |  |
|  | CTRC |  |  |
|  | CDKN2C |  |  |
|  | METTL3 |  |  |
|  | CASP10 |  |  |
|  | CRB2 |  |  |
|  | PSMC5 |  |  |
|  | MIR1236 |  |  |
|  | OXR1 |  |  |
|  | UPK3A |  |  |
|  | CTSS |  |  |
|  | DEFA3 |  |  |
|  | DUSP13B |  |  |
|  | EXO1 |  |  |
|  | STOM |  |  |
|  | BAZ1B |  |  |
|  | RAD9A |  |  |
|  | ALDH1A2 |  |  |
|  | CLEC4E |  |  |
|  | TRN-GTT2-1 |  |  |
|  | TNFRSF4 |  |  |
|  | TNXA |  |  |
|  | MIR92A1 |  |  |
|  | RNMT |  |  |
|  | GIP |  |  |
|  | ARL2BP |  |  |
|  | CFC1 |  |  |
|  | YPEL3 |  |  |
|  | STX17 |  |  |
|  | GRIA2 |  |  |
|  | TET2-AS1 |  |  |
|  | RAD52 |  |  |
|  | SLC7A7 |  |  |
|  | CARD8 |  |  |
|  | SP3 |  |  |
|  | LY75-CD302 |  |  |
|  | DCAF17 |  |  |
|  | CLIP1 |  |  |
|  | ANXA4 |  |  |
|  | NANOG |  |  |
|  | IGHJ1 |  |  |
|  | HCK |  |  |
|  | PTPN5 |  |  |
|  | JARID2 |  |  |
|  | NPY2R |  |  |
|  | PDE10A |  |  |
|  | CTTN |  |  |
|  | PSMC2 |  |  |
|  | PRSS8 |  |  |
|  | TRPM8 |  |  |
|  | CHRNB3 |  |  |
|  | GAS6 |  |  |
|  | NDUFC2 |  |  |
|  | NXN |  |  |
|  | COG1 |  |  |
|  | PLA2G10 |  |  |
|  | PLEK2 |  |  |
|  | EEA1 |  |  |
|  | PHKB |  |  |
|  | GLRA1 |  |  |
|  | SERPINA4 |  |  |
|  | KEAP1 |  |  |
|  | TAS2R38 |  |  |
|  | M6PR |  |  |
|  | AAK1 |  |  |
|  | KLRD1 |  |  |
|  | KBTBD13 |  |  |
|  | PDPN |  |  |
|  | GTF2H4 |  |  |
|  | FSCN2 |  |  |
|  | BPTF |  |  |
|  | RUVBL2 |  |  |
|  | NBR1 |  |  |
|  | PHLDB1 |  |  |
|  | ERAP1 |  |  |
|  | SERPINB2 |  |  |
|  | PSMA1 |  |  |
|  | SLAMF6 |  |  |
|  | MIP |  |  |
|  | MIR101-1 |  |  |
|  | GTF2I |  |  |
|  | KCTD1 |  |  |
|  | PTPRU |  |  |
|  | SLC30A7 |  |  |
|  | KPNB1 |  |  |
|  | VANGL1 |  |  |
|  | EPAS1 |  |  |
|  | RANBP9 |  |  |
|  | UBE2D1 |  |  |
|  | FCN3 |  |  |
|  | NOP2 |  |  |
|  | PRKRA |  |  |
|  | SEMA4D |  |  |
|  | PAX5 |  |  |
|  | AIC |  |  |
|  | SLC7A9 |  |  |
|  | RAB3A |  |  |
|  | LIFR |  |  |
|  | TRAC |  |  |
|  | WDR45B |  |  |
|  | TBL2 |  |  |
|  | PDS5A |  |  |
|  | PHOX2A |  |  |
|  | OGDH |  |  |
|  | PET117 |  |  |
|  | EGFL8 |  |  |
|  | ICAM2 |  |  |
|  | LOC126862447 |  |  |
|  | ASIC2 |  |  |
|  | GLRB |  |  |
|  | KRT71 |  |  |
|  | GPANK1 |  |  |
|  | SMOC1 |  |  |
|  | GDPD3 |  |  |
|  | MUC4 |  |  |
|  | CENATAC |  |  |
|  | RAB4B-EGLN2 |  |  |
|  | DYNLL1 |  |  |
|  | MIR182 |  |  |
|  | LTB4R |  |  |
|  | RBM28 |  |  |
|  | PPT2 |  |  |
|  | TRPV2 |  |  |
|  | PMP2 |  |  |
|  | DOCK11 |  |  |
|  | LOC126860330 |  |  |
|  | LOC110011216 |  |  |
|  | GYG2 |  |  |
|  | RSPH3 |  |  |
|  | CDX2 |  |  |
|  | MT2A |  |  |
|  | FKBP1A |  |  |
|  | SV2A |  |  |
|  | ATXN2L |  |  |
|  | NCK1 |  |  |
|  | PSMD12 |  |  |
|  | RPL3 |  |  |
|  | HARS2 |  |  |
|  | GTF2IRD2 |  |  |
|  | FUT3 |  |  |
|  | FLOT1 |  |  |
|  | CD68 |  |  |
|  | DROSHA |  |  |
|  | HLA-DQA2 |  |  |
|  | SOX3 |  |  |
|  | FLNC-AS1 |  |  |
|  | TOR1AIP1 |  |  |
|  | LPO |  |  |
|  | SLC19A2 |  |  |
|  | FCRL5 |  |  |
|  | SPTBN4 |  |  |
|  | RAB39B |  |  |
|  | POLRMT |  |  |
|  | EIF2AK4 |  |  |
|  | NME1 |  |  |
|  | SMARCC1 |  |  |
|  | STH |  |  |
|  | LOC130068460 |  |  |
|  | SSUH2 |  |  |
|  | WNK4 |  |  |
|  | DNAJB1 |  |  |
|  | LOC130059663 |  |  |
|  | SIGLEC1 |  |  |
|  | TNFSF13 |  |  |
|  | DTWD2 |  |  |
|  | RPL3L |  |  |
|  | SLX4 |  |  |
|  | COPS7A |  |  |
|  | DGCR6 |  |  |
|  | MRC2 |  |  |
|  | MIR26A1 |  |  |
|  | CCND3 |  |  |
|  | DMBT1 |  |  |
|  | OXA1L |  |  |
|  | MYMK |  |  |
|  | MAP4K4 |  |  |
|  | RNF217-AS1 |  |  |
|  | MCHR1 |  |  |
|  | ARG2 |  |  |
|  | TXK |  |  |
|  | PTGDR2 |  |  |
|  | GJB4 |  |  |
|  | LARP1 |  |  |
|  | PRRC2A |  |  |
|  | LOC130068308 |  |  |
|  | CCR9 |  |  |
|  | MRAP |  |  |
|  | SERPINB5 |  |  |
|  | LOC130060113 |  |  |
|  | P2RY8 |  |  |
|  | SELENOT |  |  |
|  | G3BP1 |  |  |
|  | IDE |  |  |
|  | SAPCD1 |  |  |
|  | DNAAF11 |  |  |
|  | ITGA5 |  |  |
|  | ADORA1 |  |  |
|  | USP13 |  |  |
|  | XPNPEP2 |  |  |
|  | WNT9B |  |  |
|  | CD82 |  |  |
|  | MAPKBP1 |  |  |
|  | MYO18A |  |  |
|  | PMCH |  |  |
|  | PIP4K2C |  |  |
|  | PPM1D |  |  |
|  | CSNK1E |  |  |
|  | PATJ |  |  |
|  | TERF2 |  |  |
|  | H4C5 |  |  |
|  | FUT8 |  |  |
|  | THAP1 |  |  |
|  | SNAP91 |  |  |
|  | APOC1 |  |  |
|  | EIF3C |  |  |
|  | TNFRSF10A |  |  |
|  | MDFIC |  |  |
|  | TMEM106B |  |  |
|  | VLDLR |  |  |
|  | SNORD116-1 |  |  |
|  | PCGF2 |  |  |
|  | SERPINB4 |  |  |
|  | GRHPR |  |  |
|  | AIM2 |  |  |
|  | CIRBP |  |  |
|  | MAPKAPK2 |  |  |
|  | LOC130005193 |  |  |
|  | LAIR1 |  |  |
|  | BTLA |  |  |
|  | SNIP1 |  |  |
|  | BAG6 |  |  |
|  | ACACA |  |  |
|  | SMG9 |  |  |
|  | RTN4 |  |  |
|  | ELMOD2 |  |  |
|  | TMTC3 |  |  |
|  | SNORD80 |  |  |
|  | PYROXD1 |  |  |
|  | SHROOM4 |  |  |
|  | CASC15 |  |  |
|  | VSIG4 |  |  |
|  | MECR |  |  |
|  | NGLY1 |  |  |
|  | H2AC20 |  |  |
|  | CAPN5 |  |  |
|  | KIF2A |  |  |
|  | BAK1 |  |  |
|  | HPX |  |  |
|  | ANKK1 |  |  |
|  | KIFBP |  |  |
|  | ADH5 |  |  |
|  | ID1 |  |  |
|  | CELF1 |  |  |
|  | JAG2 |  |  |
|  | DNAAF4-CCPG1 |  |  |
|  | NNT |  |  |
|  | HMGN2 |  |  |
|  | TTC12 |  |  |
|  | CYB5B |  |  |
|  | LOC113633877 |  |  |
|  | GUF1 |  |  |
|  | CASP12 |  |  |
|  | TIMM22 |  |  |
|  | BHLHA9 |  |  |
|  | SULT1A1 |  |  |
|  | BIRC2 |  |  |
|  | APBB3 |  |  |
|  | MIR16-2 |  |  |
|  | HPS3 |  |  |
|  | GPRASP2 |  |  |
|  | ATG14 |  |  |
|  | COPS4 |  |  |
|  | ADAMTS1 |  |  |
|  | CAMLG |  |  |
|  | MOV10 |  |  |
|  | SEPTIN2 |  |  |
|  | ARF6 |  |  |
|  | RHOF |  |  |
|  | TOP3A |  |  |
|  | TRPM7 |  |  |
|  | KCNE2 |  |  |
|  | ISL1 |  |  |
|  | SREBF2 |  |  |
|  | SIK1 |  |  |
|  | PSMD6 |  |  |
|  | KIF3B |  |  |
|  | PVR |  |  |
|  | FDX2 |  |  |
|  | PAXX |  |  |
|  | EIF3B |  |  |
|  | TXN2 |  |  |
|  | ATF3 |  |  |
|  | B3GAT1 |  |  |
|  | IL15RA |  |  |
|  | NKILA |  |  |
|  | UVSSA |  |  |
|  | PDE4DIP |  |  |
|  | CLCC1 |  |  |
|  | TPCN2 |  |  |
|  | NME8 |  |  |
|  | BCL7B |  |  |
|  | CEACAM5 |  |  |
|  | UBA2 |  |  |
|  | EXOSC5 |  |  |
|  | TRIM33 |  |  |
|  | DLAT |  |  |
|  | TCN1 |  |  |
|  | MFN1 |  |  |
|  | ANPEP |  |  |
|  | POM121L12 |  |  |
|  | MIR103B2 |  |  |
|  | TH2LCRR |  |  |
|  | ATP6AP2 |  |  |
|  | EIF3L |  |  |
|  | CLCA4 |  |  |
|  | SDK1 |  |  |
|  | CEP19 |  |  |
|  | IL1RAP |  |  |
|  | MKLN1 |  |  |
|  | MARCO |  |  |
|  | NRGN |  |  |
|  | F2R |  |  |
|  | LALBA |  |  |
|  | UFC1 |  |  |
|  | NAXE |  |  |
|  | ANP32B |  |  |
|  | TARS2 |  |  |
|  | TNFSF15 |  |  |
|  | RTL1 |  |  |
|  | RXRB |  |  |
|  | CEP89 |  |  |
|  | CELF2 |  |  |
|  | SDR9C7 |  |  |
|  | TSEN15 |  |  |
|  | SLF2 |  |  |
|  | TPPP |  |  |
|  | VANGL2 |  |  |
|  | TAF13 |  |  |
|  | SLC2A3 |  |  |
|  | GFER |  |  |
|  | CITED2 |  |  |
|  | LRP8 |  |  |
|  | KIF15 |  |  |
|  | BAD |  |  |
|  | NEMF |  |  |
|  | RAB11B |  |  |
|  | RPS15 |  |  |
|  | NFATC1 |  |  |
|  | FOXI3 |  |  |
|  | CCL8 |  |  |
|  | EIF3A |  |  |
|  | SMPX |  |  |
|  | KANSL3 |  |  |
|  | DNASE1L3 |  |  |
|  | PRG2 |  |  |
|  | LY6G5B |  |  |
|  | TNFRSF17 |  |  |
|  | TRIM63 |  |  |
|  | WTIP |  |  |
|  | MGME1 |  |  |
|  | LRP6 |  |  |
|  | IFIT1 |  |  |
|  | DCTN2 |  |  |
|  | EHMT2 |  |  |
|  | COPG1 |  |  |
|  | BDKRB1 |  |  |
|  | MIR497 |  |  |
|  | HSPA14 |  |  |
|  | BRWD1 |  |  |
|  | PTPA |  |  |
|  | HPR |  |  |
|  | SNHG3 |  |  |
|  | CACNA1C-AS1 |  |  |
|  | FTSJ1 |  |  |
|  | DEFB103A |  |  |
|  | RAB4A |  |  |
|  | DHX16 |  |  |
|  | VWA7 |  |  |
|  | SEC22B |  |  |
|  | GRK3 |  |  |
|  | PES1 |  |  |
|  | MIRLET7F1 |  |  |
|  | FCGRT |  |  |
|  | DAB1 |  |  |
|  | CYLD-AS1 |  |  |
|  | LOC129993794 |  |  |
|  | AMY2A |  |  |
|  | GNB2 |  |  |
|  | MIR31HG |  |  |
|  | LOC109504728 |  |  |
|  | PPY |  |  |
|  | SIGLEC7 |  |  |
|  | MBNL2 |  |  |
|  | MIR455 |  |  |
|  | NPSR1 |  |  |
|  | LATS1 |  |  |
|  | NUP62 |  |  |
|  | GTF2E2 |  |  |
|  | RAN |  |  |
|  | LOC126859961 |  |  |
|  | LOC126859962 |  |  |
|  | LOC130055542 |  |  |
|  | GJA3 |  |  |
|  | AQP1 |  |  |
|  | PNLIP |  |  |
|  | BCKDK |  |  |
|  | FMO1 |  |  |
|  | PGD |  |  |
|  | EIF3M |  |  |
|  | LIPN |  |  |
|  | PMM1 |  |  |
|  | PNPLA8 |  |  |
|  | ARHGAP45 |  |  |
|  | CRACR2A |  |  |
|  | NIPA2 |  |  |
|  | PIK3R4 |  |  |
|  | RNU6-1 |  |  |
|  | YPEL3-DT |  |  |
|  | CORO1A-AS1 |  |  |
|  | LOC125146441 |  |  |
|  | LOC130058808 |  |  |
|  | LOC130058809 |  |  |
|  | LOC130058810 |  |  |
|  | LOC130058811 |  |  |
|  | LOC130058812 |  |  |
|  | LOC130058813 |  |  |
|  | LOC130058814 |  |  |
|  | LOC130058815 |  |  |
|  | LOC130058816 |  |  |
|  | LOC130058817 |  |  |
|  | LOC130058818 |  |  |
|  | LOC130058819 |  |  |
|  | UBAP2L |  |  |
|  | MVD |  |  |
|  | FERMT2 |  |  |
|  | MAPK11 |  |  |
|  | KLRC1 |  |  |
|  | MDK |  |  |
|  | ITPKB |  |  |
|  | LOC130060903 |  |  |
|  | CHD6 |  |  |
|  | SUN2 |  |  |
|  | MNX1 |  |  |
|  | CRHBP |  |  |
|  | HIC1 |  |  |
|  | TRAF3IP2 |  |  |
|  | PXN |  |  |
|  | CCR6 |  |  |
|  | PRDM8 |  |  |
|  | HCP5 |  |  |
|  | MIR1246 |  |  |
|  | ULK1 |  |  |
|  | MRTFA |  |  |
|  | GUCY2C |  |  |
|  | PRKCSH |  |  |
|  | NRP2 |  |  |
|  | SNAPIN |  |  |
|  | NHERF1 |  |  |
|  | MIR1260B |  |  |
|  | CRLS1 |  |  |
|  | SSR3 |  |  |
|  | ARSD |  |  |
|  | CTSF |  |  |
|  | SEC63 |  |  |
|  | PSMB5 |  |  |
|  | H4C1 |  |  |
|  | CCT5 |  |  |
|  | DGCR8 |  |  |
|  | HCLS1 |  |  |
|  | SNX8 |  |  |
|  | H2AC17 |  |  |
|  | SLC6A15 |  |  |
|  | LOC130003417 |  |  |
|  | CD58 |  |  |
|  | ZNF699 |  |  |
|  | H2AC16 |  |  |
|  | PRKAA1 |  |  |
|  | HCG27 |  |  |
|  | GPR12 |  |  |
|  | HSPB2 |  |  |
|  | MIR503 |  |  |
|  | SELENOS |  |  |
|  | MAPK9 |  |  |
|  | H3-4 |  |  |
|  | DSC3 |  |  |
|  | HAGLROS |  |  |
|  | ATP2A3 |  |  |
|  | ATP2A1 |  |  |
|  | CCL16 |  |  |
|  | LOC126863158 |  |  |
|  | MGAT2 |  |  |
|  | TOM1 |  |  |
|  | CUL1 |  |  |
|  | MTMR12 |  |  |
|  | AMMECR1 |  |  |
|  | FAM50A |  |  |
|  | RASSF5 |  |  |
|  | RO60 |  |  |
|  | ATP5MG |  |  |
|  | HMSD |  |  |
|  | PSIP1 |  |  |
|  | RBM24 |  |  |
|  | MACROD2 |  |  |
|  | RNF5 |  |  |
|  | SECISBP2 |  |  |
|  | EIF3I |  |  |
|  | VAX1 |  |  |
|  | FSCN1 |  |  |
|  | TRAF5 |  |  |
|  | C8B |  |  |
|  | COPS6 |  |  |
|  | C8A |  |  |
|  | MARVELD3 |  |  |
|  | MTAP |  |  |
|  | LOC126859827 |  |  |
|  | CRYAA |  |  |
|  | DNAJA1 |  |  |
|  | POU3F4 |  |  |
|  | RSPH9 |  |  |
|  | MIR326 |  |  |
|  | FEN1 |  |  |
|  | DVL2 |  |  |
|  | YWHAZ |  |  |
|  | BCL2A1 |  |  |
|  | H3C12 |  |  |
|  | ERGIC1 |  |  |
|  | LINC01080 |  |  |
|  | LRP1-AS |  |  |
|  | SORL1-AS1 |  |  |
|  | LINC01616 |  |  |
|  | NECTIN1 |  |  |
|  | ITM2B |  |  |
|  | RPS3 |  |  |
|  | PCSK6 |  |  |
|  | KLRC4 |  |  |
|  | ANO3 |  |  |
|  | CSNK1A1 |  |  |
|  | DUSP19 |  |  |
|  | METAP2 |  |  |
|  | CLEC11A |  |  |
|  | MEAF6 |  |  |
|  | PDP1 |  |  |
|  | GINS1 |  |  |
|  | IGFBP4 |  |  |
|  | MYSM1 |  |  |
|  | MIB1 |  |  |
|  | APEH |  |  |
|  | CYSLTR2 |  |  |
|  | PIM1 |  |  |
|  | CCDC120 |  |  |
|  | CHRM1 |  |  |
|  | ZDHHC8 |  |  |
|  | TBC1D4 |  |  |
|  | MRI1 |  |  |
|  | CCR10 |  |  |
|  | SCAP |  |  |
|  | XRCC2 |  |  |
|  | PRDX1 |  |  |
|  | MCAM |  |  |
|  | DPH1 |  |  |
|  | SLC4A5 |  |  |
|  | PTMA |  |  |
|  | CISD2 |  |  |
|  | TMLHE |  |  |
|  | NOX1 |  |  |
|  | DELEC1 |  |  |
|  | FAM135A |  |  |
|  | SCARF1 |  |  |
|  | TAF2 |  |  |
|  | TBX22 |  |  |
|  | CCKBR |  |  |
|  | SLC22A2 |  |  |
|  | KSR2 |  |  |
|  | KPNA6 |  |  |
|  | AP3S1 |  |  |
|  | CALML5 |  |  |
|  | STAP2 |  |  |
|  | MIR103A1 |  |  |
|  | SPIB |  |  |
|  | PITX3 |  |  |
|  | GTF2H1 |  |  |
|  | EOLA1-DT |  |  |
|  | EPHX2 |  |  |
|  | PAGR1 |  |  |
|  | MIR184 |  |  |
|  | GIPC2 |  |  |
|  | COG3 |  |  |
|  | LOC132090498 |  |  |
|  | STMN2 |  |  |
|  | RENBP |  |  |
|  | CCDC47 |  |  |
|  | LOC112679198 |  |  |
|  | NEK10 |  |  |
|  | PLAC4 |  |  |
|  | SUGT1 |  |  |
|  | ELOC |  |  |
|  | C6orf15 |  |  |
|  | PPP1R2 |  |  |
|  | LNX1 |  |  |
|  | ADORA2B |  |  |
|  | BCL2L2-PABPN1 |  |  |
|  | DHX8 |  |  |
|  | GARIN5A |  |  |
|  | SNRPD3 |  |  |
|  | SORT1 |  |  |
|  | TAOK1 |  |  |
|  | NCR1 |  |  |
|  | SIGLEC5 |  |  |
|  | REV1 |  |  |
|  | H3C13 |  |  |
|  | MAP2K7 |  |  |
|  | FST |  |  |
|  | UMODL1 |  |  |
|  | MTMR1 |  |  |
|  | LOC130006336 |  |  |
|  | TUBGCP2 |  |  |
|  | AKAP8L |  |  |
|  | ERG |  |  |
|  | SLC8A2 |  |  |
|  | ERN1 |  |  |
|  | CEP350 |  |  |
|  | NOX3 |  |  |
|  | KMT2E |  |  |
|  | BAIAP2 |  |  |
|  | PRSS57 |  |  |
|  | ACY1 |  |  |
|  | GPLD1 |  |  |
|  | PSMB2 |  |  |
|  | GSTO1 |  |  |
|  | MST1 |  |  |
|  | LSG1 |  |  |
|  | TSPAN32 |  |  |
|  | CASP4 |  |  |
|  | RFC2 |  |  |
|  | LOC654780 |  |  |
|  | ANKLE1 |  |  |
|  | TSPYL1 |  |  |
|  | CEP43 |  |  |
|  | EP300-AS1 |  |  |
|  | MIR545 |  |  |
|  | ELF4 |  |  |
|  | EIF3D |  |  |
|  | ITGA1 |  |  |
|  | TNNI1 |  |  |
|  | BACH2 |  |  |
|  | ERCC6L2 |  |  |
|  | ZC3HC1 |  |  |
|  | ANKS6 |  |  |
|  | TMC8 |  |  |
|  | SASH3 |  |  |
|  | SLC18A3 |  |  |
|  | IMPDH2 |  |  |
|  | PCA3 |  |  |
|  | ESS2 |  |  |
|  | RHOT1 |  |  |
|  | CDC42EP3 |  |  |
|  | MAEA |  |  |
|  | TPK1 |  |  |
|  | DCTN4 |  |  |
|  | LAX1 |  |  |
|  | EIF3H |  |  |
|  | ABT1 |  |  |
|  | H3-7 |  |  |
|  | STK26 |  |  |
|  | PIK3C3 |  |  |
|  | P2RY2 |  |  |
|  | CBFA2T2 |  |  |
|  | PLA2G5 |  |  |
|  | MAP3K1 |  |  |
|  | USP48 |  |  |
|  | MLXIPL |  |  |
|  | GAP43 |  |  |
|  | H3-5 |  |  |
|  | DNAJB11 |  |  |
|  | PAK4 |  |  |
|  | VAMP7 |  |  |
|  | CLDN5 |  |  |
|  | GTF2H2 |  |  |
|  | PGRMC1 |  |  |
|  | FBXO11 |  |  |
|  | MTMR6 |  |  |
|  | PRPH |  |  |
|  | BICRA |  |  |
|  | E2F4 |  |  |
|  | SLC35A3 |  |  |
|  | GNAL |  |  |
|  | B3GALT4 |  |  |
|  | LAP3 |  |  |
|  | BRPF1 |  |  |
|  | PSMB3 |  |  |
|  | C7 |  |  |
|  | MYL9 |  |  |
|  | INSM1 |  |  |
|  | EOMES |  |  |
|  | CXCL6 |  |  |
|  | PNMT |  |  |
|  | DEF6 |  |  |
|  | CA9 |  |  |
|  | SELENOO |  |  |
|  | TUBA1C |  |  |
|  | RIPK3 |  |  |
|  | GRPR |  |  |
|  | CAPN10 |  |  |
|  | LILRB4 |  |  |
|  | DNAJC11 |  |  |
|  | LPCAT3 |  |  |
|  | VAV1 |  |  |
|  | TH2-LCR |  |  |
|  | AQP5-AS1 |  |  |
|  | MLYCD |  |  |
|  | NIPAL2 |  |  |
|  | CLCN3 |  |  |
|  | CAMKMT |  |  |
|  | DLG2 |  |  |
|  | SP100 |  |  |
|  | SP140 |  |  |
|  | RPAP3 |  |  |
|  | CACNG3 |  |  |
|  | AMY2B |  |  |
|  | DDX17 |  |  |
|  | TRV-AAC1-4 |  |  |
|  | EGLN1 |  |  |
|  | PHF8 |  |  |
|  | PIPOX |  |  |
|  | STX18 |  |  |
|  | CA8 |  |  |
|  | ECM1 |  |  |
|  | NIPAL3 |  |  |
|  | KPNA4 |  |  |
|  | GPR18 |  |  |
|  | PCDH8 |  |  |
|  | CES1 |  |  |
|  | LINC00707 |  |  |
|  | SELENOK |  |  |
|  | LINC01191 |  |  |
|  | ARRB1 |  |  |
|  | PYDC1 |  |  |
|  | GMDS |  |  |
|  | AMY1B |  |  |
|  | RNF17 |  |  |
|  | SLC7A8 |  |  |
|  | TOMM70 |  |  |
|  | CETN2 |  |  |
|  | SRSF1 |  |  |
|  | ING4 |  |  |
|  | ODAPH |  |  |
|  | TUBA3E |  |  |
|  | KCTD17 |  |  |
|  | CDHR3 |  |  |
|  | MTMR7 |  |  |
|  | TM2D3 |  |  |
|  | LPP |  |  |
|  | COMA |  |  |
|  | IL24 |  |  |
|  | CNTLN |  |  |
|  | ACER1 |  |  |
|  | SYNGR1 |  |  |
|  | ZBTB21 |  |  |
|  | POLA2 |  |  |
|  | MED27 |  |  |
|  | CLEC4D |  |  |
|  | PSMC6 |  |  |
|  | MBD4 |  |  |
|  | AKAP13 |  |  |
|  | DKK3 |  |  |
|  | TNFAIP8L2 |  |  |
|  | PAPPA-AS1 |  |  |
|  | DOCK1 |  |  |
|  | TMEM18 |  |  |
|  | HYCC1 |  |  |
|  | CENPO |  |  |
|  | CH25H |  |  |
|  | NKX2-6 |  |  |
|  | GTF3C1 |  |  |
|  | FOXJ1 |  |  |
|  | DLX4 |  |  |
|  | AAGAB |  |  |
|  | NMD3 |  |  |
|  | RSPH1 |  |  |
|  | TM2D1 |  |  |
|  | IL17RC |  |  |
|  | OR8J1 |  |  |
|  | TEAD1 |  |  |
|  | LOC123038178 |  |  |
|  | GJA5 |  |  |
|  | MTMR4 |  |  |
|  | MEAK7 |  |  |
|  | FMO5 |  |  |
|  | LOC129391064 |  |  |
|  | EIF4H |  |  |
|  | ILF2 |  |  |
|  | CFAP298 |  |  |
|  | VPS16 |  |  |
|  | TSPY1 |  |  |
|  | ZNF384 |  |  |
|  | STARD3NL |  |  |
|  | GORASP1 |  |  |
|  | MIR656 |  |  |
|  | PLEKHG4 |  |  |
|  | DNMT3L |  |  |
|  | MIR492 |  |  |
|  | GFOD3P |  |  |
|  | FUCA2 |  |  |
|  | HFM1 |  |  |
|  | TCF25 |  |  |
|  | DECR1 |  |  |
|  | GYS2 |  |  |
|  | MALRD1 |  |  |
|  | CLEC5A |  |  |
|  | SSTR2 |  |  |
|  | EXTL2 |  |  |
|  | C10orf90 |  |  |
|  | ZNF711 |  |  |
|  | MDCMP |  |  |
|  | SLC25A5 |  |  |
|  | UBQLN1 |  |  |
|  | TULP3 |  |  |
|  | POMP |  |  |
|  | DNMBP |  |  |
|  | CLEC4G |  |  |
|  | SLC44A4 |  |  |
|  | PTGER4 |  |  |
|  | UGGT1 |  |  |
|  | NTF4 |  |  |
|  | MICALL1 |  |  |
|  | MAP3K3 |  |  |
|  | CPSF3 |  |  |
|  | PPP2R2B |  |  |
|  | CFD |  |  |
|  | ARF3 |  |  |
|  | BPIFB1 |  |  |
|  | STK19 |  |  |
|  | EIF1AX |  |  |
|  | CASP7 |  |  |
|  | CTSH |  |  |
|  | GBX2 |  |  |
|  | FOXO1 |  |  |
|  | PPP1R10 |  |  |
|  | SNRPA |  |  |
|  | MED15 |  |  |
|  | TMEM240 |  |  |
|  | EPM2A-DT |  |  |
|  | HCG25 |  |  |
|  | DPM3 |  |  |
|  | S100A7 |  |  |
|  | BUD23 |  |  |
|  | LRRC7 |  |  |
|  | LDHB |  |  |
|  | EGFR-AS1 |  |  |
|  | ABCA7 |  |  |
|  | ADAM8 |  |  |
|  | LINC02972 |  |  |
|  | IFNA17 |  |  |
|  | PPP6R2 |  |  |
|  | HS2ST1 |  |  |
|  | VTI1B |  |  |
|  | GNA12 |  |  |
|  | DOCK3 |  |  |
|  | PYGL |  |  |
|  | MINAR2 |  |  |
|  | LOC129997381 |  |  |
|  | MTMR3 |  |  |
|  | GPR6 |  |  |
|  | TRIM62 |  |  |
|  | RUNX1T1 |  |  |
|  | PLXNB2 |  |  |
|  | TRMT9B |  |  |
|  | LOC106050103 |  |  |
|  | LONP2 |  |  |
|  | NCOA6 |  |  |
|  | UBE2N |  |  |
|  | SNX25 |  |  |
|  | TBRG1 |  |  |
|  | SIGLEC8 |  |  |
|  | APOBEC3F |  |  |
|  | CD83 |  |  |
|  | ASIC1 |  |  |
|  | NSMCE1 |  |  |
|  | LOC126805612 |  |  |
|  | RUNX3 |  |  |
|  | BTRC |  |  |
|  | MUC6 |  |  |
|  | LOC111674463 |  |  |
|  | CALML3 |  |  |
|  | CCRL2 |  |  |
|  | HRG |  |  |
|  | TNFSF9 |  |  |
|  | HLA-DQB2 |  |  |
|  | SCG2 |  |  |
|  | CSN3 |  |  |
|  | BMAL1 |  |  |
|  | SNORD24 |  |  |
|  | HSPH1 |  |  |
|  | SEC31A |  |  |
|  | ESCO1 |  |  |
|  | LAMP3 |  |  |
|  | PDIA3 |  |  |
|  | ALG9 |  |  |
|  | GK2 |  |  |
|  | UQCRH |  |  |
|  | RAB2A |  |  |
|  | KCNJ18 |  |  |
|  | ABCF1 |  |  |
|  | SKAP1-AS2 |  |  |
|  | THORLNC |  |  |
|  | GOLGB1 |  |  |
|  | RHOB |  |  |
|  | NAAA |  |  |
|  | CNKSR2 |  |  |
|  | CCDC22 |  |  |
|  | MIR561 |  |  |
|  | VPS13A |  |  |
|  | LRIF1 |  |  |
|  | BMP10 |  |  |
|  | ACER2 |  |  |
|  | TNFRSF10B |  |  |
|  | SIX2 |  |  |
|  | MIR4492 |  |  |
|  | PSMB11 |  |  |
|  | VAMP3 |  |  |
|  | BTC |  |  |
|  | RBBP5 |  |  |
|  | UNC5D |  |  |
|  | TSR3 |  |  |
|  | FAM111B |  |  |
|  | SNHG12 |  |  |
|  | ALG5 |  |  |
|  | SKP1 |  |  |
|  | SNX13 |  |  |
|  | CD5L |  |  |
|  | MAPK8IP1 |  |  |
|  | TTTY15 |  |  |
|  | DRAP1 |  |  |
|  | GRK6 |  |  |
|  | MED11 |  |  |
|  | GPRC5A |  |  |
|  | GLT8D1 |  |  |
|  | ERAP2 |  |  |
|  | ATP13A3 |  |  |
|  | LINC00458 |  |  |
|  | ASPRV1 |  |  |
|  | MMP26 |  |  |
|  | GID8 |  |  |
|  | HLA-DRB6 |  |  |
|  | RILP |  |  |
|  | CCNO |  |  |
|  | ZBTB16 |  |  |
|  | DNTT |  |  |
|  | PROZ |  |  |
|  | FKBP6 |  |  |
|  | TANK |  |  |
|  | MAN2A1 |  |  |
|  | CYS1 |  |  |
|  | LOC130063650 |  |  |
|  | EPSTI1 |  |  |
|  | POU2AF1 |  |  |
|  | DNM3 |  |  |
|  | RHOD |  |  |
|  | IGSF3 |  |  |
|  | ADPRH |  |  |
|  | UNC50 |  |  |
|  | CEP83 |  |  |
|  | KDM6B |  |  |
|  | MTCH1 |  |  |
|  | TACR2 |  |  |
|  | ZNF518A |  |  |
|  | DDX49 |  |  |
|  | EPB41L3 |  |  |
|  | CCDC103 |  |  |
|  | NPLOC4 |  |  |
|  | SLIT2 |  |  |
|  | LOC126806252 |  |  |
|  | ADAM19 |  |  |
|  | NELL2 |  |  |
|  | SYNJ2 |  |  |
|  | PSMD14 |  |  |
|  | PLCL2 |  |  |
|  | CDC25A |  |  |
|  | ANO1 |  |  |
|  | SLC34A2 |  |  |
|  | ENPP3 |  |  |
|  | IGHV3-72 |  |  |
|  | KCNT2 |  |  |
|  | MAPRE3 |  |  |
|  | LINC01150 |  |  |
|  | SLF1 |  |  |
|  | LOC129931648 |  |  |
|  | TOPBP1 |  |  |
|  | IPO13 |  |  |
|  | CAPN2 |  |  |
|  | IFITM10 |  |  |
|  | HLX |  |  |
|  | ANKRD24 |  |  |
|  | NUP88 |  |  |
|  | PYGO2 |  |  |
|  | LTB4R2 |  |  |
|  | INA |  |  |
|  | MIMT1 |  |  |
|  | HYAL2 |  |  |
|  | ZMYND11 |  |  |
|  | CHCHD4 |  |  |
|  | SH3KBP1 |  |  |
|  | P2RY4 |  |  |
|  | UGGT2 |  |  |
|  | MIR302C |  |  |
|  | GOLGA3 |  |  |
|  | IK |  |  |
|  | PNN |  |  |
|  | RAB43 |  |  |
|  | ZMYND10 |  |  |
|  | GRIN2C |  |  |
|  | MIR4804 |  |  |
|  | MIR1207 |  |  |
|  | H4C9 |  |  |
|  | DENND2B |  |  |
|  | PI4K2A |  |  |
|  | MAPK6 |  |  |
|  | HBM |  |  |
|  | WASHC2C |  |  |
|  | DR1 |  |  |
|  | ATP1B1 |  |  |
|  | NFIB |  |  |
|  | FRMPD4 |  |  |
|  | DHX34 |  |  |
|  | RMND5A |  |  |
|  | HEG1 |  |  |
|  | MSTO1 |  |  |
|  | INTS8 |  |  |
|  | NRF1 |  |  |
|  | LOC130063648 |  |  |
|  | NEU3 |  |  |
|  | PPM1F |  |  |
|  | HTRA2 |  |  |
|  | LOC130065345 |  |  |
|  | SELENOF |  |  |
|  | TRIM36 |  |  |
|  | NFE2 |  |  |
|  | TLN2 |  |  |
|  | RMDN2 |  |  |
|  | LINC01587 |  |  |
|  | UBE2E1 |  |  |
